# Multifunctional roles and advances of polymers in solar cell technologies: a review

**DOI:** 10.1039/d5ra05820a

**Published:** 2025-09-29

**Authors:** Nouf K. Al-Saleem, Aishah Al-Naghmaish, Mohamed Madani, Wadiah Alfawwar, A. M. Elbasiony, Salha Alharthi, Md Azizul Haque, Mohamed Mohamady Ghobashy

**Affiliations:** a Department of Physics, College of Science and Humanities-Jubail, Imam Abdulrahman Bin Faisal University Jubail 35811 Saudi Arabia; b Department of Chemistry, College of Science, Northern Border University (NBU) Arar Saudi Arabia; c Chemistry Department, College of Science, Imam Abdulrahman Bin Faisal University P.O. Box 1982 Dammam 31441 Saudi Arabia snalharthi@iau.edu.sa; d Department of Biotechnology, Yeungnam University Gyeongbuk 38541 Republic of Korea; e Radiation Research of Polymer Department, National Center for Radiation Research and Technology (NCRRT), Atomic Energy Authority P.O. Box. 29 Nasr City Cairo Egypt Mohamed_ghobashy@yahoo.com

## Abstract

The integration of polymeric materials into solar cell technologies has emerged as a transformative approach to address the limitations of conventional rigid photovoltaic systems while enabling new functionalities and applications. This comprehensive review examines the multifunctional contributions of polymers across all components of solar cell architectures, from flexible substrates to innovative protective coatings. A critical evaluation of polymer applications reveals significant progress in organic photovoltaics, where donor–acceptor copolymers have enabled power conversion efficiencies (PCEs) exceeding 18% in single-junction devices. In perovskite solar cells, polymeric hole-transport materials and encapsulation systems have demonstrated comparable performance to expensive alternatives, while offering enhanced thermal stability and reduced costs. Advanced encapsulation polymers based on polyolefin elastomers have demonstrated superior UV resistance and reduced potential-induced degradation compared to traditional ethylene-vinyl acetate systems. Innovative coatings that incorporate superhydrophobic and anti-reflective properties have demonstrated the ability to maintain over 95% of their initial power output after 12 months of outdoor exposure, representing a 10% improvement over uncoated modules. This review offers critical insights for researchers and industry practitioners seeking to advance polymer-enabled solar technologies, providing both a fundamental understanding and practical guidance for materials selection, device design, and manufacturing optimization.

## Abstract 2

Polymers enable flexible, lightweight solar cells with >18% efficiency. Encapsulants boost UV and moisture resistance. Self-cleaning coatings retain 95% output, and stretchable polymers allow wearable modules with up to 50% strain.

## Graphical abstract

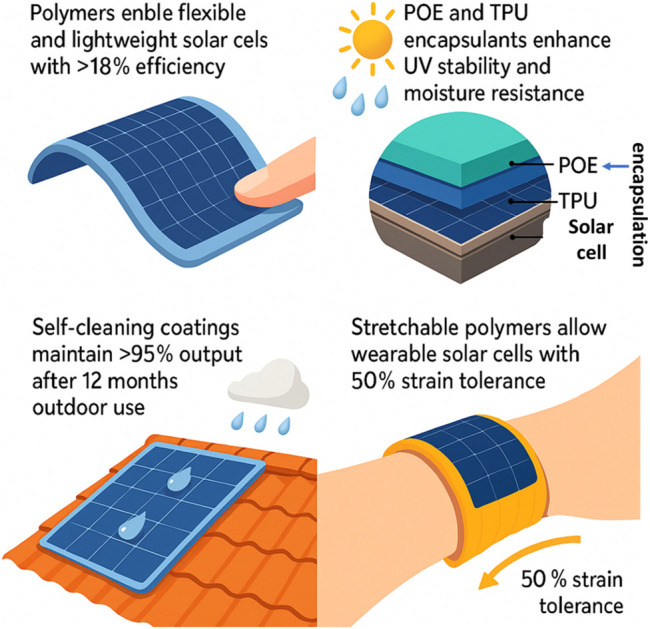

## 1. Introduction

The global transition toward renewable energy sources has intensified research efforts in photovoltaic (PV) technologies, with solar cells emerging as a cornerstone of sustainable energy infrastructure. The International Energy Agency projects that solar photovoltaics will become the world's largest source of electricity by 2050, necessitating continued innovation in materials science and device engineering.^1^ Traditional solar cell technologies, predominantly based on crystalline silicon with glass substrates and metallic components, face inherent limitations including rigidity, weight, and manufacturing constraints that restrict their deployment in emerging applications such as building-integrated photovoltaics, wearable electronics, and aerospace systems.^2,3^

Polymeric materials have emerged as transformative components in solar cell technologies, offering unprecedented opportunities to overcome these limitations while enabling new functionalities. Unlike conventional inorganic materials, polymers provide unique advantages, including mechanical flexibility, lightweight characteristics, solution processability, and tunable optical and electronic properties.^4,5^ Recent advances in polymer chemistry have yielded materials with remarkable properties: transparent conductive polymers achieving sheet resistances below 100 Ω sq^−1^ while maintaining >85% optical transparency, flexible substrates capable of withstanding >10 000 bend cycles, and encapsulation systems providing a water vapor transmission rate (WVTR) as low as 10^−6^ g m^−2^ per day.^6,7^

In organic photovoltaics (OPV), advancements in polymer technology have led to significant improvements in efficiency.

The development of donor–acceptor copolymers—such as those in the PTB7 and PM6 families—has driven (PCEs) beyond 20% in single-junction organic solar cells, particularly through the incorporation of next-generation non-fullerene acceptors and optimized BHJ morphologies.^8–10^ More recently studies reported similarly high performance in donor–acceptor systems supported by finely engineered morphologies and interfacial tuning.^11–14^ These polymers incorporate electron-rich and electron-poor units in their backbone structure, creating optimal energy level alignment and charge transport properties. Recent breakthroughs in non-fullerene acceptors, particularly Y6-based systems, have revolutionized OPV performance by extending light absorption into the near-infrared region while maintaining high open-circuit voltages.^15,16^

The emergence of perovskite solar cells (PSCs) has created new opportunities for polymer integration. Polymeric hole transport materials (HTMs) have shown promise as alternatives to expensive small-molecule HTMs, such as Spiro-OMeTAD. For instance, PTAA (poly[bis(4-phenyl)(2,4,6-trimethylphenyl)amine]) has demonstrated comparable performance while offering improved thermal stability and reduced material costs.^17,18^ Polymer encapsulation strategies for PSCs have become critical for commercial viability, with ethylene vinyl acetate (EVA) and polyolefin elastomer (POE) systems providing essential protection against moisture and oxygen ingress.^19,20^

Encapsulation represents one of the most mature applications of polymers in solar cell technologies. EVA has dominated the market for decades, providing excellent optical clarity, adhesion properties, and cost-effectiveness. However, recent research has identified limitations including potential-induced degradation (degradation pathways) and acetic acid evolution under thermal stress.^21,22^ Advanced encapsulants based on POE and thermoplastic polyurethane (TPU) have emerged as superior alternatives, offering enhanced UV stability, reduced water vapor transmission, and improved electrical insulation properties.^23,24^

The development of smart coatings represents an emerging frontier in polymer-based solar cell technologies. Anti-reflective (AR) coatings based on fluorinated polymers have achieved broadband reflection reduction through precise refractive index control, with some systems demonstrating <1% average reflectance across the solar spectrum.^25,26^ Self-cleaning surfaces inspired by natural systems have been developed using superhydrophobic polymer coatings, showing potential for reducing maintenance requirements in dusty environments.^27,28^ Recent studies have demonstrated that polymer-based anti-soiling coatings can maintain more than 95% of their initial power output after 12 months of outdoor exposure, compared to 85% for uncoated modules.^29^

Conductive polymers have found applications in transparent electrodes and charge collection layers. Poly(3,4-ethylenedioxythiophene):poly(styrenesulfonate) (PEDOT:PSS) remains the most widely used conductive polymer, with optimized formulations achieving sheet resistances below 30 Ω sq^−1^ and work function tunability from 4.2 to 5.2 eV.^30,31^ Recent advances in post-treatment methods, utilizing ionic liquids and organic solvents, have further enhanced conductivity while maintaining transparency and film quality.^32,33^

The integration of nanostructured polymers has opened up new avenues for optimizing light management and charge transport. Polymer-nanoparticle composites incorporating plasmonic nanoparticles have demonstrated enhanced light trapping, with polymers embedded with silver nanoparticles showing improvements of 15–20% in short-circuit current density.^34,35^ Hierarchical nanostructures created through polymer self-assembly have enabled precise control over charge transport pathways, leading to improved fill factors and reduced recombination losses.^36,37^

Manufacturing scalability remains a critical consideration for polymer-based solar cell technologies. Roll-to-roll (R2R) processing has demonstrated compatibility with various polymer systems, with printing speeds exceeding 10 m min^−1^ achieved for organic photovoltaic modules.^38,39^ Quality control challenges associated with polymer processing, including thickness uniformity and defect density, have been addressed through the use of advanced process monitoring and feedback control systems.^40,41^

Despite significant advances, several challenges persist in polymer-based solar cell technologies. Thermal stability limitations restrict processing temperatures and operating conditions, particularly for organic polymers.^42^ Long-term degradation mechanisms, including photo-oxidation and thermal decomposition, continue to impact device lifetime.^43,44^ The intrinsic trade-off between conductivity and transparency in conductive polymers arises from the fact that improving electrical conductivity generally requires higher levels of doping, which introduces additional charge carriers but simultaneously increases optical absorption in the visible range. As a result, heavily doped polymers exhibit strong electronic transitions that reduce transparency, whereas maintaining high transparency necessitates lower doping levels that compromise conductivity. This interdependence between optical and electronic properties remains a critical challenge, restricting the use of conductive polymers in high-efficiency optoelectronic devices.^45,46^

Environmental considerations have become increasingly important in the selection of polymers and the design of devices. Life cycle assessment studies have highlighted the environmental benefits of polymer-based flexible solar cells, particularly in terms of reduced material consumption and manufacturing energy requirements.^47,48^ The development of bio-based and biodegradable polymers offers potential pathways toward more sustainable solar cell technologies.^49,50^

Looking toward the future, several research directions show particular promise. Machine learning approaches are being employed to accelerate polymer discovery and optimization, with algorithms capable of predicting polymer properties from molecular structure.^51,52^ Artificial intelligence-guided synthesis has identified novel polymer compositions with enhanced performance characteristics.^53,54^ The integration of Internet of Things (IoT) capabilities into polymer-based solar cells promises new functionalities, including real-time performance monitoring and predictive maintenance.^55,56^

This review provides a comprehensive analysis of polymers in solar cell technologies, examining their multifunctional roles from substrates to smart coatings. Through critical evaluation of recent advances and systematic comparison with traditional materials, we identify key challenges and opportunities for future development. The analysis encompasses performance metrics, manufacturing considerations, and sustainability aspects, providing insights for researchers and industry practitioners working toward the next generation of polymer-enabled solar cell technologies.

**Fig. 1:**
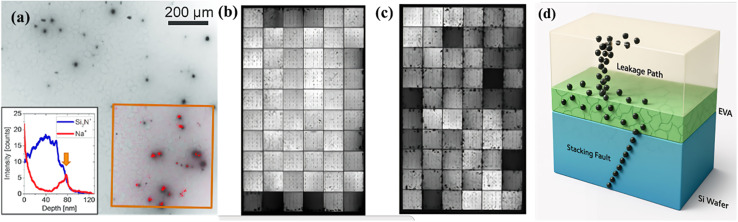
Potential-induced degradation (PID) mechanisms and testing methodologies in crystalline silicon solar cells. (a) EBIC image of monocrystalline cell region showing PID-shunts as dark circular spots with ToF-SIMS overlay (red dots) indicating sodium accumulation at SiN_*x*_/Si interface; inset shows depth profile of Na concentration at shunt sites. (b) Electroluminescence image of the module after chamber PID testing, showing an edge-initiated degradation pattern typical of field conditions. (c) Electroluminescence image after Al foil PID testing, displaying randomly distributed degraded cells across the module surface. Reproduced from ref. 57 with permission from Elsevier, (2021).

Fig. 1 demonstrate excellent scientific coherence and complementary perspectives on PID phenomena in silicon solar cells. Fig. 1a presents a detailed microscopic analysis of potential-induced degradation (PID) mechanisms in monocrystalline silicon solar cells through EBIC imaging combined with ToF-SIMS chemical analysis. The image reveals individual PID-shunts as dark circular spots with diameters ranging from 5 to 20 μm, each representing a localized disruption of the p–n junction. The red dots overlaid on the EBIC image show the precise spatial distribution of sodium ions at the SiN_*x*_/Si interface, demonstrating a direct correlation between sodium accumulation and shunt locations. The inset depth profile graph confirms significant sodium enrichment at the SiN_*x*_/Si interface, as indicated by the peak in the Na signal coinciding with the decline in Si_2_N^+^ intensity. This microscopic investigation establishes that PID-shunts are fundamentally associated with stacking faults in crystallographic planes that become decorated with migrated sodium ions, creating conductive pathways that bypass the regular junction operation.

Fig. 1b demonstrates the macroscopic manifestation of PID degradation through electroluminescence imaging of modules subjected to chamber PID testing under controlled humidity and temperature conditions. The degradation pattern exhibits a characteristic progression that initiates from the module frame and spreads inward, resulting in a distinctive edge-dominant degradation profile. This pattern reflects the realistic field conditions where moisture ingress and ionic migration typically begin at the module periphery where sealing may be compromised. The chamber PID test methodology incorporates environmental factors, such as humidity and high temperature, which are commonly employed to simulate accelerated aging conditions, as they significantly exacerbate degradation mechanisms, including hydrolysis, ion migration, and thermal decomposition. Conversely, environments with low humidity and reduced temperature can slow down these degradation pathways, thereby extending the effective operational lifetime compared to accelerated testing conditions. The edge-initiated degradation observed in this figure is consistent with field observations and demonstrates how the microscopic sodium migration mechanism identified in Fig. 1a translates to predictable macroscopic performance losses.

Fig. 1c presents an alternative degradation pattern resulting from Al foil PID testing, where the affected cells appear as randomly distributed dark squares across the module surface rather than following the edge-dominant pattern. This uniform distribution results from the Al foil creating a conductive layer that provides equivalent electrical stress conditions across the entire module surface, simulating the effect of conductive soiling or surface contamination. While this testing method produces higher degradation rates at the same stress temperature and requires less stringent humidity control, it may not fully represent typical field conditions. The random distribution pattern, though less common in field applications, can occasionally occur in real installations due to conductive soiling or specific encapsulation material properties that create uniform potential distribution across the module surface.

## 2. Polymer classification in solar cell technologies

Polymers are indispensable in the advancement of modern solar cell technologies due to their multifunctional roles, lightweight nature, and flexibility. These polymeric materials are typically categorized into three main functional groups within photovoltaic (PV) devices: structural components, functional components, and protective components—each fulfilling a distinct purpose to optimize device performance and durability.^58^ Structurally, polymers provide the necessary mechanical framework and support for solar cells, particularly those designed to be flexible or lightweight. Commonly used flexible substrates include polyethylene terephthalate (PET) and polyethylene naphthalate (PEN), which offer the mechanical pliability needed for bendable solar modules. Transparent polymers, such as polyimide (PI), serve as durable substrates, allowing sunlight to reach the active layers without compromising the device's structural integrity. Complementing the substrates, encapsulant polymers such as ethylene-vinyl acetate (EVA) and polyvinyl butyral (PVB) encase the inner layers of solar cells, protecting them from moisture, oxygen, and other environmental factors that could degrade their performance over time.^59^ These encapsulants are crucial for ensuring the device's longevity, as they require high optical clarity, flexibility, and excellent barrier properties. In addition to their structural roles, functional polymers play a central role in the operation of the solar cell by actively participating in charge generation, separation, transport, and collection. Within this category, charge transport layers are essential—hole transport layers (HTLs), for instance, often use conductive polymers like PEDOT:PSS to facilitate the efficient movement of holes (positive charge carriers) to the anode.^60^ Similarly, electron transport layers (ETLs) commonly utilize polymers such as PCBM ([6,6]-phenyl-C_61_-butyric acid methyl ester), which efficiently transport electrons to the cathode.^61^ The active layer of the solar cell, responsible for absorbing sunlight and generating excitons (electron–hole pairs), typically consists of a blend of donor and acceptor materials. Donor polymers like poly(3-hexylthiophene) (P3HT) and PTB7 (polythieno[3,4-*b*]-thiophene-*co*-benzodithiophene) possess extended conjugation and suitable energy levels to facilitate photon absorption and exciton generation.^62^ Acceptor materials, initially dominated by fullerene derivatives, have recently shifted towards more efficient non-fullerene acceptors (NFAs) such as ITIC and Y6, which offer improved charge separation, broader absorption spectra, and enhanced device efficiencies. Furthermore, conductive polymers such as polyaniline (PANI) and PEDOT:PSS are frequently employed as transparent or flexible electrodes, replacing traditional brittle materials like indium tin oxide (ITO) in flexible electronics.^63^ Interconnects composed of conductive polymers also play a crucial role in integrating multiple cells and efficiently collecting charge carriers. Protective polymers, the third major category, are designed to defend the solar cell from physical and environmental damage, thus prolonging its operational life and efficiency. Anti-reflective coatings made from fluorinated polymers are engineered to possess refractive indices that minimize surface reflectance, thereby enhancing the absorption of incident light. Self-cleaning surfaces, which utilize hydrophobic polymers such as polydimethylsiloxane (PDMS), help prevent the accumulation of dust, water, and other contaminants that could block light and reduce efficiency.^64^ These materials often exhibit lotus-leaf-like properties, enabling water droplets to roll off the surface, carry away particles, and maintain surface cleanliness. In addition, exposure to ultraviolet (UV) radiation from sunlight poses a significant threat to organic solar cell components. To combat this, UV-blocking polymers like polycarbonates are used to shield sensitive layers from photodegradation. These UV-protective layers must balance transparency in the visible spectrum with high UV absorption to ensure the continued functionality of the active materials. Taken together, the careful selection and engineering of polymers for these various roles—whether for structure, function, or protection—are key to the design of high-performance solar cells. Recent innovations in polymer chemistry, such as the development of low-bandgap donor polymers, highly crystalline NFAs, and smart self-healing encapsulants, have significantly boosted the efficiency and stability of organic and hybrid solar cells. Moreover, the recyclability and potential biodegradability of certain polymeric materials contribute to the environmental sustainability of photovoltaic technologies. These advances make polymers not only vital components but also dynamic enablers of next-generation solar cells, particularly for applications that demand lightweight, conformable, and portable power solutions such as wearable electronics, building-integrated photovoltaics (BIPVs), and roll-to-roll printed solar panels.^65^ As the field progresses, ongoing research continues to focus on enhancing the electrical, optical, and mechanical properties of polymers, ensuring that they meet the demanding operational conditions of real-world solar energy systems. From enhancing charge mobility and environmental resistance to facilitating large-area manufacturability, polymers are at the heart of efforts to make solar energy more efficient, affordable, and accessible. Therefore, the classification of polymers into structural, functional, and protective categories provides a framework for understanding their multifaceted contributions to solar cell technology, underscoring their irreplaceable role in driving the performance, stability, and commercial viability of modern photovoltaic devices (Table 1).

**Table 1:** Comprehensive properties of polymer substrates for solar cell applications

Material	*T*_g_ (°C)	*T*_m_ (°C)	Transparency (%)	Tensile strength (MPa)	Elastic modulus (GPa)	CTE (ppm/°C)	WVTR (g m^−2^ per day)	OTR (cm^2^ per day)	Bend radius (mm)
Polyethylene terephthalate (PET)	78	255	89	55–75	2.8–4.1	70	15–20	5–15	2–5
Polyethylene naphthalate (PEN)	120	269	86	180–220	5.2–6.8	20	8–12	3–8	1–3
Polyimide (PI)	350–400	>500	85–92	120–340	2.5–8.5	12–20	5–15	2–10	0.5–2
Polycarbonate (PC)	145	267	89–91	55–75	2.0–2.4	65–70	25–35	15–25	5–10
Cyclic olefin copolymer (COC)	70–180	N/A	92–95	50–80	2.3–3.2	60–80	0.1–1.0	5–20	3–8
Polyethersulfone (PES)	225	N/A	84–87	84–90	2.6–2.8	54–56	12–18	8–15	2–5
Polysulfone (PSU)	190	N/A	86–88	70–85	2.3–2.7	56–60	10–15	6–12	3–6
Polyetherimide (PEI)	217	N/A	85–89	105–115	3.2–3.4	47–56	8–12	4–8	2–4
Fluorinated ethylene propylene (FEP)	80	260	95–97	20–25	0.5–0.6	135–200	2–5	10–30	1–3
Perfluoroalkoxy (PFA)	90	305	95–98	20–35	0.5–0.7	100–135	1–3	5–20	1–2
Ethylene tetrafluoroethylene (ETFE)	95	267	94–96	40–50	0.8–1.6	70–100	3–8	15–40	2–5
Polyvinylidene fluoride (PVDF)	−35	177	90–92	35–55	1.2–2.8	130–140	5–15	20–50	3–8
Polytetrafluoroethylene (PTFE)	130	327	96–98	20–35	0.4–0.5	100–200	1–2	5–15	5–15
Cyclic olefin polymer (COP)	130–180	N/A	92–95	55–75	2.0–3.5	60–70	0.05–0.5	3–15	2–6
Polyarylate (PAR)	190	N/A	87–90	80–110	2.1–2.8	50–60	12–20	8–18	2–5
Liquid crystal polymer (LCP)	280–340	N/A	85–88	140–200	12–18	2–17	5–12	3–10	1–3
Polyoxymethylene (POM)	−85	175	75–80	60–70	2.6–3.1	100–110	20–30	40–80	8–15
Polyphenylene oxide (PPO)	210	N/A	88–91	55–75	2.3–2.6	50–60	8–15	10–20	3–7
Polyphenylene sulfide (PPS)	85	285	82–86	65–100	3.3–4.1	50–60	6–12	5–15	2–5
Thermoplastic polyurethane (TPU)	−50 to 80	180–220	85–90	35–60	0.1–0.8	150–200	500–2000	200–800	0.5–2
Polyvinyl alcohol (PVA)	85	230	88–92	75–120	2.0–6.0	70–100	1000–5000	100–500	5–12
Cellulose acetate (CA)	105	230	89–92	35–55	1.2–4.0	80–120	400–1500	150–600	3–8
Polylactic acid (PLA)	55–60	175	88–91	50–70	3.0–4.0	60–68	200–800	100–400	5–12
Polyhydroxybutyrate (PHB)	5	175	85–89	40–50	3.5–4.0	150–200	300–1000	200–600	8–15
Polystyrene (PS)	100	240	90–92	35–55	3.0–3.5	60–80	50–150	300–800	10–20
Polymethyl methacrylate (PMMA)	105	N/A	92–94	65–75	2.2–3.2	70–90	15–30	50–200	5–15
Polyvinyl chloride (PVC)	82	212	85–88	50–80	2.4–4.1	50–100	20–50	30–100	8–20
Polypropylene (PP)	−10	165	90–92	30–40	1.1–1.6	100–150	5–15	2000–5000	5–15
Polyethylene (PE)	−125	135	90–93	20–35	0.2–1.4	100–200	8–20	3000–8000	2–10
Poly(4-vinylphenol) (PVP)	150	N/A	87–90	45–65	2.5–3.8	55–75	100–300	80–250	3–8
Polyacrylonitrile (PAN)	85	317	82–87	350–630	15–35	60–80	50–150	25–100	2–6
Polyvinylfluoride (PVF)	40	200	93–95	70–140	2.1–2.8	50–80	2–8	8–25	2–5
Polyvinyl butyral (PVB)	50–70	N/A	88–91	20–35	0.02–2.0	80–150	200–800	100–400	1–5
Ethylene vinyl acetate (EVA)	−30	85	91–94	10–25	0.02–0.2	200–300	80–200	500–1500	0.5–3
Thermoplastic elastomer (TPE)	−50 to 50	150–200	85–92	15–45	0.1–1.0	100–250	100–1000	200–2000	0.2–2

### 2.1. Structural polymers in solar cells

Structural polymers form the physical backbone of solar cell technologies, particularly in flexible and lightweight devices, where conventional rigid materials such as glass or metal are unsuitable. These polymers are primarily responsible for providing mechanical support, dimensional stability, and overall robustness to the photovoltaic architecture, while also often contributing to the device's optical or barrier properties. The two main classes of structural polymer components in solar cells are substrates and encapsulants, both of which are indispensable for the fabrication and longevity of modern photovoltaic devices, particularly organic solar cells (OSCs), perovskite solar cells (PSCs), and other thin-film solar technologies.^66^ Substrates are the foundational layers upon which the entire solar cell stack is built. In conventional silicon-based solar cells, glass is typically used as the substrate. Still, in flexible and wearable solar cells, polymeric materials are favoured due to their low weight, flexibility, and cost-effective processing. Polyethylene terephthalate (PET) and polyethylene naphthalate (PEN) are widely used thermoplastic substrates due to their excellent mechanical strength, dimensional stability, and compatibility with large-scale roll-to-roll processing techniques. PET is particularly favored for its affordability and transparency, while PEN offers superior thermal resistance, which is crucial during high-temperature processing steps. Another common substrate is polyimide (PI), which provides high optical transparency and excellent thermal and chemical stability, making it suitable for advanced photovoltaic devices that require high-temperature post-processing. PI's high flexibility also makes it ideal for foldable or wearable solar applications. The choice of substrate has a significant influence on the overall device performance, including light transmittance, mechanical robustness, and environmental stability.^67^

Encapsulants, on the other hand, serve to physically seal and protect the internal layers of the solar cell from environmental stressors such as moisture, oxygen, UV radiation, dust, and mechanical abrasion. These environmental factors are known to degrade sensitive layers such as the photoactive layer or the electrode materials, especially in organic and perovskite solar cells, which are more chemically vulnerable than their inorganic counterparts. Ethylene-vinyl acetate (EVA) is the most commonly used encapsulant in commercial photovoltaic modules, particularly in crystalline silicon solar cells.^68^ EVA offers excellent adhesion, high optical transparency, and sufficient thermal stability. However, it may degrade over time, particularly under high UV exposure, producing acetic acid, which can corrode other cell components. To address this, other polymers, such as polyvinyl butyral (PVB) and thermoplastic polyolefin (TPO), have been explored, especially in newer or more sensitive cell architectures. PVB, for example, exhibits superior adhesion to glass and offers better resistance to moisture ingress, making it suitable for devices where high barrier properties are critical. In advanced applications, multilayer encapsulant structures incorporating barrier coatings, such as SiO_*x*_ or Al_2_O_3_, are deposited on polymer films (*e.g.*, PET or PI) to improve hermeticity while retaining flexibility. Together, substrates and encapsulants define the physical form and durability of the solar cell. The interplay between flexibility, transparency, thermal tolerance, and barrier properties must be finely tuned for optimal performance.

Furthermore, structural polymers must also be compatible with the solvents, temperatures, and mechanical stresses involved in solar cell fabrication and operation. With the rapid development of flexible and wearable electronics, the demand for polymeric substrates and encapsulants with advanced functionalities—such as self-healing, recyclability, or stretchability—is increasing. Research is actively exploring new polymer composites and nanostructured films that combine mechanical strength with additional properties, such as UV filtering, enhanced gas barrier performance, or even active functionalities like thermochromic behaviour. In conclusion, structural components made from advanced polymeric materials are foundational to the physical and operational integrity of solar cells, particularly in flexible and lightweight formats. They ensure that the device maintains mechanical stability during handling, transportation, and deployment, while also protecting the sensitive internal components from the deleterious effects of environmental exposure, thus playing a crucial role in enabling durable, efficient, and practical solar energy technologies.

#### 2.1.1. Substrates (flexible, transparent)

The development of flexible and transparent substrates represents a cornerstone advancement in polymer-enabled solar cell technologies, fundamentally transforming device architectures and enabling new application domains.^69^ Traditional rigid glass substrates, while offering excellent optical clarity and chemical stability, impose significant limitations on device flexibility, weight, and manufacturing scalability. Polymeric substrates have emerged as compelling alternatives, offering unique combinations of mechanical flexibility, optical transparency, and processability that enable roll-to-roll manufacturing at unprecedented scales.^70^

Polyethylene terephthalate (PET) and polyethylene naphthalate (PEN) have dominated the flexible substrate landscape due to their exceptional mechanical properties and thermal stability.^71^ These materials demonstrate remarkable durability, withstanding over 20 000 bend cycles while maintaining structural integrity and optical performance. The superior glass transition temperature of PEN (120 °C) compared to PET (78 °C) provides enhanced thermal stability during device processing, enabling compatibility with higher-temperature fabrication steps. However, the processing temperature limitations of most polymeric substrates, typically restricted to below 200 °C, represent a significant constraint for specific device architectures and manufacturing processes.^72^

Polyimide substrates have garnered significant attention for high-performance applications that require enhanced thermal stability.^73^ These materials can withstand processing temperatures exceeding 300 °C while maintaining excellent mechanical flexibility and dimensional stability. The inherent yellowish coloration of traditional polyimides has been addressed through the development of colorless polyimide formulations, which achieve optical transmission exceeding 85% in the visible spectrum while retaining superior thermal properties. Advanced polyimide chemistries incorporating fluorinated segments have demonstrated improved optical clarity and reduced water absorption, both of which are critical factors for long-term device reliability.^74^

The emergence of ultra-thin glass substrates, although not strictly polymeric, represents a significant development in flexible substrate technology.^75^ These materials, typically 25–100 μm thick, offer the chemical and thermal stability of conventional glass while providing mechanical flexibility approaching that of polymers. Hybrid approaches combining ultra-thin glass with polymeric support layers have shown promise in achieving optimal combinations of flexibility, thermal stability, and processing compatibility.

Surface modification of polymeric substrates has proven essential for optimal device performance.^76^ Plasma treatment, chemical etching, and coating deposition are commonly employed to enhance surface energy, improve adhesion, and reduce surface roughness. These modifications are particularly critical for organic photovoltaic devices where interfacial quality directly impacts charge transport and device efficiency. Advanced surface treatment techniques have achieved root-mean-square roughness values of below 1 nm, comparable to those of glass substrates.

#### 2.1.2. Encapsulants (barrier properties, durability)

Encapsulation systems represent critical components in polymer-enabled solar cell technologies, providing essential protection against environmental degradation while maintaining optical transmission and mechanical integrity.^77^ The development of advanced polymeric encapsulants has addressed fundamental challenges in solar cell durability, particularly moisture ingress, oxygen permeation, and ultraviolet degradation that can severely compromise device performance and lifetime.

Ethylene vinyl acetate (EVA) has historically served as the standard encapsulation material in photovoltaic modules, offering good optical clarity, adhesion properties, and processability.^78^ However, EVA systems exhibit several limitations, including thermal degradation at elevated temperatures, potential-induced degradation under high-voltage conditions, and the evolution of acetic acid during thermal cycling. These limitations have driven the development of advanced encapsulation systems based on polyolefin elastomers (POE) and thermoplastic polyurethanes (TPU) that demonstrate superior thermal stability and reduced degradation pathways.^79^

Ultra-barrier encapsulation systems have achieved remarkable performance metrics, with water vapor transmission rates as low as 10^6^ g m^−2^ s^−1^, representing an order of magnitude improvement over conventional materials.^80^ These systems typically employ multilayer architectures that combine organic and inorganic barrier layers, where polymeric components provide mechanical flexibility and adhesion, while inorganic layers contribute primary barrier functionality. Atomic layer deposition techniques have enabled the fabrication of ultra-thin inorganic barriers with exceptional uniformity and pinhole-free coverage.

The development of self-healing encapsulation materials represents a significant advancement in durability enhancement.^81^ These systems incorporate microcapsules containing healing agents that are released upon mechanical damage, allowing for the autonomous repair of barrier defects. Advanced formulations have demonstrated the ability to maintain barrier properties even after multiple damage-healing cycles, potentially extending device lifetimes significantly beyond conventional systems.

Superhydrophobic encapsulation surfaces have emerged as practical approaches for maintaining solar cell performance under outdoor conditions.^82^ These surfaces, typically incorporating micro- and nanostructured morphologies with low surface energy coatings, demonstrate water contact angles exceeding 150° and enable self-cleaning functionality. Field testing has been shown that superhydrophobic coatings can maintain over 95% of their initial power output after 12 months of outdoor exposure, representing a 10% improvement over uncoated modules due to reduced soiling and enhanced light management.

Anti-reflective properties integrated into encapsulation systems provide additional performance benefits through enhanced light coupling and reduced surface reflection losses.^83^ Nanostructured surface morphologies, graded refractive index coatings, and multilayer interference systems have been successfully incorporated into polymeric encapsulants. These approaches typically achieve reflection losses of less than 2% across the solar spectrum while maintaining mechanical durability and weathering resistance.

The long-term stability of polymeric encapsulants under UV exposure remains a critical consideration for commercial deployment.^84^ Advanced UV-resistant formulations incorporating hindered amine light stabilizers, UV absorbers, and antioxidant systems have demonstrated significantly improved photostability compared to unprotected polymers. Accelerated weathering tests using concentrated UV exposure have demonstrated that optimized encapsulation systems can maintain their optical and mechanical properties for periods equivalent to over 25 years of outdoor exposure.

Recyclability and end-of-life considerations have become increasingly important in the development of encapsulation systems.^85^ Thermoplastic encapsulants offer advantages in module recycling, enabling separation and recovery of active materials through thermal processing. Advanced debondable encapsulation systems have been developed that allow reversible adhesion, facilitating component separation and material recovery at module end-of-life (Table 2).

**Table 2:** Comprehensive performance data of encapsulation systems for solar cell applications

Material	WVTR (g m^−2^ per day)	OTR (cm^2^ per day)	UV transmittance (%)	Thermal stability (°C)	Adhesion strength (N cm^−1^)	Volume resistivity (Ω cm)	Dielectric strength (kV mm^−1^)	Yellowing index	Reference
Ethylene vinyl acetate (EVA)	80–200	500–1500	85–90	150	25–45	10^14^–10^15^	40–60	2–8	Klemchuk *et al.*^86^
Polyolefin elastomer (POE)	15–50	100–400	90–95	200	30–55	10^15^–10^16^	50–80	1–3	Oreski *et al.*^79^
Thermoplastic polyurethane (TPU)	500–2000	200–800	85–92	180	40–70	10^12^–10^14^	20–40	3–10	Raman *et al.*^87^
Polyvinyl butyral (PVB)	200–800	100–400	88–91	120	20–40	10^13^–10^14^	30–50	2–6	Huang *et al.*^88^
Ionomer resin	5–25	50–200	92–95	300	50–80	10^14^–10^16^	60–100	1–2	Tracy *et al.*^89^
Polydimethylsiloxane (PDMS)	1000–5000	5000–15000	95–98	250	15–35	10^14^–10^15^	15–30	0.5–1	Leem *et al.*^90^
Fluoropolymer film (FEP)	2–5	10–30	95–97	300	10–25	10^16^–10^18^	80–120	0.2–0.5	Hirschmann *et al.*^91^
Polyethylene terephthalate (PET)	15–20	5–15	89–91	200	20–35	10^14^–10^15^	150–200	2–5	Iwan *et al.*^92^
Cyclic olefin copolymer (COC)	0.1–1.0	5–20	92–95	200	25–45	10^16^–10^17^	100–150	0.5–2	Gunasekaran *et al.*^93^
Polycarbonate (PC)	25–35	15–25	89–91	180	30–50	10^14^–10^15^	25–40	3–8	Budiman *et al.*^94^
Polyimide (PI)	5–15	2–10	85–92	400	35–60	10^15^–10^16^	200–300	1–4	Gouzman *et al.*^95^
Polyvinyl fluoride (PVF)	2–8	8–25	93–95	250	25–40	10^15^–10^16^	100–150	0.3–1	Ocaña *et al.*^96^
Polyvinylidene fluoride (PVDF)	5–15	20–50	90–92	200	20–40	10^13^–10^14^	50–80	1–3	Saxena *et al.*^97^
Multilayer barrier film (SiO_*x*_/polymer)	0.001–0.01	0.1–1	90–93	200	30–50	10^14^–10^15^	80–120	1–3	Lien *et al.*^98^
Aluminum oxide barrier (AlO_*x*_/polymer)	0.0001–0.001	0.01–0.1	88–91	250	25–45	10^15^–10^16^	100–180	0.5–2	Corsini *et al.*^99^
Polyethylene naphthalate (PEN)	8–12	3–8	86–89	220	35–55	10^14^–10^15^	180–250	2–5	Addonizio *et al.*^100^
Polyetherimide (PEI)	8–12	4–8	85–89	300	40–65	10^15^–10^16^	150–200	1–3	Ye *et al.*^101^
Polyethersulfone (PES)	12–18	8–15	84–87	280	35–55	10^14^–10^15^	120–180	2–4	Ganjali *et al.*^102^
Liquid crystal polymer (LCP)	5–12	3–10	85–88	350	50–80	10^16^–10^17^	200–300	0.5–2	Lee *et al.*^103^
Polyacrylate copolymer	50–150	100–300	90–94	150	20–40	10^13^–10^14^	30–60	2–6	Shanti *et al.*^104^
Polyurethane acrylate (PUA)	100–400	200–600	88–92	160	35–60	10^12^–10^14^	25–45	3–8	Ismail *et al.*^105^
Perfluoroalkoxy (PFA)	1–3	5–20	95–98	350	15–30	10^16^–10^18^	100–180	0.1–0.3	Nain *et al.*^106^
Ethylene tetrafluoroethylene (ETFE)	3–8	15–40	94–96	300	20–35	10^15^–10^16^	60–100	0.2–0.8	Lamnatou *et al.*^107^
Polytetrafluoroethylene (PTFE)	1–2	5–15	96–98	350	5–15	10^16^–10^18^	40–80	0.1–0.2	Nadege *et al.*^108^
Polystyrene-*block*-Polybutadiene (SBS)	200–600	800–2000	88–91	120	25–50	10^12^–10^14^	15–30	5–12	Dintcheva *et al.*^109^
Polyvinyl acetate (PVAc)	300–800	400–1200	87–90	100	15–30	10^12^–10^13^	20–40	4–10	Jiang *et al.*^110^
Polyethylene glycol diacrylate (PEGDA)	500–1500	300–1000	90–93	140	30–55	10^11^–10^13^	25–50	2–6	Xu *et al.*^111^
Polylactic acid (PLA)	200–800	100–400	88–91	120	20–40	10^13^–10^14^	40–80	3–8	Gunasekaran *et al.*^112^
Polyhydroxyalkanoate (PHA)	150–500	80–300	85–89	140	25–45	10^13^–10^14^	35–70	2–6	Wu *et al.*^113^
Cellulose acetate (CA)	400–1500	150–600	89–92	150	20–35	10^12^–10^13^	30–60	4–9	Cortina *et al.*^114^

### 2.2. Functional polymers in solar cells

The development of functional polymers for use in charge transport, light absorption, and charge collection is driving continuous improvements in solar cell performance and stability. Innovations in polymer chemistry have enabled precise control over molecular weight, energy levels, crystallinity, and phase separation behavior, all of which are crucial for achieving high efficiencies and long-term operational stability. Moreover, the compatibility of these polymers with low-temperature and solution-based processes aligns well with the goals of cost-effective, roll-to-roll manufacturing for commercial solar cell deployment.^115^ As research progresses, the emphasis is shifting toward developing multifunctional polymers that can serve dual or even triple roles (*e.g.*, light-harvesting and charge-transporting), further simplifying device architecture and reducing material costs.

Functional polymeric components are the key active materials in solar cell devices, directly responsible for light absorption, charge generation, separation, transport, and collection. These materials are primarily classified into charge transport layers (CTLs), active layer components (donors and acceptors), and electrode/interconnect polymers. Their design and integration significantly influence the photovoltaic efficiency, internal quantum yield, and overall durability of organic and hybrid solar cells, including organic photovoltaics (OPVs) and perovskite solar cells (PSCs).

Charge Transport Layers (CTLs) enable the selective extraction and transport of charges generated in the photoactive layer. They are typically divided into hole-transport layers (HTLs) and electron-transport layers (ETLs). Among HTLs, PEDOT:PSS (poly(3,4-ethylenedioxythiophene):polystyrenesulfonate) is widely employed due to its high electrical conductivity, optical transparency, solution-processability, and compatibility with flexible substrates.^116^ PEDOT:PSS facilitates efficient hole extraction from the active layer to the anode while simultaneously smoothing the underlying surface to promote better film formation.^117^ However, its acidic and hygroscopic nature may degrade the underlying layers over time, especially in PSCs, prompting the development of alternative HTLs such as poly(triarylamine) (PTAA) and polyaniline (PANI), which offer better chemical stability.^118^ For ETLs, PCBM ([6,6]-phenyl-C_61_-butyric acid methyl ester) has long been the benchmark due to its superior electron mobility, good solubility, and excellent compatibility with donor polymers.^119^ Nevertheless, newer non-fullerene ETL materials, such as conjugated polyelectrolytes or fluorinated n-type polymers, have emerged, offering more favorable band alignment and thermal stability.^120^

Active layer materials, which form the photoactive interface, are perhaps the most critical functional component in polymer solar cells. The bulk heterojunction (BHJ) concept combines electron donor and acceptor materials into an interpenetrating network to maximize the donor–acceptor interface for exciton dissociation. On the donor side, polymers such as P3HT (poly(3-hexylthiophene)) have been widely used due to their semi-crystalline nature, low cost, and decent hole mobility.^121^ More advanced donor polymers, such as PTB7 and PM6, have been developed with lower bandgaps and improved absorption profiles, enabling greater light harvesting and reduced energy losses.^15^ For acceptor materials, although fullerenes, such as PCBM, dominated early research, limitations including narrow absorption and morphological instability led to the rise of non-fullerene acceptors (NFAs). NFAs such as ITIC and Y6 have shown enhanced performance due to their broader and stronger absorption spectra, tunable energy levels, and better film-forming properties.^16^ Devices using Y6-based NFAs have exceeded 18% power conversion efficiency (PCE), a major milestone in OPV research.^15,16^

Electrodes and interconnect materials are responsible for collecting and transporting charges to the external circuit. Conventional transparent electrodes, such as indium tin oxide (ITO), offer excellent conductivity and transparency but suffer from brittleness and the scarcity of indium, making them unsuitable for flexible devices.^122^ In this context, conductive polymers such as PEDOT:PSS, PANI, and polypyrrole (PPy) have emerged as promising alternatives. Doped PEDOT:PSS can act as both an electrode and HTL, providing flexibility and compatibility with roll-to-roll printing.^116^ PANI, known for its tunable conductivity and electrochemical stability, has been incorporated into hybrid structures as flexible electrodes or interconnects.^4^ These polymers can be processed using low-temperature and solution-based techniques, aligning with the goal of scalable, low-cost fabrication. Additionally, conductive adhesives and polymer–metal hybrids are used to interconnect multiple solar cells into modules without compromising flexibility or performance.^122^ In addition to OPVs and PSCs, dye-sensitized solar cells (DSSCs) represent another class of third-generation photovoltaics where polymeric materials have significantly enhanced device performance, flexibility, and stability. Initially developed with rigid substrates and liquid electrolytes, DSSCs have evolved through the integration of polymer gel electrolytes, which reduce leakage, enhance ionic conductivity, and enable flexible and wearable applications. Polymer-based encapsulants and counter electrode composites have further improved environmental durability and reduced reliance on expensive platinum materials. Furthermore, flexible polymer substrates, such as PET or PEN, have enabled the fabrication of bendable DSSCs suitable for building-integrated photovoltaics (BIPV) and portable electronics. These advances underscore how polymer science continues to expand the material toolbox for next-generation solar energy systems, extending beyond organic and perovskite photovoltaics.

**Fig. 2:**
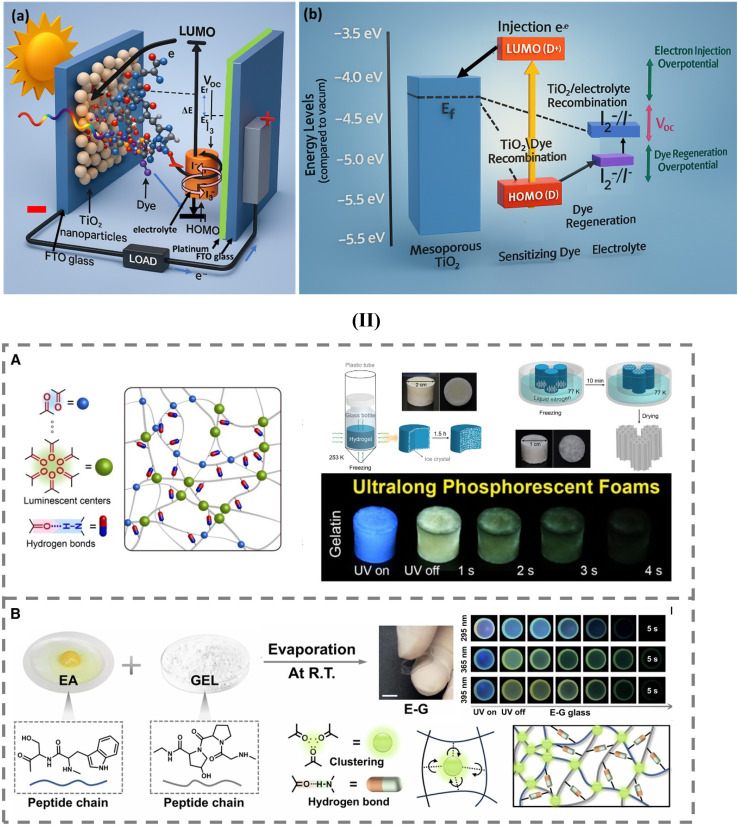
(I) Schematic of a DSSC showing light absorption by dye, electron injection into TiO_2_, and charge transport through the electrolyte and counter electrode. (b). Energy level diagram of a DSSC illustrating photoexcitation, electron injection, and potential loss due to recombination pathways. (II) Self-assembled polymer-based room-temperature phosphorescent materials. (A) Ultralong phosphorescent foams from gelatin hydrogels *via* hydrogen-bond-driven self-assembly. (B) Flexible molecular glasses (EG) with long RTP lifetime prepared by evaporation-induced self-assembly of egg albumin and gelatin. Reproduced from ref. 134 with permission from [American Chemical Society], copyright > [2021]. Reproduced from ref. 133 with permission from Nature, (2024).

Fig. 2(I)a illustrates the comprehensive architecture and operational principle of a dye-sensitized solar cell (DSSC), a photoelectrochemical device designed for converting solar energy into electrical power through the synergistic interaction of its constituent components: a mesoporous TiO_2_ photoanode, a dye sensitizer, a redox electrolyte, and a counter electrode. The core of the device is a nanocrystalline TiO_2_ layer, which has a high surface area, allowing for maximum dye loading. Upon solar irradiation, the adsorbed dye molecules absorb photons and transition from the HOMO to the LUMO, initiating photoexcitation. The excited electrons are rapidly injected into the conduction band of TiO_2_, from which they percolate through the porous network and are collected at the conducting substrate, thus generating current. Meanwhile, the oxidized dye is regenerated by accepting electrons from the I^−^/I_3_^−^ redox couple in the electrolyte. The electrolyte ensures ionic transport and maintains charge neutrality, while the counter electrode, typically platinum, facilitates the reduction of I_3_^−^ to I^−^, thereby completing the electrical circuit and enabling continuous operation.

Fig. 2(I)b delves deeper into the energy-level interactions that underpin the function of DSSC. It shows how electrons, excited by photon absorption, move from the dye's HOMO to LUMO and are subsequently injected into the conduction band of TiO_2_. This electron injection initiates charge separation, a critical step for photovoltaic activity. The photovoltage (*V*_OC_) is derived from the potential difference between the quasi-Fermi level of electrons in the TiO_2_ and the redox potential of the electrolyte. Efficient dye regeneration by the electrolyte and swift electron transport through the TiO_2_ network help minimize recombination and sustain current generation. However, recombination pathways—such as back electron transfer from TiO_2_ to the oxidized dye or the electrolyte—contribute to dark current and reduce overall efficiency. The interplay between Fig. 2(I)a and b illustrates how the structural design (*e.g.*, mesoporous TiO_2_) and energy alignment (*e.g.*, dye LUMO and TiO_2_ conduction band) must be finely tuned to optimize charge separation, transport, and collection processes, thereby enhancing the power conversion efficiency of DSSCs.

#### 2.2.1. Charge transport layers (HTL, ETL)

Charge transport layers represent fundamental components in polymer-enabled solar cell architectures, serving as selective contacts that facilitate efficient extraction of photogenerated carriers while blocking unwanted charge recombination.^123^ The development of polymeric hole transport layers (HTLs) and electron transport layers (ETLs) has revolutionized device performance, enabling the fine-tuning of energy level alignment, charge mobility, and interfacial properties that directly impact power conversion efficiency and operational stability.

(PEDOT:PSS) has emerged as the predominant polymeric hole transport material, offering exceptional processability, optical transparency, and hole mobility exceeding 10^−3^ cm^2^ V^−1^ s^−1^.^124^ The aqueous processability of PEDOT:PSS enables low-temperature fabrication compatible with flexible plastic substrates, while its work function tunability through compositional modification allows optimization for various active layer systems. However, the hygroscopic nature and inherent acidity of PEDOT:PSS can compromise long-term device stability, driving the development of alternative hole transport materials.^125^

Doped conjugated polymers have demonstrated remarkable potential as high-performance hole-transport layers, with carefully designed molecular structures enabling hole mobilities exceeding 10^−2^ cm^2^ V^−1^ s^−1^.^126^ Molecular doping strategies employing p-type dopants, such as 2,3,5,6-tetrafluoro-7,7,8,8-tetracyanoquinodimethane (F4TCNQ), have achieved conductivities exceeding 10^2^ S cm^−1^ while maintaining optical transparency of over 80% in the visible spectrum. The precise control of doping concentration enables the systematic tuning of the Fermi level position, allowing for optimal energy level alignment with active layer materials.^127^

Crosslinkable hole-transport polymers have addressed critical challenges in multilayer device fabrication, preventing the dissolution of underlying layers during subsequent processing steps.^128^ These materials typically incorporate crosslinkable functional groups that can be activated through thermal treatment or UV exposure, forming insoluble networks while maintaining charge transport properties. Advanced crosslinkable formulations have demonstrated high thermal stability, exceeding the requirements for compatibility with high-temperature processing steps necessary for specific device architectures.

Polymer electron transport layers have traditionally been more challenging to develop compared to hole transport materials, primarily due to the inherent instability of n-type organic semiconductors in ambient conditions.^129^ However, recent advances in polymer chemistry have yielded electron transport materials with remarkable air stability and electron mobilities exceeding 10^−2^ cm^2^ V^−1^ s^−1^. Polymers incorporating electron-deficient units such as perylene diimides, naphthalene diimides, and benzothiadiazole have shown particular promise for electron transport applications.^130^

The development of self-doped polymer transport layers represents a significant advancement in eliminating the need for external dopants while maintaining high conductivity.^131^ These materials incorporate ionic side chains that provide intrinsic charge compensation, enabling high conductivity without compromising film morphology or introducing mobile dopant species that can migrate during device operation. Self-doped polymers have demonstrated conductivities exceeding 10^1^ S cm^−1^ with excellent thermal and electrochemical stability.

Interface engineering through polymer transport layers has proven critical for optimizing charge extraction efficiency and minimizing interfacial recombination losses.^132^ Surface modification techniques, including plasma treatment, self-assembled monolayers, and interlayer insertion, have been employed to fine-tune interfacial energetics and improve charge collection. Advanced interface engineering approaches have achieved near-unity charge collection efficiencies across diverse active layer systems.

Fig. 2(II)A illustrates the development of ultralong phosphorescent foams derived from gelatin (GEL) hydrogels, as studied by Cai *et al.*^136^ The researchers exploited hydrogen bonding as a critical driving force for the directional alignment and self-assembly of GEL oligomers. This process enabled the formation of rigid, fibrous structures that not only provided high mechanical integrity to the resulting foams but also enhanced their room-temperature phosphorescence (RTP). Remarkably, hydrogen bonds stabilized carbonyl clusters in collagen molecules within GEL, facilitating efficient triplet exciton generation and minimizing non-radiative decay pathways. Through controlled ice crystal growth during the freezing process, the authors achieved tunable excitation–wavelength–dependent phosphorescence across different emission colors. These color-tunable foams showed outstanding phosphorescence performance, with a maximum RTP lifetime of 485.8 ms, making them promising candidates for durable, mechanically robust RTP materials. This work highlights the synergy between molecular self-assembly and hydrogen bonding in optimizing both mechanical and optical properties in natural polymer systems.

Fig. 2(II)B presents the formation of flexible molecular glasses (EG) with ultralong phosphorescent lifetimes, as reported by Nie and Yan.^133^ In this study, the authors employed an evaporation-induced self-assembly (EISA) approach using egg albumin (EA) and gelatin as biomolecular precursors. The molecular glass structure was stabilized by a combination of hydrogen bonding, electrostatic interactions, hydrophobic effects, and van der Waals forces, all of which contributed to the dense packing and aggregation of carbonyl clusters, crucial for phosphorescent emission. The resulting material achieved a maximum RTP lifetime of 180.4 ms, showcasing the effectiveness of this mild, solvent-based processing technique. The amorphous, glassy state formed through EISA allowed for reduced molecular mobility, thereby suppressing non-radiative losses and enhancing the stability of triplet states. This method demonstrates the power of bio-inspired self-assembly in crafting flexible, long-lived RTP materials without the need for heavy metals or complex processing.

#### 2.2.2. Polymeric donor and acceptor materials

The development of polymeric active materials has been central to advancing organic photovoltaic technologies, with donor–acceptor copolymers achieving power conversion efficiencies (PCEs) exceeding 18% in single-junction devices.^135^ The molecular design of these materials requires careful optimisation of multiple parameters, including energy levels, optical absorption, charge mobility, and morphological properties, to achieve optimal photovoltaic performance.

Donor polymers based on alternating donor–acceptor architectures have dominated high-efficiency organic solar cells, with materials such as PM6, D18, and PY-IT demonstrating exceptional performance when paired with appropriate acceptor materials.^136^ These polymers typically incorporate electron-rich units such as benzo[1,2-*b*:4,5-*b*′]dithiophene (BDT) as donor segments and electron-deficient units such as thieno[3,4-*c*]pyrrole-4,6-dione (TPD) or benzotriazole as acceptor segments. The alternating architecture enables fine-tuning of energy levels and optical properties through systematic modification of the donor and acceptor components.^137^

The optimization of polymer backbone planarity and intermolecular interactions has proven crucial for achieving high charge mobilities in donor polymers.^138^ Side-chain engineering strategies, employing branched alkyl chains, heteroatom substitution, and fluorination, have been successfully utilized to enhance polymer solubility while maintaining favorable solid-state packing. Advanced donor polymers have achieved hole mobilities exceeding 10^−2^ cm^2^ V^−1^ s^−1^ with optimal morphological properties for efficient charge transport.

Non-fullerene acceptor polymers have emerged as promising alternatives to small-molecule acceptors, offering advantages in terms of synthetic accessibility, batch-to-batch reproducibility, and morphological stability.^139^ These materials typically incorporate strongly electron-withdrawing units such as perylenediimide or naphthalenediimide integrated into conjugated polymer backbones. Polymer acceptors have demonstrated power conversion efficiencies (PCEs) exceeding 15% in optimized device architectures, with enhanced thermal stability compared to small-molecule systems.

The development of all-polymer solar cells utilizing both polymer donors and polymer acceptors represents a significant advancement in achieving morphological stability and mechanical flexibility.^140^ These systems have demonstrated remarkable thermal stability with minimal efficiency loss after thermal annealing at temperatures exceeding 150 °C for extended periods. The mechanical properties of all-polymer films enable exceptional flexibility, with devices maintaining their performance even after bending to radii as small as 1 mm.

Ternary blend systems incorporating multiple donor or acceptor components have shown promise for enhancing device performance through complementary absorption and optimized morphology.^141^ These systems typically employ a primary donor–acceptor pair, accompanied by a secondary component that broadens the spectral absorption or enhances charge transport properties. Optimized ternary blends have achieved (PCEs) exceeding 17% with enhanced photocurrent generation across the solar spectrum.

The incorporation of near-infrared-absorbing polymers has extended the spectral response of organic solar cells beyond 1000 nm, enabling the harvesting of previously unutilized portions of the solar spectrum.^142^ These materials typically incorporate low-bandgap units, such as diketopyrrolopyrrole, isoindigo, or thienoisoindigo, which provide strong absorption in the near-infrared region. Advanced low-bandgap polymers have maintained high open-circuit voltages despite narrow band gaps, achieving an optimal balance between photocurrent generation and voltage output.

**Fig. 3:**
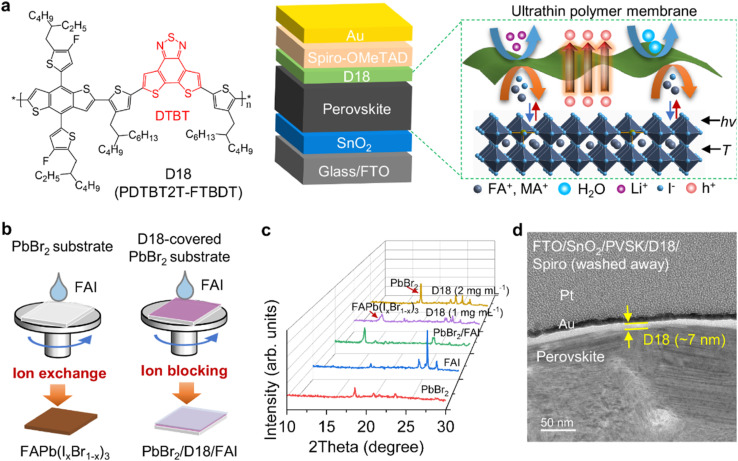
Ion-blocking effect of the D18 interlayer in perovskite solar cells. (a) Device architecture, molecular structure of D18, and schematic illustrating its ion-blocking mechanism. (b) Experimental setup for evaluating the effectiveness of D18 in blocking ion diffusion. (c) XRD analysis showing suppressed ion diffusion when FAI is spin-coated on D18-covered PbBr_2_. (d) HRTEM image of the PVSK/D18 interface, confirming conformal D18 coverage and interface integrity. Reproduced from ref. 143 with permission from Nature, (2024).

Fig. 3 illustrates the ion-blocking functionality and structural integration of the D18 polymer as a hole-selective interlayer in n-i-p perovskite solar cells (PSCs). The use of D18, a well-established donor polymer in organic photovoltaics, combines the high hole mobility and structural compactness required for both efficient charge extraction and ionic stabilization of the perovskite layer. While D18 has been the most commonly employed donor polymer and has demonstrated a notable blocking effect that improves charge separation and suppresses recombination, other polymer donors such as PM6 and PM1 have also been widely investigated. For instance, PM6 has been extensively paired with non-fullerene acceptors (*e.g.*, Y6 derivatives) to achieve (PCEs) exceeding 18–19%, while PM1 has demonstrated favorable morphology control and stability under thermal stress. In addition to polymers, small-molecule donors have also been explored, offering advantages such as well-defined molecular structures and batch-to-batch reproducibility, though they often face challenges in achieving the same film-forming properties as polymer donors.

Fig. 3a depicts the device architecture incorporating the D18 interlayer and presents the chemical structure of the D18 polymer, which features a fused-ring acceptor unit known as dithieno[3′,2′:3,4;2′′,3′′:5,6]benzo[1,2-*c*][1,2,5]thiadiazole (DTBT). This unit contributes to a planar backbone that promotes strong π–π stacking interactions, resulting in a dense and well-packed D18 layer. These properties are essential because the ultrathin D18 film, due to its conformal coverage and tight packing, acts as an effective barrier against ion migration—particularly halide ions—without sacrificing hole transport efficiency. This is critical for ensuring long-term operational stability in perovskite devices. The high fluidity of D18 in diluted solution enables uniform coverage on the perovskite surface during spin-coating, thus avoiding the typical surface discontinuities associated with thicker polymer films.

Fig. 3b provides a schematic overview of the experimental setup used to test the ion-blocking effectiveness of D18. In this context, the structure simulates the interface between perovskite and the overlying hole transport layer (HTL), where halide ions from the perovskite layer can migrate upward and degrade the device. Here, the D18 film is hypothesized to restrict this ionic movement. This schematic mirrors the function of proton exchange membranes (PEMs) in fuel cells, which conduct protons while blocking unwanted species. By analogy, D18 selectively conducts holes while inhibiting the diffusion of halide ions from the perovskite.

Fig. 3c shows X-ray diffraction (XRD) patterns to assess whether D18 effectively blocks halide ions directly. The experimental comparison involves spin-coating formamidinium iodide (FAI) onto bare PbBr_2_*versus* D18-covered PbBr_2_ films. The XRD results reveal that ion diffusion occurs readily on bare PbBr_2_, forming lead iodide phases due to reaction with FAI. In contrast, the D18-covered sample shows no evidence of such phase formation, strongly indicating that the D18 layer blocks iodide ion diffusion, validating its barrier functionality.

Fig. 3d presents a high-resolution transmission electron microscopy (HRTEM) image of the interface between the perovskite (PVSK) layer and the D18 film in the full device structure: FTO/SnO_2_/PVSK/D18. This structural analysis was performed after depositing and then removing Spiro-OMeTAD using chlorobenzene to visualize the PVSK/D18 interface without disruption. The clear, intact interface demonstrates that D18 forms a conformal and stable interlayer without delamination or mixing. Furthermore, the presence of protective Au and Pt layers (used during focused ion beam [FIB] sample preparation) ensures accurate preservation of the interface morphology for imaging.

From a materials standpoint, D18 functions as a donor polymer, specifically chosen for its exceptional hole mobility (∼3.8 × 10^−2^ cm^2^ V^−1^ s^−1^), energy level compatibility with both the perovskite and Spiro-OMeTAD, and its ability to form dense films due to temperature-dependent aggregation behavior. These features allow D18 to bridge active material functions (as a donor in organic solar cells) with interfacial engineering roles (as a hole-selective and ion-blocking interlayer in PSCs). This dual role illustrates the versatility of polymeric donors in modern hybrid optoelectronics, contributing to both performance optimization and operational stability.

#### 2.2.3. Polymeric electrodes and interconnects for flexible solar cells

Polymeric electrodes and interconnects have emerged as critical components enabling flexible, lightweight, and cost-effective solar cell technologies.^144^ The development of conductive polymers with sheet resistances below 30 Ω sq^−1^, while maintaining optical transparency exceeding 90%, has revolutionised device architectures and manufacturing processes, enabling the solution-processed fabrication of complete photovoltaic systems.

Poly(3,4-ethylenedioxythiophene) (PEDOT) based systems have dominated the transparent conductive polymer electrode landscape, with optimized formulations achieving sheet resistances as low as 20 Ω sq^−1^.^145^ The combination of high conductivity, excellent optical transparency, and mechanical flexibility makes PEDOT-based electrodes particularly attractive for flexible photovoltaic applications. Advanced processing techniques including post-deposition treatments with polar solvents and secondary doping have further enhanced the electrical properties of PEDOT films.

Hybrid electrode systems combining conductive polymers with metallic nanowires or graphene have demonstrated superior performance compared to purely polymeric systems.^146^ Silver nanowire-polymer composites have achieved sheet resistances below 10 Ω sq^−1^ with optical transparency exceeding 85%, while maintaining excellent mechanical flexibility and environmental stability. The polymer matrix provides mechanical support and environmental protection for the metallic network, while the nanowires contribute primary electrical conductivity.

The development of printable electrode inks has enabled high-throughput manufacturing of polymer-based solar cells through roll-to-roll processing at speeds exceeding 10 m min^−1^.^147^ These inks typically incorporate conductive polymers, metallic nanoparticles, and rheological modifiers to achieve optimal printing characteristics while maintaining electrical performance after deposition. Advanced ink formulations have demonstrated compatibility with various printing techniques including screen printing, inkjet printing, and gravure printing.

Interconnection strategies for polymer-based solar cells require careful consideration of thermal and mechanical stresses that can compromise long-term reliability.^148^ Flexible interconnect materials, based on conductive polymers and elastomeric substrates, have been developed to accommodate thermal expansion and mechanical deformation without compromising electrical integrity. These systems typically incorporate serpentine geometries or accordion-like structures that provide mechanical compliance while maintaining electrical continuity. The integration of self-healing properties into polymeric electrodes represents an innovative approach to enhancing device durability and reliability.^149^ These systems incorporate microcapsules containing conductive additives that are released upon mechanical damage, allowing for the autonomous repair of electrical pathways. Self-healing electrodes have demonstrated the ability to recover conductivity after multiple damage-healing cycles, potentially extending device lifetimes significantly beyond conventional systems.

Environmental stability of polymeric electrodes under outdoor conditions remains a critical consideration for commercial deployment.^150^ Advanced formulations incorporating UV stabilizers, antioxidants, and barrier coatings have demonstrated significantly improved stability compared to unprotected systems. Accelerated weathering tests have demonstrated that optimized polymeric electrodes can maintain their electrical and optical properties for periods equivalent to over 20 years of outdoor exposure.

The development of stretchable electrodes based on intrinsically conductive polymers has enabled new applications in wearable and conformable photovoltaic systems.^151^ These materials maintain electrical conductivity under mechanical strain exceeding 100%, enabling integration into textiles and curved surfaces. Advanced stretchable formulations have achieved conductivities exceeding 10^3^ S cm^−1^ while maintaining mechanical compliance and processability from solution.

### 2.3. Protective polymeric components

Protective components represent a critical frontier in polymer-enabled solar cell technologies, where multifunctional coatings serve dual purposes of enhancing optical performance while providing environmental protection. The integration of polymeric protective layers has emerged as a transformative approach to addressing the persistent challenges of surface reflectance losses, soiling degradation, and long-term UV-induced material deterioration, which significantly impact the operational efficiency and lifetime of photovoltaic systems. In the context of flexible solar modules, multifunctional protective films are especially critical. These include composite laminates with layered structures that provide mechanical robustness, gas barrier protection, and optical enhancement in a single configuration. For instance, multilayer films combining PET with sputtered or atomic layer-deposited metal oxides embedded in polymer matrices have demonstrated extremely low WVTR (<10^−5^ g m^−2^ per day) while maintaining high flexibility.^152^ The polymeric matrix in such systems absorbs mechanical stress while the inorganic nanolayers block diffusion of oxygen and moisture. Moreover, emerging self-healing polymers are being researched to repair microcracks and maintain encapsulant integrity over repeated thermal cycles and mechanical deformation.^153^ One of the primary functions of protective polymers in solar cells is to reduce surface reflectance through anti-reflective coatings (ARCs). Light reflection at the air/module interface can lead to significant energy losses, especially under diffuse lighting or at non-normal angles of incidence. To address this, polymeric materials with tailored refractive indices—such as fluorinated polymers or nano-structured coatings—are applied to the surface of solar modules. These ARCs minimize reflectance by promoting destructive interference of reflected light waves, thereby enhancing photon capture and increasing short-circuit current density (*J*_sc_).^152^ Fluorinated coatings, for instance, offer low surface energy and high transmittance, making them particularly effective as both ARCs and hydrophobic layers.^116^

Self-cleaning polymer coatings have also been widely adopted to prevent soiling, which can reduce photovoltaic output by up to 30% in dusty or humid environments. These coatings are typically made from hydrophobic or superhydrophobic polymers such as polydimethylsiloxane (PDMS), which can repel water and dust, allowing contaminants to be removed by rainfall or condensation.^154^ Some surfaces mimic natural phenomena, such as the “lotus effect,” in which micro- and nanostructures combined with low surface energy materials result in minimal adhesion of particles. This capability ensures that light transmission remains optimal over time, reducing maintenance requirements. Additionally, self-cleaning layers can protect against corrosive pollutants and salts that would otherwise compromise the stability of solar cell interfaces.

Another major degradation pathway in organic and hybrid solar cells is UV-induced photooxidation, which can lead to yellowing, cracking, and loss of functionality in sensitive polymeric and perovskite layers. To combat this, UV-absorbing polymers such as polycarbonates and poly(methyl methacrylate) (PMMA) doped with UV stabilizers, as well as cyanoacrylate-based polymers, are used as UV-blocking encapsulant layers.^155^ These materials absorb harmful UV photons while maintaining high transparency in the visible spectrum, thus extending the operational lifetime of the device. Moreover, hybrid barrier films that combine inorganic nanolayers (*e.g.*, SiO_*x*_ or Al_2_O_3_) with polymer matrices offer superior water vapor transmission rates (WVTRs) and UV resistance compared to single-material systems.^122^

#### 2.3.1. Anti-reflective coatings

Polymeric anti-reflective (AR) coatings have revolutionized solar cell surface engineering by offering solution-processable alternatives to traditional inorganic multilayer systems. Recent advances in polymer chemistry have yielded AR coatings with exceptional performance characteristics, achieving reflectance values below 2% across the solar spectrum while maintaining excellent adhesion to various substrate materials.^156,157^ These coatings typically employ gradient refractive index structures created through the controlled phase separation of polymer blends or the incorporation of nanostructured elements, such as silica nanoparticles or hollow polymer spheres. The most promising systems utilize fluorinated acrylic polymers with precisely tuned refractive indices ranging from 1.25 to 1.45, enabling broad-spectrum anti-reflective properties that can increase light transmission by 4–6% compared to untreated surfaces.^83^ Advanced formulations incorporating bio-inspired moth-eye nanostructures have demonstrated even superior performance, with some systems achieving less than 0.5% reflectance across wavelengths from 400 to 1100 nm.^158^ The scalability of these polymer-based AR coatings through roll-to-roll processing and spray coating techniques offers significant manufacturing advantages over vacuum-deposited inorganic alternatives, with production speeds exceeding 15 m min^−1^ while maintaining uniformity within a thickness variation of ±5%.^159^

#### 2.3.2. Self-cleaning surfaces

The development of self-cleaning polymeric surfaces represents a paradigm shift in solar module maintenance, addressing the critical issue of soiling losses that can reduce power output by 15–30% in dusty environments. Modern self-cleaning coatings leverage superhydrophobic and superhydrophilic mechanisms to achieve autonomous cleaning functionality through rainfall or dew formation.^160^ Superhydrophobic coatings based on fluorinated polymer matrices with hierarchical micro/nanostructures have demonstrated water contact angles exceeding 150° and sliding angles below 5°, enabling efficient removal of dust particles through the rolling of water droplets.^161^ These systems typically incorporate dual-scale roughness created through combination of polymer microspheres and nanoparticles, with the most effective formulations using perfluorinated methacrylate copolymers combined with silica nanoparticles treated with fluoroalkylsilane coupling agents.^162^ Alternatively, superhydrophilic self-cleaning surfaces utilize photocatalytic titanium dioxide nanoparticles dispersed in transparent polymer matrices to create surfaces with water contact angles below 10°, allowing uniform water spreading that carries away accumulated particles.^163^ Field testing of these self-cleaning coatings has demonstrated remarkable durability, with some systems maintaining their cleaning efficiency for over 18 months of outdoor exposure while preserving more than 95% of their initial power output, compared to 78% for uncoated reference modules.^164^ The integration of smart switching mechanisms that can alternate between superhydrophobic and superhydrophilic states depending on environmental conditions represents the next generation of adaptive self-cleaning surfaces.^165^

**Fig. 4:**
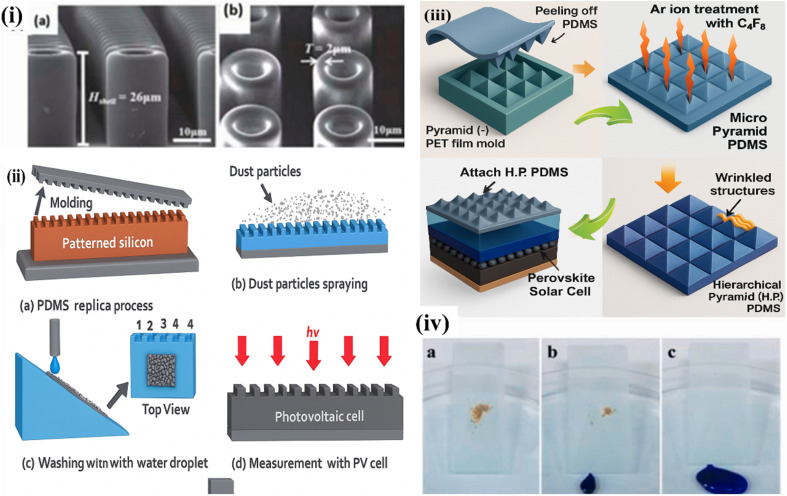
PDMS-based superhydrophobic structures for solar cell applications (i and ii). Microshell PDMS arrays and dust-removal demonstration showing superior self-cleaning. (iii and iv) Hierarchical PDMS arrays and coatings enhance perovskite solar cell efficiency and hydrophobicity. Reproduced from ref. 169 with permission from Elsevier, (2021).

Fig. 4 encapsulates the structural versatility and multifunctionality of PDMS, from microstructuring and hierarchical patterning to self-cleaning and anti-reflective performance. These innovations demonstrate how PDMS plays a critical role in developing robust, transparent, and high-efficiency superhydrophobic surfaces for photovoltaic and other surface-sensitive applications. Fig. 4 presents a comprehensive visual representation of various polydimethylsiloxane (PDMS)-based structures and their application in enhancing superhydrophobicity and photovoltaic performance, particularly in organic and perovskite solar cells. These subfigures illustrate the advancements in PDMS surface engineering, demonstrating how surface morphology and hierarchical structuring significantly contribute to water repellency and self-cleaning effects, thereby enhancing solar cell efficiency and durability.

Fig. 4(i) shows scanning electron microscopy (SEM) images of microshell PDMS arrays, with both side and top views provided. These arrays were developed by Park *et al.*^166^ and exhibit a highly ordered, dome-like microstructure that mimics natural superhydrophobic surfaces, such as those found on lotus leaves. The unique micro-topography of the PDMS shells contributes to an extremely high water contact angle (WCA) of approximately 150°, indicating excellent water-repellent properties. This structured surface was found to outperform flat PDMS in terms of self-cleaning ability due to the reduced adhesion of water droplets and dust particles, making it highly suitable for use in outdoor optical devices such as solar cells.

Fig. 4(ii) illustrates the experimental procedure for dust removal from PDMS-coated surfaces, showcasing the self-cleaning behavior of the superhydrophobic PDMS film. Dust particles deposited on the surface are easily removed by rolling water droplets, which carry the contaminants away due to minimal surface adhesion. This experiment visually demonstrates one of the key functional benefits of PDMS coatings—its ability to maintain cleanliness under environmental exposure, which is especially crucial for solar cell operation, preventing light scattering and absorption loss.

Fig. 4(iii) shows a schematic of a lotus leaf-inspired, hierarchical PDMS pyramid array fabricated for perovskite solar cells, as reported by.^167^ This structure mimics natural micro- and nano-level roughness and is laminated directly onto the surface of CH_3_NH_3_PbI_3_ perovskite solar cells. The hierarchical architecture not only provides enhanced self-cleaning properties but also offers an anti-reflective effect, leading to improved light absorption. As a result, the power conversion efficiency (PCE) of the device increased from 13.12% to 14.01%, highlighting the dual functional role of PDMS in improving both the optical and environmental performance of perovskite-based devices.

Fig. 4(iv) depicts the self-cleaning mechanism of a superhydrophobic PDMS-based coating, where water droplets roll off the surface, picking up dust and other contaminants along the way. Reproduced from,^168^ this figure effectively captures the practical utility of PDMS coatings in environmental applications. Such coatings can extend the operational lifetime of solar devices by minimizing maintenance and preserving optical clarity in real-world conditions, particularly in environments where exposure to pollutants or water is frequent.

#### 2.3.3. UV-protective polymeric layers

UV-protective polymeric layers have become indispensable components in solar cell architectures, serving as the primary defence against photodegradation while maintaining optical transparency and mechanical flexibility. Contemporary UV-protective coatings employ sophisticated approaches, including UV-absorbing chromophores, hindered amine light stabilizers (HALS), and nanoparticle-based UV filters to provide comprehensive protection across the entire UV spectrum.^170^ High-performance formulations utilize benzotriazole and benzophenone derivatives covalently bonded to polymer backbones, preventing chromophore migration and ensuring long-term stability of UV protection.^171^ These systems can absorb more than 99% of UV radiation below 380 nm while maintaining greater than 92% transmission in the visible spectrum, effectively protecting underlying layers from photodegradation without compromising photovoltaic performance.^172^ Advanced UV-protective coatings incorporating cerium oxide nanoparticles have demonstrated exceptional performance, offering UV blocking efficiency exceeding 95% while providing additional benefits, including scratch resistance and anti-static properties.^173^ The thermal stability of these protective layers has been significantly improved through the use of thermally stable polymer matrices, such as polybenzoxazole and polyimide derivatives, which enable processing temperatures of up to 220 °C while maintaining UV protection effectiveness.^174^ Accelerated aging studies have demonstrated that modules protected with advanced UV-protective coatings retain over 90% of their initial power output after 2000 hours of UV exposure, equivalent to approximately 20 years of outdoor operation, compared to 65% retention for unprotected systems.^175^ The integration of self-healing mechanisms in UV-protective coatings, utilizing microcapsule technology and thermally reversible bonds, has further enhanced the longevity and reliability of these protective systems.^176^

The synergistic integration of anti-reflective, self-cleaning, and UV-protective functionalities within single multifunctional coatings represents the current state-of-the-art in solar cell surface engineering. These advanced systems combine optical enhancement with environmental protection, offering comprehensive solutions that address multiple performance-limiting factors simultaneously while maintaining the processing advantages and cost-effectiveness that make polymer-based approaches attractive for large-scale solar deployment.^177^

## 3. Polymer substrates for flexible and lightweight solar cells

Polymer substrates are foundational to the development of next-generation flexible, lightweight, and portable solar cell technologies. Unlike rigid substrates such as glass or silicon wafers, polymer-based substrates offer mechanical flexibility, low weight, and compatibility with large-area, roll-to-roll processing methods—critical for scalable manufacturing and novel applications like wearable electronics, building-integrated photovoltaics (BIPVs), and curved or foldable surfaces. Their primary function is to provide a mechanical support platform upon which the entire photovoltaic architecture is built, while simultaneously contributing to the device's optical transparency, thermal stability, and chemical resistance.

Among the most commonly used polymer substrates in solar cell technologies are polyethylene terephthalate (PET), polyethylene naphthalate (PEN), and polyimide (PI). PET is widely employed due to its excellent optical clarity, flexibility, dimensional stability, and cost-effectiveness. It serves as a transparent support for both rigid and semi-flexible solar modules and is well-suited for low-temperature processing, typically below 150 °C.^122^ However, PET's limited thermal stability can restrict its use in devices that require high-temperature post-processing steps.

To overcome this limitation, PEN has been adopted due to its higher glass transition temperature (∼155 °C *vs.* ∼78 °C for PET), better dimensional stability, and improved resistance to moisture and UV degradation.^154^ PEN substrates are particularly beneficial in thin-film solar cell technologies, such as organic photovoltaics (OPVs) and perovskite solar cells (PSCs), where layer integrity is critical during thermal annealing. The trade-off, however, is a higher cost compared to PET, which must be considered for large-scale commercial applications.

For applications that demand superior thermal and chemical stability, polyimide (PI) has become the substrate of choice. PI films offer exceptional mechanical flexibility, thermal endurance (up to 400 °C), and resistance to solvents and radiation, making them highly suitable for space-grade photovoltaics, wearable systems, and foldable electronics.^155^ Their amber coloration, though a limitation for certain optical applications, can be tuned through chemical modifications or the use of colorless PI derivatives. Moreover, PI's excellent adhesion with active layers ensures enhanced mechanical reliability under repeated bending or rolling.

The optical properties of polymer substrates are equally important. High optical transparency in the visible range is necessary to ensure that sufficient light reaches the active layers of the device. Furthermore, surface smoothness and uniformity of polymer films influence the quality of thin-film deposition, particularly for solution-processed solar cells. Surface treatments such as plasma etching, UV-ozone exposure, or chemical modifications can be used to enhance surface energy and promote better wetting and adhesion of functional layers.^152^

Importantly, polymer substrates must also exhibit low permeability to gases such as oxygen and water vapor, which can degrade the active materials, especially in PSCs and OPVs. While the intrinsic barrier properties of PET, PEN, and PI are limited compared to glass, these can be enhanced by integrating barrier coatings, such as atomic layer-deposited (ALD) Al_2_O_3_, SiO_*x*_, or multilayer polymer/inorganic laminates, which reduce the (WVTR) to below 10^−6^ g m^−2^ per day.^122,154^

With the growing demand for eco-friendly and recyclable substrates, researchers are exploring biodegradable or bio-based polymers such as cellulose derivatives, PLA (polylactic acid), and PHA (polyhydroxyalkanoates). Although still in early stages of research, these materials hold promise for transient electronics and green energy systems, where device disposability and environmental compatibility are critical.^118^

In conclusion, polymer substrates are not merely structural supports but active enablers of flexibility, processability, and functionality in modern solar cell devices. The tailored selection of polymer type, based on application-specific needs such as thermal processing conditions, mechanical requirements, optical clarity, and environmental resistance, allows for the integration of photovoltaic systems into diverse environments and form factors. As the field advances, continued research into multifunctional, recyclable, and high-performance polymers will be crucial to overcoming the current limitations and expanding the applicability of solar energy technologies beyond conventional installations.

### 3.1. Material requirements and challenges

The development of polymer substrates for flexible solar cells represents one of the most challenging aspects of photovoltaic technology advancement, requiring the simultaneous optimization of multiple conflicting material properties to achieve commercially viable performance. The unique combination of thermal stability, optical transparency, barrier properties, and mechanical flexibility demands sophisticated polymer engineering approaches that push the boundaries of traditional material science while maintaining cost-effectiveness and manufacturing scalability.

#### 3.1.1. Thermal stability requirements (>150 °C processing)

Thermal stability emerges as perhaps the most critical constraint in polymer substrate development, as modern solar cell manufacturing processes require sustained exposure to elevated temperatures during deposition, annealing, and encapsulation steps. The requirement for processing temperatures exceeding 150 °C poses significant challenges for conventional polymer systems, which often exhibit glass transition temperatures, melting points, or degradation onset temperatures below these critical thresholds.^178^ Advanced polymer substrates must maintain dimensional stability, optical clarity, and mechanical integrity throughout thermal cycling while avoiding outgassing, thermal degradation, or property drift that could compromise device performance.^179^ High-performance thermoplastic substrates based on polyethylene naphthalate (PEN) and polyethylene terephthalate (PET) derivatives have demonstrated excellent thermal stability up to 180 °C, with some specially formulated grades withstanding continuous exposure at 200 °C for periods exceeding 1000 hours without significant property degradation.^180^ Engineering thermoplastics, such as polyimide (PI) and polybenzoxazole (PBO), offer superior thermal performance, with service temperatures extending to 250 °C and beyond, although often at the expense of optical transparency and processing complexity.^181^ The development of thermally stable polymer substrates requires careful consideration of polymer backbone chemistry, with aromatic structures generally providing enhanced thermal stability compared to aliphatic systems. In contrast, the incorporation of heat-resistant additives such as phenolic antioxidants and phosphorus-based thermal stabilisers can further extend usable temperature ranges.^182^ Cross-linked polymer systems offer an alternative approach to enhanced thermal stability, with thermoset substrates based on benzocyclobutene (BCB) and polyimide precursors demonstrating exceptional thermal performance; however, this typically requires more complex processing protocols and may compromise mechanical flexibility.^183^

#### 3.1.2. Optical transparency requirements (>85% transmission)

Achieving high optical transparency across the solar spectrum represents a fundamental challenge in polymer substrate design, as the competing requirements for thermal stability and mechanical robustness often necessitate polymer structures and additives that can compromise optical performance. The target of greater than 85% transmission across wavelengths from 400 to 1100 nm requires careful optimization of the polymer molecular structure to minimize chromophoric groups while maintaining necessary thermal and mechanical properties.^182^ Absorption losses in polymer substrates typically arise from several sources, including aromatic conjugation, carbonyl groups, residual catalysts, and additive systems, with each contributing to specific wavelength-dependent absorption features that must be systematically addressed through molecular design.^184^ Advanced polymer substrates utilizing aliphatic polyester backbones with carefully selected co-monomer compositions have achieved transmission values exceeding 90% across the visible spectrum while maintaining adequate thermal stability for processing temperatures up to 160 °C.^185^ The incorporation of UV-absorbing additives to enhance long-term stability often presents trade-offs with optical transparency, requiring the development of selective UV filters that block harmful radiation below 380 nm while maintaining high transmission in the photovoltaically active region.^186^ Fluorinated polymer systems offer exceptional optical clarity due to their low polarizability and reduced light scattering, with some fluorinated substrates achieving transmission values above 95%, although this often comes at significantly higher material costs and processing complexity.^178^ Anti-reflective treatments applied to polymer substrate surfaces can enhance effective transmission by 3–5% through reduction of Fresnel reflection losses, with some systems incorporating gradient refractive index structures to achieve broadband anti-reflective performance.^83^ The challenge of maintaining optical transparency during thermal processing requires understanding of thermally induced optical changes, including polymer chain relaxation, additive migration, and potential haze formation, which can significantly impact long-term optical performance.^187^

#### 3.1.3. Barrier properties requirements (WVTR <10^−6^ g m^−2^ per day)

The stringent barrier property requirements for solar cell substrates necessitate (WVTRs) below 10^−6^ g m^−2^ per day to prevent moisture-induced degradation of photovoltaic components, representing one of the most challenging specifications in flexible electronics packaging. Achieving such low permeability levels requires sophisticated barrier coating systems or specialized polymer formulations that can effectively block molecular transport while maintaining flexibility and optical transparency.^188^ Conventional polymer substrates typically exhibit WVTR values several orders of magnitude higher than required specifications, with standard PET and PEN substrates showing transmission rates in the range of 10^−2^ to 10^−3^ g m^−2^ per day, necessitating the application of advanced barrier enhancement technologies.^189^ Multilayer barrier systems incorporating alternating organic and inorganic layers have demonstrated exceptional performance, with some configurations achieving WVTR values below 10^−7^ g m^−2^ per day through the creation of tortuous diffusion pathways that dramatically reduce molecular transport rates.^190^ Atomic layer deposition (ALD) of ultra-thin oxide layers on polymer substrates has emerged as a leading approach for achieving ultra-low permeability, with alumina and silica layers as thin as 20–50 nm providing barrier enhancement factors exceeding 10^4^ when properly applied.^191^ The mechanical flexibility of barrier-enhanced substrates presents significant challenges, as inorganic barrier layers are inherently brittle and prone to cracking under mechanical stress, requiring the development of flexible barrier concepts including segmented structures and self-healing mechanisms.^192^ Polymer-based barrier systems utilizing liquid crystalline polymers and highly oriented structures have shown promise for achieving low permeability while maintaining flexibility, with some systems demonstrating WVTR values approaching 10^−5^ g m^−2^ per day through molecular engineering approaches.^193^ The integration of moisture-scavenging systems within polymer substrates represents an additional approach to managing water vapor ingress, with reactive desiccants and molecular sieves providing dynamic moisture control that can compensate for finite barrier performance.^194^

#### 3.1.4. Mechanical flexibility requirements (>10^4^ bend cycles)

The mechanical flexibility requirements for polymer substrates in flexible solar cells demand exceptional fatigue resistance, with the ability to withstand more than 10 000 bend cycles without catastrophic failure or significant property degradation. This requirement encompasses not only the substrate material itself but also its interaction with deposited layers, adhesives, and encapsulation systems, which collectively determine the overall device flexibility.^179^ The fundamental challenge lies in maintaining electrical conductivity and optical transparency of deposited layers while accommodating the mechanical strain associated with bending, twisting, and stretching that flexible solar cells must endure in practical applications.^178^ Modern polymer substrates achieve exceptional flexibility through careful control of molecular weight, chain architecture, and processing conditions, with some systems demonstrating bend cycle performance exceeding 10^5^ cycles at bend radii as small as 2 mm.^183^ The mechanical behavior of polymer substrates under cyclic loading involves complex phenomena, including stress relaxation, creep, and fatigue crack propagation, which require a comprehensive understanding of polymer physics and failure mechanisms to optimize long-term performance.^182^ Thermoplastic substrates based on polyimide and PEN have demonstrated superior flexibility compared to thermoset systems, with the ability to accommodate large strains without brittle failure while maintaining dimensional stability under thermal cycling conditions.^181^ The interaction between substrate flexibility and deposited functional layers represents a critical design consideration, as the mechanical properties of multilayer systems can be dominated by the stiffest component, potentially negating the benefits of flexible substrates if electrode or barrier layers are not properly designed for mechanical compatibility.^182^ Advanced characterization techniques including dynamic mechanical analysis, fatigue testing, and *in situ* monitoring of electrical properties during mechanical cycling have become essential tools for evaluating substrate performance and optimizing material selection for specific applications.^184^

The simultaneous achievement of all four critical requirements represents the ultimate challenge in polymer substrate development, requiring holistic material design approaches that consider the complex interactions between thermal, optical, barrier, and mechanical properties. The most successful substrate systems employ multi-component approaches combining high-performance polymer matrices with specialized additives, surface treatments, and barrier coatings to achieve the multifunctional performance required for commercial flexible solar cell applications.^185^ Future developments in polymer substrate technology focus on bio-based alternatives, recyclable systems, and smart materials that can adapt their properties in response to environmental conditions, positioning flexible solar cells as truly sustainable energy solutions.^178^

**Fig. 5:**
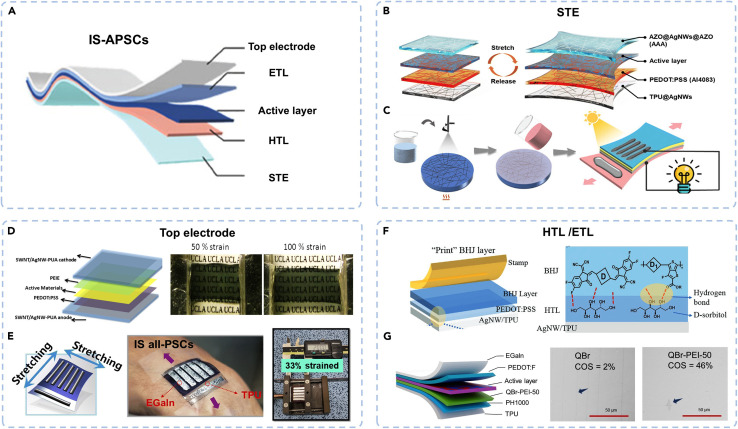
Stretchable architectures and key functional layers of IS-APSCs. (A) Schematic structure of IS-APSCs with stretchable components. (B and C) AgNW-based bottom electrodes embedded in TPU for high conductivity and elasticity. (D and E) Stretchable top electrodes using SWNT/AgNW networks and EGaIn liquid metal. (F and G) Enhanced HTL and ETL layers using optimized PEDOT:PSS and quinoidal polymer for improved charge transport and stretchability. Reproduced from ref. 200 with permission from Elsevier, (2025).

Fig. 5A presents the core architecture of an intrinsically stretchable all-polymer solar cell (IS-APSC), highlighting the functional layers essential for maintaining high photovoltaic performance under mechanical deformation. Central to this design is the stretchable transparent electrode (STE), whose surface is modified using PEDOT:PSS to reduce surface roughness. This modification not only improves the physical interface with adjacent layers but also enhances charge collection efficiency and overall power conversion efficiency (PCE). The schematic highlights the requirement for highly stretchable substrates with smooth surfaces to facilitate stable energy generation under repeated strain.

Fig. 5B illustrates the implementation of a silver nanowire (AgNW)-based bottom electrode layer, specifically a composite of AgNWs embedded within thermoplastic polyurethane (TPU). Developed by Li *et al.*,^195^ this structure offers a combination of high optical transparency, mechanical flexibility, and excellent tensile durability. The electrode's compatibility with solution-processing techniques also contributes to scalable and low-cost fabrication, resulting in a record PCE of 9.52% for IS-APSCs fabricated *via* this method.

Fig. 5C shows a more advanced embedding strategy where the AgNW conductive network is fully integrated into the TPU elastomer matrix. This design, proposed by Han *et al.*,^196^ leverages the viscoelastic properties of the polymer matrix to distribute mechanical stress, enabling the electrode to maintain both conductivity and structural integrity under tensile strains exceeding 50%. The resulting IS-APSC achieved a PCE of 12.5%, demonstrating the effectiveness of embedding conductive networks in soft polymer matrices.

Fig. 5D focuses on a hybrid top electrode composed of single-walled carbon nanotubes (SWNTs) and AgNWs dispersed in a polyurethane acrylate matrix. Reported by Valluvar *et al.*,^200^ this composite electrode supports high optical transmittance and can tolerate tensile strains of up to 100%, making it well-suited for stretchable photovoltaic devices. Its percolating network structure enables efficient charge transport, while the flexible matrix ensures mechanical resilience.

Fig. 5E depicts the application of eutectic gallium–indium (EGaIn) as a top electrode. Liquid metals, such as EGaIn, offer unique advantages, including conformal contact, high conductivity, and mechanical deformability. Lee *et al.*^197^ demonstrated the use of drop-cast EGaIn for patterned, stretchable electrodes, greatly improving both the mechanical stretchability and electrical performance of IS-APSCs. However, challenges such as oxidation and environmental stability must be addressed for practical deployment.

Fig. 5F illustrates the optimization of PEDOT:PSS for use as a highly stretchable hole-transport layer (HTL). By treating PEDOT:PSS films with strong acids and incorporating additives like D-sorbitol and polyethylene glycol, Wang *et al.*^198^ achieved enhanced conductivity, reduced phase separation, and improved tensile properties. These modifications enabled the formation of nanofiber-like structures within the HTL, contributing to both high charge mobility and mechanical robustness.

Fig. 5G introduces a cross-linkable quinoidal compound developed by Wang *et al.*^199^ as a novel stretchable electron transport layer (ETL). This polymeric ETL exhibits a high electrical conductivity of 0.049 S m^−1^ and a crack onset strain above 45%, making it well-suited for integration into deformable solar cells. The cross-linking strategy provides excellent film-forming capability and mechanical durability, which are vital for minimizing degradation under cyclic mechanical stress.

### 3.2. Surface treatments and modifications

Surface treatments and modifications of polymer substrates are crucial for achieving the mechanical integrity, environmental durability, and electronic performance necessary for high-efficiency flexible solar cell applications. Pristine polymer surfaces—such as those of PET, PEN, or PI—typically exhibit low surface energy, weak chemical reactivity, and poor wettability, which hinders the uniform deposition and strong adhesion of subsequent functional layers, including electrodes, active layers, or encapsulants. Moreover, their inherent barrier properties to moisture and oxygen are insufficient for long-term outdoor use in photovoltaic (PV) devices. Therefore, surface engineering strategies are crucial to tailoring interfacial interactions and enhancing the compatibility of polymers with device fabrication processes and operating environments.^179^

Modern approaches to surface modification employ physical, chemical, and hybrid techniques, often used in tandem to precisely adjust surface energy, functional group density, and topography without altering the bulk mechanical or optical properties of the polymer substrate. Among physical techniques, plasma treatment (*e.g.*, oxygen, argon, or ammonia plasma) is one of the most effective and widely used. It introduces polar functional groups (*e.g.*, hydroxyl, carboxyl, or amine groups) onto the polymer surface, significantly increasing surface energy and promoting better wetting and adhesion of solution-processed layers.^122^ Plasma treatment can also induce nano-scale roughness that mechanically interlocks deposited films, further enhancing film adhesion and structural robustness.

UV-ozone treatment is another common surface activation technique that uses high-energy ultraviolet radiation to generate reactive oxygen species, which oxidize the polymer surface, increase hydrophilicity, and introduce chemically reactive groups. This method is especially suitable for transparent substrates, such as PET or PEN, as it minimally alters optical transmission while improving interfacial compatibility with conductive polymers or metal electrodes.^154^ However, care must be taken to avoid overexposure, which may lead to surface degradation or yellowing of the substrate.

Chemical functionalization techniques involve grafting or coating the substrate surface with reactive molecules or polymer brushes that improve adhesion and compatibility. For instance, silanization with organosilanes, such as aminopropyltriethoxysilane (APTES), can form stable covalent bonds with hydroxylated polymer surfaces (*e.g.*, post-plasma-treated PI or PET), providing anchor points for the subsequent attachment of metal nanoparticles, conductive polymers, or active layers.^116^ This approach is particularly valuable for integrating biofunctionalized surfaces, transparent electrodes, or nanostructured charge transport layers.

To further enhance the barrier performance, multilayer hybrid coatings that combine inorganic and organic materials are utilized. Atomic layer deposition (ALD) or chemical vapor deposition (CVD) techniques can deposit ultra-thin inorganic oxide layers (*e.g.*, Al_2_O_3_, SiO_*x*_) that dramatically reduce the (WVTR), while maintaining flexibility when integrated with polymeric interlayers.^152^ These hybrid coatings not only prevent ingress of oxygen and moisture—key degradation pathways for organic and perovskite solar cells—but also preserve the mechanical properties and bending durability of the flexible substrate.

Emerging strategies include laser patterning, electrochemical activation, and layer-by-layer (LbL) assembly to create gradient or patterned surfaces with spatially controlled properties. Such techniques can direct the crystallization of donor or acceptor materials in the active layer or enable the selective deposition of electrodes in printed electronics.^118^ In advanced applications, self-assembled monolayers (SAMs) are utilized to fine-tune the work function of surfaces and tailor the electronic band alignment at interfaces, thereby further optimizing charge transport and extraction in the device stack.

#### 3.2.1. Plasma treatment for adhesion enhancement

Plasma treatment has emerged as the most widely adopted surface modification technique for enhancing adhesion between polymer substrates and subsequently deposited layers, offering precise control over surface chemistry and morphology through carefully controlled ionized gas environments. The fundamental mechanism of plasma treatment involves the generation of reactive species, ions, and electrons that interact with polymer surfaces to create new chemical functionalities, increase surface energy, and develop micro-roughness that enhances mechanical interlocking with deposited materials.^183^ Oxygen plasma treatment represents the most common approach, creating hydroxyl, carbonyl, and carboxyl functional groups on polymer surfaces that dramatically improve wettability and chemical bonding with overlying layers, with contact angle reductions from >90° to <20° commonly achieved within treatment times of 30–120 seconds.^180^ The effectiveness of plasma treatment depends critically on processing parameters including gas composition, power density, treatment time, and chamber pressure, with optimal conditions varying significantly between different polymer substrate materials and intended applications.^178^ Advanced plasma treatment protocols utilizing argon, nitrogen, or mixed gas compositions can create specific surface chemistries tailored to particular adhesion requirements. Notably, nitrogen plasma generates amine functionalities that enhance bonding with metal oxides, while argon plasma creates activated surfaces without introducing heteroatoms.^182^ The temporal stability of plasma-treated surfaces is a significant practical consideration, as surface energy and functional group density typically decrease over time due to polymer chain relaxation and atmospheric contamination, necessitating careful control of processing sequences and storage conditions to maintain treatment effectiveness.^181^ Atmospheric pressure plasma treatments have gained increasing attention for industrial applications, offering continuous processing capabilities and eliminating the need for vacuum systems while achieving comparable surface modification effects to low-pressure treatments.^182^ The integration of plasma treatment with other surface modification techniques, including chemical priming and silane coupling agents, can provide synergistic adhesion enhancement effects that exceed the performance of individual treatments alone.^184^

#### 3.2.2. Barrier coatings: SiO_*x*_ and Al_2_O_3_ systems

Inorganic barrier coatings based on silicon oxide (SiO_*x*_) and aluminum oxide (Al_2_O_3_) represent the current state-of-the-art for achieving ultra-low (WVTR) on polymer substrates. With properly engineered systems, permeability can be reduced by factors exceeding 10^4^ compared to uncoated substrates. Silicon oxide barrier layers, typically deposited through plasma-enhanced chemical vapor deposition (PECVD) or atomic layer deposition (ALD), can achieve water vapor transmission rates (WVTRs) below 10^−5^ g m^−2^ per day when applied as continuous films with thicknesses ranging from 50 to 200 nm.^185^ The barrier performance of SiO_*x*_ coatings depends critically on film stoichiometry, with silicon-rich compositions (*x* < 2) generally providing superior barrier properties compared to stoichiometric SiO_2_ due to increased density and reduced porosity, though often at the expense of optical transparency.^188^ Aluminum oxide barrier layers deposited *via* ALD have demonstrated exceptional performance, with some systems achieving WVTR values below 10^−6^ g m^−2^ per day through the formation of highly conformal, pinhole-free coatings that effectively seal substrate surface defects.^186^ The mechanical flexibility of inorganic barrier coatings on polymer substrates presents significant engineering challenges, as the inherent brittleness of ceramic materials leads to crack formation under mechanical stress, potentially compromising barrier integrity and creating pathways for moisture ingress.^178^ Advanced barrier coating strategies employ segmented or multilayer architectures that can accommodate substrate deformation while maintaining barrier performance. Some systems incorporate stress-relief interlayers or engineered crack patterns to prevent catastrophic failure during bending.^187^ The thermal stability of barrier coatings during solar cell processing necessitates careful consideration of the thermal expansion mismatch between inorganic layers and polymer substrates, as differential expansion can lead to coating delamination or stress-induced cracking at elevated temperatures.^83^ Hybrid barrier systems combining SiO_*x*_ and Al_2_O_3_ layers have shown promise for achieving optimal barrier performance while minimizing stress-related failures, with alternating layer structures providing redundant barrier pathways and improved mechanical compliance.^189^ The integration of barrier coatings with plasma treatment represents a synergistic approach that enhances both adhesion and barrier performance, with plasma activation of polymer surfaces prior to coating deposition significantly improving interfacial bonding and reducing coating defects.^190^

#### 3.2.3. Hybrid organic–inorganic systems

Hybrid organic–inorganic surface treatment systems represent an advanced approach that combines the processing advantages and flexibility of organic materials with the superior barrier and thermal properties of inorganic components, creating multifunctional interfaces that address multiple performance requirements simultaneously. These systems typically employ sol–gel chemistry, molecular self-assembly, or reactive coupling strategies to create covalently bonded organic–inorganic networks that exhibit properties superior to either component alone.^193^ Silane-based coupling agents represent the most widely used hybrid approach, providing molecular bridges between polymer substrates and inorganic overlayers through bifunctional molecules that contain both organic-reactive and inorganic-reactive groups.^191^ Advanced hybrid systems utilizing organically modified silicates (ORMOSILs) have demonstrated exceptional performance in combining barrier properties with mechanical flexibility, achieving WVTR values below 10^−4^ g m^−2^ per day while maintaining bendability over 10^4^ cycles through the incorporation of flexible organic segments within the inorganic network.^192^ The development of hybrid barrier systems through layer-by-layer assembly techniques enables precise control over coating thickness, composition, and functionality. Some systems incorporate multiple hybrid layers with different organic–inorganic ratios to create gradient structures that optimize both barrier and mechanical properties.^194^ Sol–gel-derived hybrid coatings offer exceptional versatility in tailoring surface properties, with the ability to incorporate functional additives, including UV absorbers, antioxidants, and adhesion promoters, directly into the hybrid matrix, creating multifunctional surface treatments that address multiple performance requirements in a single processing step.^179^ The thermal stability of hybrid organic–inorganic systems generally exceeds that of purely organic treatments, while maintaining superior flexibility compared to purely inorganic coatings. With properly designed systems, stable performance is demonstrated at temperatures up to 200 °C for extended periods.^183^ Nanoparticle-reinforced hybrid systems incorporating silica, titania, or alumina nanoparticles within organic matrices have demonstrated remarkable property enhancements, with barrier performance improvements exceeding 100-fold compared to unfilled organic coatings, while maintaining optical transparency above 90%.^178^ The processing compatibility of hybrid systems with roll-to-roll manufacturing represents a significant advantage for large-scale production, with solution-processable formulations enabling continuous coating application at line speeds exceeding 10 m min^−1^ while maintaining coating uniformity and performance.^182^ Advanced hybrid architectures incorporating self-healing mechanisms through reversible bonds or encapsulated healing agents have demonstrated the ability to recover barrier performance after mechanical damage, representing a significant advancement in long-term reliability for flexible solar cell applications.^185^

The integration of plasma treatment, barrier coatings, and hybrid organic–inorganic systems represents the current frontier in polymer substrate surface engineering, with optimized treatment sequences capable of achieving the simultaneous requirements of excellent adhesion, ultra-low permeability, and mechanical flexibility that define high-performance flexible solar cell substrates. The continued development of these surface treatment technologies focuses on reducing processing complexity, improving environmental compatibility, and enhancing long-term stability under realistic operating conditions, positioning treated polymer substrates as enabling components for next-generation flexible photovoltaic systems.^181^

## 4. Encapsulation polymers – protecting solar cells

Encapsulation polymers are indispensable components in solar cell modules, acting as protective barriers that shield sensitive photovoltaic materials from mechanical damage, environmental degradation, and photothermal stresses. Among these materials, ethylene vinyl acetate (EVA) has emerged as the most widely used encapsulation polymer, particularly in silicon-based and thin-film solar modules, due to its excellent transparency, strong adhesion to glass and other module components, and reliable processing characteristics. EVA encapsulants serve dual roles: they mechanically secure the layers of a solar module and optically enhance device efficiency by maintaining high light transmittance while suppressing reflectance and scattering. Structurally, EVA is a copolymer composed of ethylene and vinyl acetate units. The vinyl acetate content, typically ranging between 28% and 33%, gives EVA its flexibility, adhesive strength, and resistance to cracking or embrittlement under thermal cycling.^154^ Upon thermal lamination—generally at temperatures around 140–150 °C—EVA undergoes crosslinking (initiated by peroxides), transforming into a durable thermoset that securely bonds the solar cell layers. This crosslinked structure enhances mechanical stability and dimensional retention while preventing delamination during outdoor exposure.

EVA's optical clarity is one of its most essential attributes. With a refractive index (∼1.48) that matches well with glass and silicon, EVA minimizes Fresnel losses and enhances light transmission into the active layer. It also acts as a physical cushion, absorbing mechanical shocks or stresses caused by hail, wind loads, or thermal expansion mismatches between different module layers. This makes EVA an essential material for both rigid and flexible module architectures.^122^

However, EVA is not without limitations. Its susceptibility to UV degradation is a major concern for long-term outdoor operation. Under prolonged exposure to UV light, EVA can undergo yellowing due to the formation of chromophoric degradation products, including acetic acid and carbonyl groups.^155^ This yellowing not only reduces optical transmission, thereby lowering device efficiency, but the acetic acid can corrode metallic contacts and degrade encapsulated layers. To mitigate this, EVA formulations are commonly stabilized with UV absorbers, antioxidants, and metal deactivators that extend their operational lifetime and delay degradation pathways.

EVA also exhibits moderate barrier properties to oxygen and moisture. While it provides some resistance, it is often used in conjunction with front glass and rear barrier films (such as fluoropolymer backsheets) to meet the strict IEC 61215 and IEC 61730 standards for module reliability. In more advanced module designs, especially those employing organic photovoltaics (OPVs) or perovskite solar cells (PSCs), where moisture sensitivity is critical, EVA may be combined with multi-layer barrier stacks (*e.g.*, inorganic/organic hybrids) to achieve ultra-low (WVTR < 10^−6^ g m^−2^ per day).^152^

From a manufacturing perspective, EVA is attractive because of its low cost, scalable processing, and excellent adhesion to glass, silicon, TCOs (transparent conductive oxides), and backsheet films. Its broad adoption in the solar industry has also led to standardization of lamination processes, enabling high-throughput fabrication of reliable modules using roll-to-roll or batch lamination techniques. Additionally, transparent EVA variants doped with light-diffusing or luminescent particles are being developed to further enhance photon management in the device stack, thereby improving light-harvesting efficiency and thermal stability.^116^

Despite emerging alternatives such as thermoplastic polyolefins (TPOs), polyvinyl butyral (PVB), and ionomers, EVA remains the benchmark encapsulant in commercial photovoltaic manufacturing, primarily due to its well-balanced properties and cost-efficiency. Continuous innovation in EVA formulation, including improved crosslinking kinetics, enhanced photostabilizers, and hybrid encapsulants, is being pursued to address its limitations and tailor it to next-generation solar technologies, such as bifacial, ultra-thin, and flexible solar cells.

**Fig. 6:**
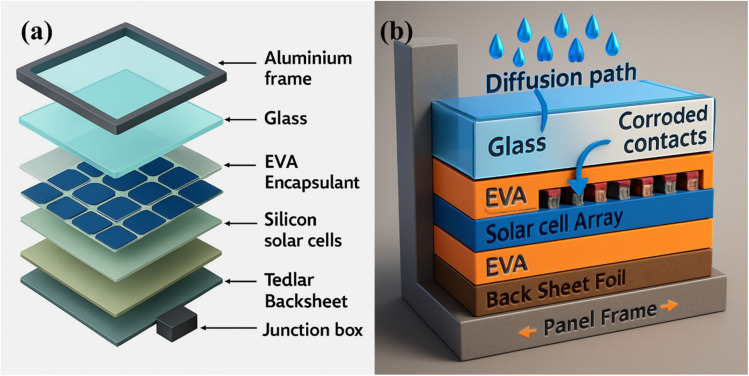
(a) Exploded view of a crystalline silicon photovoltaic module showing the layered structure from the aluminum frame and protective glass to the silicon solar cells, EVA encapsulant, Tedlar backsheet, and junction box. (b) Cross-sectional illustration of moisture-induced degradation pathway in solar panels, demonstrating how water infiltration leads to corrosion of silver contacts and reduced module efficiency. Reproduced from ref. 201 with permission from Elsevier, (2020).

Fig. 6a shows the layered construction of a crystalline silicon (c-Si) photovoltaic solar panel module. The diagram presents an exploded view of all the essential components stacked from top to bottom. At the top is the aluminum frame that provides structural support and mounting capability. Below that is the protective glass layer that allows sunlight to pass through while shielding the internal components from environmental damage. The EVA (Ethylene Vinyl Acetate) encapsulant layer is next, serving as a transparent adhesive that bonds and protects the solar cells. The heart of the module is the silicon solar cells layer, where the actual photovoltaic conversion occurs. Another EVA encapsulant layer follows to provide additional protection and bonding. At the bottom is the Tedlar backsheet, which serves as a weather-resistant barrier to protect the rear of the module. Finally, the junction box is attached to facilitate electrical connections and house the bypass diodes.

Fig. 6b demonstrates the degradation pathway that affects solar panel performance over time. This cross-sectional view shows how moisture infiltration leads to module deterioration. Water droplets penetrate through the diffusion path in the glass layer, eventually reaching the solar cell array. The diagram illustrates how this moisture causes corrosion of the electrical contacts (fingers) on the solar cells, which are typically made of silver. The EVA encapsulant layers are shown sandwiching the solar cell array, with the back sheet foil and panel frame providing structural support. This moisture-induced corrosion of the silver fingers increases the series resistance of the solar cells, ultimately reducing the module's electrical efficiency and overall performance. The one-dimensional simulation approach mentioned in the text is justified because the layer thickness is minimal compared to the module's overall area, making this simplified model appropriate for studying the degradation process.

### 4.1. EVA (ethylene vinyl acetate) systems

Ethylene vinyl acetate (EVA) encapsulation systems have dominated the photovoltaic industry for over four decades, establishing themselves as the benchmark technology for solar cell protection through their unique combination of optical transparency, mechanical flexibility, and cost-effectiveness. The widespread adoption of EVA systems is driven by their ability to provide reliable long-term protection while maintaining excellent optical coupling between solar cells and the external environment, achieved through the careful optimization of polymer composition, crosslinking chemistry, and stabilization strategies.^179^ Modern EVA formulations represent sophisticated polymer engineering achievements, incorporating advanced crosslinking agents, UV stabilizers, and processing aids that enable reliable operation under diverse environmental conditions while maintaining the economic advantages that have made EVA the industry standard for photovoltaic encapsulation applications.

#### 4.1.1. Crosslinking mechanisms in EVA systems

The crosslinking behavior of EVA encapsulation systems represents a critical aspect of their performance, fundamentally determining the mechanical properties, thermal stability, and long-term durability of photovoltaic modules through the formation of three-dimensional polymer networks that prevent flow and maintain dimensional stability under operational stresses. EVA crosslinking typically occurs through free radical mechanisms initiated by organic peroxides, with 2,5-dimethyl-2,5-di(*tert*-butylperoxy)hexane (DBPH) serving as the most widely used crosslinking agent due to its optimal decomposition temperature profile and compatibility with lamination processing conditions.^178^ The crosslinking reaction proceeds through a complex series of steps involving peroxide decomposition, hydrogen abstraction from EVA chains, and subsequent radical coupling reactions that create carbon–carbon bonds between polymer chains, with the overall process strongly dependent on temperature, time, and peroxide concentration.^180^ Optimal crosslinking typically requires temperatures between 140 °C and 160 °C for durations of 10–20 minutes, with the degree of crosslinking monitored through gel content measurements that indicate the fraction of polymer chains incorporated into the crosslinked network.^183^ The vinyl acetate (VA) content of EVA copolymers significantly influences crosslinking behavior, with higher VA content generally facilitating crosslinking through increased chain mobility and reduced crystallinity. However, excessive VA levels can compromise optical properties and thermal stability.^182^ Advanced EVA formulations incorporate crosslinking enhancers, such as triallyl isocyanurate (TAIC) or zinc diacrylate, which increase crosslinking efficiency and provide additional crosslinking pathways, enabling more complete network formation and improved mechanical properties.^182^ The crosslinking process must be carefully controlled to avoid overcrosslinking, which can lead to brittleness and stress concentration, or undercrosslinking, which results in insufficient dimensional stability and potential delamination under thermal cycling conditions.^181^ Modern crosslinking strategies employ multi-stage curing profiles that optimize both crosslinking efficiency and stress relief, with some systems incorporating post-curing treatments that enhance network completion and stress relaxation.^184^ The interaction between crosslinking chemistry and other EVA components, particularly UV stabilizers and processing aids, requires careful formulation optimization to prevent interference with crosslinking reactions while maintaining overall system performance.^185^

#### 4.1.2. UV stabilization strategies for EVA systems

UV stabilization represents perhaps the most critical aspect of EVA formulation for photovoltaic applications, as prolonged exposure to solar radiation can cause polymer degradation, yellowing, and loss of mechanical properties that compromise module performance and lifetime. The challenge of UV stabilization in EVA systems is compounded by the requirement for excellent optical transparency, which limits the use of UV-absorbing additives that might interfere with photovoltaic performance.^188^ Modern EVA stabilization strategies employ multi-component systems that combine UV absorbers, hindered amine light stabilizers (HALS), and antioxidants, providing synergistic protection against photodegradation while maintaining optical clarity and processing compatibility.^178^ Benzotriazole-based UV absorbers represent the most widely used stabilizer class, offering broad-spectrum UV protection through efficient energy dissipation mechanisms that convert absorbed UV energy into harmless heat. 2-(2-Hydroxy-5-methylphenyl)benzotriazole (Tinuvin P) serves as a benchmark stabilizer for EVA applications.^187^ The effectiveness of UV stabilization depends critically on the stabilizer concentration, distribution, and thermal stability during processing, with typical loading levels ranging from 0.1% to 0.5% by weight to achieve optimal protection without compromising optical properties.^83^ Hindered amine light stabilizers (HALS) provide complementary protection through radical scavenging mechanisms that interrupt photodegradation chain reactions, with some HALS systems offering regenerative stabilization, which maintains effectiveness over extended exposure periods.^186^ The development of thermally stable UV stabilizers represents a key advancement in EVA technology, with high-performance stabilizers maintaining effectiveness at lamination temperatures exceeding 150 °C while providing long-term stability under operational conditions.^189^ Advanced stabilization systems incorporate multiple UV absorbers with different spectral characteristics to provide broad-spectrum protection. Some formulations utilize combinations of benzotriazole and benzophenone derivatives to achieve comprehensive UV coverage.^190^ The interaction between UV stabilizers and crosslinking chemistry requires careful optimization to prevent stabilizer interference with peroxide decomposition or crosslinking reactions. Some systems employ delayed-action stabilizers that become active only after crosslinking is complete.^191^ Innovative stabilization approaches include the use of UV-screening layers or gradient stabilizer distributions that provide maximum protection at the air-facing surface while minimizing interference with optical transmission in the bulk material.^192^

#### 4.1.3. Performance in different climate conditions

The performance of EVA encapsulation systems under diverse climatic conditions represents a critical consideration for global photovoltaic deployment, as environmental factors, including temperature, humidity, UV irradiance, and atmospheric pollutants, can significantly impact polymer degradation rates and module reliability. Desert climates present particular challenges for EVA systems due to extreme temperature cycling, intense UV radiation, and potential sand abrasion. Some desert installations experience module temperatures exceeding 85 °C and UV doses several times higher than those in moderate climates.^193^ Thermal degradation mechanisms, including deacetylation, crosslinking degradation, and stabilizer consumption govern the high-temperature performance of EVA systems. With proper formulation and processing, reliable operation at elevated temperatures can be achieved for extended periods.^194^ Humid tropical climates present distinct challenges through accelerated hydrolysis reactions, particularly at elevated temperatures, where water vapor ingress can lead to EVA degradation, corrosion of electrical components, and delamination at material interfaces.^179^ The development of humidity-resistant EVA formulations incorporates moisture scavengers, corrosion inhibitors, and enhanced barrier properties that minimize water uptake and mitigate hydrolysis reactions, enabling reliable operation in high-humidity environments.^178^ Cold climate performance requires consideration of low-temperature flexibility, thermal shock resistance, and potential ice loading, with EVA systems maintaining adequate flexibility and adhesion properties at temperatures as low as −40 °C.^183^ UV dose accumulation varies significantly with geographical location and installation conditions, with high-altitude installations experiencing particularly intense UV exposure that can accelerate polymer degradation and require enhanced stabilization strategies.^182^ Field studies comparing EVA performance across different climatic zones have revealed location-specific degradation patterns, with desert installations showing primarily thermal and UV-induced degradation. At the same time, coastal locations exhibit accelerated corrosion and salt-induced effects.^182^ The interaction between multiple environmental stressors can produce synergistic degradation effects that exceed the sum of individual stress impacts, requiring comprehensive accelerated testing protocols that simulate realistic multi-stress conditions.^181^ Advanced EVA formulations tailored for specific climatic conditions incorporate climate-optimized stabilizer packages. Desert-grade formulations emphasize thermal and UV stability, while tropical-grade systems focus on hydrolysis resistance and corrosion protection.^184^ Long-term field performance data spanning over 25 years has validated the reliability of properly formulated EVA systems across diverse climatic conditions, with well-designed modules maintaining over 80% of initial power output after two decades of operation.^185^

The continued evolution of EVA encapsulation technology focuses on addressing emerging challenges including higher operating temperatures in next-generation solar cells, enhanced UV resistance for space applications, and improved recyclability for sustainable end-of-life management. Advanced EVA systems incorporating nanotechnology, smart additives, and bio-based components represent the next generation of encapsulation materials, enabling solar technology deployment in increasingly demanding applications while maintaining the cost-effectiveness and reliability that have established EVA as the industry standard.^188^ The integration of real-time monitoring capabilities and self-healing mechanisms in future EVA systems promises to enhance module reliability further and enable predictive maintenance strategies that optimize solar installation performance over extended operational lifetimes.^178^

### 4.2. Advanced encapsulants

The evolution of encapsulation materials represents a critical advancement in solar cell technology, with next-generation polymeric systems offering superior performance characteristics compared to traditional ethylene vinyl acetate (EVA) materials. Advanced encapsulants play a multifaceted role in photovoltaic modules, providing mechanical protection, optical clarity, electrical insulation, and long-term stability while enabling new device architectures and manufacturing processes. The need for enhanced durability, reduced degradation rates, and improved compatibility with emerging solar cell technologies has driven the transition from conventional encapsulants to advanced polymer systems.

#### 4.2.1. Polyolefin elastomer (POE) systems

Polyolefin elastomers have emerged as leading candidates for next-generation solar cell encapsulation, offering exceptional performance characteristics that address many limitations of traditional EVA systems. POE materials demonstrate superior UV resistance, with studies showing minimal yellowing and degradation of mechanical properties after extended exposure to accelerated weathering conditions equivalent to 25 years of outdoor deployment.^79^ The chemical structure of POE, comprising ethylene–octene copolymers with controlled crystallinity, offers an optimal balance between flexibility and dimensional stability, which is essential for long-term module reliability.

The optical properties of POE encapsulants represent a significant advancement over conventional materials, with transmittance values consistently exceeding 95% across the solar spectrum while maintaining stability under prolonged UV exposure. Advanced POE formulations incorporate UV-absorbing additives and antioxidant packages that effectively prevent photodegradation without compromising optical clarity. These materials exhibit excellent adhesion to both glass and backsheet materials, creating robust interfacial bonds that resist delamination under thermal cycling and mechanical stress conditions.

POE encapsulants demonstrate exceptional barrier properties, with WVTRs as low as 10^−6^ g m^−2^ per day, representing a two-order-of-magnitude improvement over standard EVA systems.^202^ This ultra-low permeability effectively prevents moisture ingress that can lead to corrosion of metallic components and degradation of organic materials within the module. The superior barrier performance is attributed to the semi-crystalline structure of POE, which creates tortuous diffusion paths that significantly reduce vapor transport rates.

#### 4.2.2. Thermoplastic polyurethane (TPU) encapsulants

Thermoplastic polyurethane systems offer unique advantages for specialized solar cell applications, particularly in flexible and lightweight photovoltaic modules where mechanical compliance is essential. TPU encapsulants provide exceptional elasticity and tear resistance, with elongation-at-break values exceeding 500% while maintaining optical transparency above 90%.^105^ The segmented block copolymer structure of TPU, consisting of alternating hard and soft segments, enables tailored mechanical properties through careful selection of polyol and isocyanate components.

The adhesive properties of TPU encapsulants eliminate the need for separate adhesive layers in many applications, simplifying module construction and reducing manufacturing costs. Advanced TPU formulations exhibit excellent adhesion to various substrate materials, including polymer films, metal foils, and glass surfaces, with peel strength values exceeding 50 N cm^−1^ after thermal aging.^203^ The thermoplastic nature of TPU enables reprocessing and recycling, addressing growing concerns about end-of-life module disposal and supporting circular economy principles in photovoltaic manufacturing.

TPU encapsulants exhibit superior low-temperature flexibility compared to thermoset alternatives, maintaining mechanical integrity at temperatures as low as −40 °C without brittle failure. This characteristic is particularly valuable for applications in harsh climatic conditions where thermal cycling can induce significant mechanical stress in rigid encapsulant systems.^204^ The excellent low-temperature performance is attributed to the glass transition temperature of the soft segment, which can be tailored through the selection of polyols to optimize flexibility for specific applications.

#### 4.2.3. Silicone-based encapsulation systems

Silicone-based encapsulants represent the premium tier of advanced encapsulation materials, offering unparalleled thermal stability and chemical resistance for high-performance solar cell applications. These systems demonstrate exceptional temperature stability, maintaining mechanical and optical properties at operating temperatures exceeding 150 °C while showing minimal degradation under accelerated aging conditions.^190^ The siloxane backbone provides inherent UV resistance and oxidative stability, making silicone encapsulants particularly suitable for concentrator photovoltaic systems and high-temperature applications.

The refractive index of silicone encapsulants can be precisely controlled through chemical modification, allowing for the optimization of optical coupling between cell surfaces and encapsulant materials. Advanced silicone formulations achieve refractive indices ranging from 1.35 to 1.55, enabling the minimization of reflection losses and the maximization of light transmission to the active cell area.^205^ This optical tunability, combined with exceptional clarity and color stability, makes silicone encapsulants ideal for high-efficiency cell architectures where optical losses must be minimized.

Silicone encapsulants demonstrate superior hydrophobic characteristics, with water contact angles exceeding 110°, which contribute to self-cleaning properties and reduced soiling losses in outdoor applications. The hydrophobic nature also provides excellent electrical insulation properties, with volume resistivity values above 10^15^ Ω cm maintained even under humid conditions.^166^ Advanced silicone systems incorporate conductive pathways for static charge dissipation while maintaining bulk insulation properties, addressing potential-induced degradation concerns in high-voltage photovoltaic systems.

#### 4.2.4. Performance comparison and selection criteria

The selection of appropriate encapsulant materials requires careful consideration of multiple performance criteria including optical transmission, mechanical properties, barrier characteristics, thermal stability, and cost considerations. Comparative analysis reveals that POE systems offer the best balance of performance and cost for mainstream silicon photovoltaic applications, with superior barrier properties and UV resistance compared to EVA at only marginally higher material costs.^178^ TPU encapsulants offer unique advantages for flexible and lightweight applications where mechanical compliance is crucial, albeit at higher material costs and with some limitations in high-temperature stability.

Silicone-based systems represent the premium option for demanding applications where thermal stability and long-term reliability are paramount, justifying higher material costs through extended service life and reduced maintenance requirements. The choice between these advanced encapsulant systems depends on specific application requirements, with POE systems suitable for standard installations, TPU for flexible applications, and silicone for high-performance and high-temperature environments.^206^

### 4.3. Degradation mechanisms and mitigation

The long-term performance and reliability of polymer-based solar cell components are fundamentally governed by various degradation mechanisms that can significantly impact device efficiency and service life. Understanding these degradation pathways and implementing effective mitigation strategies is crucial for achieving the 25-year performance warranties expected in commercial photovoltaic applications. Advanced polymer systems in solar cells face multiple environmental stressors including elevated temperatures, UV radiation, moisture, oxygen exposure, and mechanical stress, each contributing to specific degradation mechanisms that must be addressed through careful materials design and processing optimization.

The complexity of degradation in polymer-based solar cells arises from the interconnected nature of various failure modes, where primary degradation mechanisms can accelerate secondary processes, leading to cascading failures that compromise overall module performance. Modern approaches to degradation mitigation involve multi-layered strategies combining intrinsic material stability, protective additives, barrier systems, and advanced processing techniques to create robust polymer systems capable of withstanding decades of outdoor exposure while maintaining acceptable performance levels.

Thermal degradation represents one of the most significant challenges in polymer-based solar cell systems, with elevated operating temperatures accelerating various chemical processes that compromise material properties and device performance. The thermal degradation of polymers in photovoltaic applications typically follows complex reaction pathways involving chain scission, crosslinking, and oxidative processes that alter molecular structure and consequently affect optical, electrical, and mechanical properties.^179^ Understanding these pathways is essential for developing thermally stable polymer systems and implementing effective mitigation strategies.

Primary thermal degradation mechanisms in polymer solar cell components include random chain scission, which reduces molecular weight and compromises mechanical properties, and crosslinking reactions that can increase brittleness and reduce flexibility. In organic photovoltaic materials, thermal degradation often manifests as morphological changes in the active layer, where phase separation between donor and acceptor components can occur at elevated temperatures, resulting in a reduced interfacial area and decreased charge generation efficiency.^207^ These morphological instabilities are particularly problematic in bulk heterojunction systems where optimal nanoscale morphology is critical for device performance.

The temperature dependence of degradation rates in polymer systems typically follows Arrhenius behavior, with reaction rates doubling for every 10 °C increase in temperature. This relationship has profound implications for solar cell applications, where module temperatures can exceed 80 °C during peak solar irradiance conditions. Advanced polymer systems designed for high-temperature stability incorporate thermally stable backbone structures, such as aromatic polyimides and polybenzoxazoles, which maintain structural integrity at temperatures exceeding 300 °C.^202^ These materials exhibit significantly reduced degradation rates compared to conventional aliphatic polymers, allowing for operation at elevated temperatures without substantial performance loss.

Mitigation strategies for thermal degradation include the incorporation of thermal stabilizers, such as hindered phenolic antioxidants and phosphite-based processing stabilizers, which scavenge free radicals and prevent chain scission reactions. Advanced stabilizer packages often combine multiple mechanisms, including primary antioxidants that break radical chain reactions and secondary antioxidants that decompose hydroperoxides before they can initiate further degradation.^208^ Heat-resistant polymer formulations also benefit from careful molecular design, incorporating thermally stable linkages and avoiding weak bonds that are susceptible to thermal cleavage.

UV-induced yellowing represents a critical degradation mechanism in polymer-based solar cell systems, where prolonged exposure to ultraviolet radiation leads to the formation of chromophoric groups that absorb visible light, thereby reducing optical transmission. This phenomenon is particularly problematic in encapsulant materials and transparent electrodes, where maintained optical clarity is essential for efficient light transmission to the active cell area. The yellowing process typically involves photochemical reactions that create conjugated systems and carbonyl groups, which shift the absorption spectrum into the visible region and cause the characteristic yellow to brown discoloration observed in aged polymer systems.^185^

The mechanism of UV-induced yellowing in polymer systems involves the absorption of UV photons by chromophoric groups or impurities, leading to the formation of excited states that can undergo various photochemical reactions. These reactions often include photo-oxidation processes that create carbonyl groups, chain scission reactions that generate radical species, and crosslinking reactions that form complex chromophoric networks. In polyolefin-based encapsulants, UV exposure can lead to the formation of conjugated polyene sequences through sequential hydrogen abstraction and radical coupling reactions, resulting in progressive yellowing that can reduce light transmission by 5–10% over the module lifetime.^209^

The wavelength dependence of UV-induced degradation varies significantly among different polymer systems, with most organic materials showing peak sensitivity in the UV-B region (280–320 nm) where solar radiation intensity is sufficient to drive photochemical reactions. Advanced UV-stable polymer formulations incorporate UV-absorbing additives that compete with the polymer matrix for UV photons, effectively protecting the polymer backbone from photochemical attack. Common UV absorbers include benzotriazole derivatives, benzophenone compounds, and triazine-based systems, each offering specific advantages for different polymer systems and application requirements.^210^

Mitigation strategies for UV-induced yellowing encompass both intrinsic material design and additive-based approaches. Intrinsically UV-stable polymers incorporate aromatic structures with delocalized π-electron systems that can dissipate absorbed UV energy through non-radiative processes without undergoing chemical reaction. Examples include polycarbonate and polyethylene terephthalate systems with enhanced UV stability through molecular modification.^211^ Additive-based approaches utilize UV absorbers, hindered amine light stabilizers (HALS), and quenchers that work synergistically to prevent UV-induced degradation while maintaining optical clarity.

Hydrolysis represents a significant degradation mechanism in polymer-based solar cell systems, particularly affecting materials with hydrolyzable bonds such as polyesters, polyurethanes, and specific encapsulant systems. The hydrolysis process involves the cleavage of chemical bonds through a reaction with water molecules, leading to chain scission, a reduction in molecular weight, and a progressive deterioration of mechanical and barrier properties. This degradation mechanism is accelerated at elevated temperatures and in the presence of acidic or basic catalysts, making it particularly relevant for outdoor photovoltaic applications where modules are exposed to varying humidity conditions and temperature fluctuations.^188^

The kinetics of hydrolysis in polymer systems depend on multiple factors, including the chemical structure of the polymer backbone, the presence of hydrolyzable groups, environmental conditions, and the availability of water molecules. In polyester-based systems, hydrolysis typically occurs at ester linkages, leading to the formation of carboxylic acid and alcohol end groups that can catalyze further hydrolysis reactions in an autocatalytic process. This self-accelerating degradation can lead to rapid deterioration once initiated, making prevention strategies critical for long-term reliability.^212^

Corrosion prevention in polymer-based solar cell systems involves protecting metallic components from moisture ingress and creating chemically inert environments that prevent electrochemical reactions from occurring. Advanced barrier systems utilize multiple layers of polymer materials with complementary properties to create tortuous diffusion paths, significantly reducing moisture transmission rates. Ultra-barrier encapsulants based on polyolefin elastomers achieve WVTRs as low as 10^−6^ g m^−2^ per day, effectively preventing moisture-induced corrosion of metallic grid lines and interconnects.^213^

Mitigation strategies for hydrolysis and corrosion include the development of hydrolysis-resistant polymer chemistries, such as polyolefin and fluoropolymer systems that lack hydrolyzable bonds, and the incorporation of molecular sieves and desiccants that actively remove moisture from the module interior. Advanced encapsulant systems also incorporate corrosion inhibitors and pH buffers that neutralize acidic species, thereby maintaining chemically stable environments around sensitive components. Edge sealing technologies using moisture-curing sealants provide additional protection against moisture ingress while allowing for thermal expansion and contraction during temperature cycling.^209^

The complexity of degradation mechanisms in polymer-based solar cell systems necessitates integrated mitigation approaches that simultaneously address multiple failure modes while maintaining overall system performance and cost-effectiveness. Modern degradation mitigation strategies integrate advances in materials science with processing optimization and system-level design considerations to create robust polymer systems that meet long-term reliability requirements. These integrated approaches recognize that individual degradation mechanisms often interact synergistically, where the presence of one degradation mode can accelerate others, necessitating comprehensive protection strategies.^79^

Advanced polymer formulations for solar cell applications typically incorporate multiple additive systems working in concert to provide comprehensive protection against various degradation mechanisms. These systems may include primary and secondary antioxidants for thermal stability, UV absorbers and light stabilizers for photochemical protection, and moisture scavengers to prevent hydrolysis. The optimization of these additive packages requires careful consideration of potential interactions and compatibility issues, as some combinations can lead to antagonistic effects that reduce overall protection efficacy.^214^

## 5. Role of polymeric materials in emerging solar technologies

The integration of polymeric materials into emerging solar technologies represents a paradigm shift in photovoltaic device design, enabling new architectures and performance capabilities that were previously unattainable with traditional inorganic materials. As next-generation solar technologies continue to evolve, polymers play increasingly critical roles in addressing fundamental challenges related to device stability, manufacturing scalability, and cost-effectiveness. These emerging technologies, including perovskite solar cells, organic–inorganic hybrid systems, and flexible photovoltaic architectures, rely heavily on the unique properties of polymeric materials to achieve breakthrough performance metrics while maintaining commercial viability. The multifunctional nature of polymers in emerging solar technologies extends beyond traditional roles as passive components to include active participation in charge transport, light management, and device protection. Advanced polymer systems exhibit remarkable versatility in adapting to the specific requirements of various solar cell architectures, providing tailored properties that address the unique challenges associated with each technology platform. This adaptability, combined with the potential for low-cost solution processing and roll-to-roll manufacturing, positions polymers as enabling materials for the next generation of photovoltaic technologies.

### 5.1. Perovskite solar cells

Perovskite solar cells have rapidly advanced from initial efficiencies of around 3.8% in 2009 to certified (PCEs) exceeding 27% in single-junction devices, with the current record standing at 27.0%, verified by the National Renewable Energy Laboratory (NREL) as of 2025,^215^ rivaling the performance of established silicon technologies. The success of perovskite solar cells is intrinsically linked to the integration of advanced polymeric materials that address critical challenges in device stability, charge transport, and environmental protection. Polymers serve multiple essential functions in perovskite architectures, including hole-transport layers, encapsulation systems, and interfacial modification layers, which collectively enable high-performance operation while addressing the inherent instabilities associated with organic–inorganic halide perovskite materials.

The unique properties of perovskite materials, including their exceptional optoelectronic characteristics and solution processability, create both opportunities and challenges for polymer integration. While perovskites offer outstanding light absorption coefficients and long carrier diffusion lengths, their sensitivity to moisture, oxygen, heat, and light exposure necessitates sophisticated protection strategies that leverage the barrier properties and chemical stability of advanced polymer systems. The development of perovskite-compatible polymers requires careful consideration of interface chemistry, thermal stability, and long-term compatibility under operational conditions.

The development of efficient and stable hole transport materials represents a critical advancement in perovskite solar cell technology, with polymeric systems offering significant advantages over traditional small-molecule alternatives in terms of processability, film formation, and long-term stability. Polymeric hole transport materials (HTMs) provide superior morphological stability compared to small-molecule systems, which are prone to crystallization and phase segregation during device operation. The macromolecular nature of polymeric HTMs yields robust, amorphous films that maintain uniform hole transport properties over extended periods, thereby contributing to enhanced device reliability and performance consistency.^183^

Advanced polymeric HTMs based on carbazole, fluorene, and thiophene building blocks have demonstrated (PCEs) exceeding 18–20% in perovskite solar cells, comparable to expensive small-molecule alternatives while offering enhanced thermal stability and reduced material costs. The molecular design of these polymeric systems focuses on optimizing energy level alignment with perovskite materials, typically requiring highest occupied molecular orbital (HOMO) levels between −5.2 and −5.5 eV to ensure efficient hole extraction while maintaining adequate open-circuit voltage. Conjugated polymers such as poly(3-hexylthiophene) derivatives and poly(triarylamine) systems have shown particular promise, with carefully tuned side chain architectures that balance solubility, film-forming properties, and electronic characteristics.^204^

The charge transport properties of polymeric HTMs are governed by both intramolecular and intermolecular charge transfer mechanisms, with polymer molecular weight, regioregularity, and side chain structure playing crucial roles in determining hole mobility and transport efficiency. High-molecular-weight polymers with enhanced π–π stacking interactions demonstrate superior hole transport properties, with mobility values exceeding 10^−3^ cm^2^ V^−1^ s^−1^ achieved in optimized systems. The incorporation of dopant systems, such as lithium bis(trifluoromethanesulfonyl)imide (LiTFSI) and 4-*tert*-butylpyridine, further enhances conductivity and improves interface energetics, though careful optimization is required to prevent negative impacts on device stability.^216^

Interfacial engineering using polymeric HTMs enables precise control over charge extraction dynamics and recombination losses at the perovskite-HTM interface. Self-assembled monolayers and interfacial modification layers based on functionalized polymers create optimal energy level alignment while passivating surface defects that can act as recombination centers. Advanced polymeric HTM systems incorporate functional groups that interact favorably with perovskite surface species, thereby reducing the interface recombination velocity and enhancing charge collection efficiency. These interfacial modifications have demonstrated the ability to enhance open-circuit voltage by up to 100 mV while maintaining high fill factors and photocurrent generation.^193^

Polymers play a pivotal role in the design of hole transport layers (HTLs) and surface modification strategies in perovskite solar cells. Recent progress highlights how polymeric HTLs can enhance interfacial contact, reduce defect density, and improve both efficiency and operational stability. For instance, studies have demonstrated that tailored polymer HTLs not only facilitate efficient charge extraction but also act as passivation layers to suppress ion migration and interfacial recombination.^217^ Furthermore, advanced functional polymers have been engineered with tailored energy levels and hydrophobicity, leading to improved stability against moisture and thermal stress.^218^ In addition, surface treatments using polymeric modifiers have been shown to significantly enhance the crystallinity of perovskite films and passivate defects, resulting in record efficiencies and operational lifetimes.^219^ Collectively, these advances underscore the multifunctional role of polymers as HTLs and interfacial modifiers, providing a powerful route toward stable, high-performance perovskite photovoltaics.

The encapsulation of perovskite solar cells presents unique challenges that distinguish it from conventional photovoltaic technologies, primarily due to the extreme sensitivity of perovskite materials to environmental factors, including moisture, oxygen, elevated temperatures, and UV radiation. Traditional encapsulation approaches developed for silicon solar cells are inadequate for perovskite systems, which can degrade rapidly upon exposure to water vapor concentrations as low as 10 ppm or oxygen levels typical of ambient air. The development of effective encapsulation solutions requires ultra-barrier polymer systems capable of providing (WVTRs) below 10^−6^ g m^−2^ per day while maintaining optical transparency and mechanical integrity over extended periods.^184^

Advanced polymer encapsulation systems for perovskite solar cells utilize multi-layer barrier architectures that combine organic and inorganic components to achieve unprecedented protection levels. These systems typically employ alternating layers of polymer materials with complementary barrier properties, such as polyethylene terephthalate (PET) for mechanical support, aluminum oxide for moisture blocking, and fluoropolymer coatings for chemical resistance. The resulting multilayer structures create tortuous diffusion paths that effectively prevent the ingress of moisture and oxygen while accommodating thermal expansion and mechanical stress during device operation.^210^

The chemical compatibility between encapsulant materials and perovskite components requires careful consideration to prevent adverse reactions that could compromise device performance or stability. Many conventional encapsulant additives, including plasticizers, UV stabilizers, and processing aids, can migrate into perovskite layers and cause degradation or performance loss. Advanced perovskite-compatible encapsulants utilize purified polymer systems with minimal additive content and incorporate getters or scavengers that actively remove potentially harmful species from the device environment. These systems demonstrate exceptional chemical inertness while maintaining the barrier properties essential for long-term stability.^209^

Edge sealing represents a critical aspect of perovskite encapsulation, as the device edges often provide the primary pathway for moisture ingress due to the multilayer device structure and potential interfacial delamination. Advanced edge sealing solutions utilize moisture-curing polyurethane and silicone-based sealants that create robust hermetic seals while accommodating thermal cycling and mechanical stress. These sealants incorporate desiccant particles and corrosion inhibitors, providing additional protection against moisture-induced degradation. The optimization of edge sealing systems has demonstrated the ability to extend device lifetime by orders of magnitude under accelerated aging conditions.^220^

**Fig. 7:**
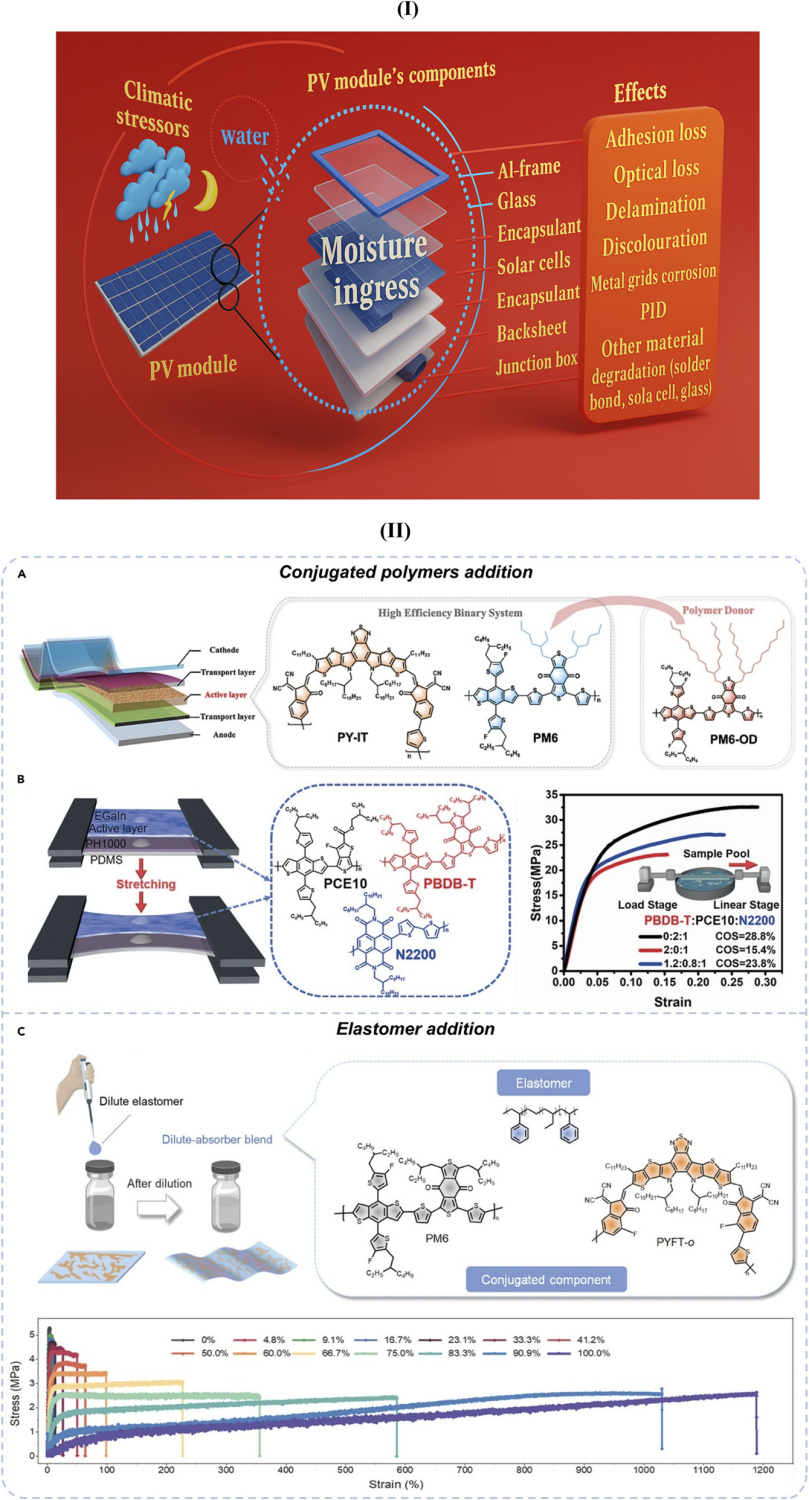
(I) Moisture ingress pathways and degradation mechanisms in photovoltaic (PV) modules. (II) Enhancing mechanical properties *via* third-component incorporation. (A) Incorporation of PM6-OD to improve ductility and stress dissipation. (B) Addition of PCE10 to stabilize N2200 morphology and enhance fracture strain. (C) Use of elastomer SEBS to achieve ultrahigh stretchability in the blend film. Reproduced from ref. 200 with permission from Elsevier, (2025).

Fig. 7(I) illustrates how moisture ingress affects photovoltaic (PV) solar modules and the cascading degradation effects it can cause. Fig. 7(I) illustrates climatic stressors, such as water and weather conditions, that lead to moisture penetration through the various layers of a PV module, including the aluminum frame, glass cover, encapsulant materials, solar cells, backsheet, and junction box. Once moisture infiltrates these components, it triggers a range of detrimental effects shown on the right side of the image, including adhesion loss between layers, optical losses that reduce light transmission, delamination of component interfaces, discoloration of materials, corrosion of metal grid contacts, potential-induced degradation (PID), and broader material degradation affecting solder bonds, solar cells, and glass components. This comprehensive view illustrates how environmental moisture exposure can compromise multiple aspects of solar panel performance and longevity, underscoring the importance of moisture protection in PV module design and installation.

The enhancement of perovskite solar cell stability through polymer integration represents a multifaceted approach that addresses both intrinsic material instabilities and external environmental factors. Polymeric additives and interfacial layers have emerged as powerful tools for improving the fundamental stability of perovskite materials, with carefully designed polymer systems capable of passivating defects, suppressing ion migration, and preventing phase segregation that can lead to device degradation. These stability enhancement strategies often work synergistically with encapsulation approaches to create comprehensive protection systems, enabling the practical deployment of perovskite technologies.^188^

Polymer additives incorporated directly into perovskite precursor solutions have demonstrated remarkable effectiveness in improving crystallization behavior and reducing defect density in the resulting films. Cross-linking polymers such as poly(ethylene glycol) diacrylate create three-dimensional networks within perovskite grain boundaries, effectively immobilizing mobile ions and preventing the migration processes that contribute to device degradation. These polymer networks also provide mechanical reinforcement, reducing the susceptibility of perovskite films to cracking and delamination under thermal stress. Optimized polymer additive concentrations of 1–5 wt% have shown the ability to improve device stability by factors of 10–100 while maintaining high power conversion efficiency.^212^

Interfacial stabilization using polymeric buffer layers represents another critical strategy for enhancing the stability of perovskite solar cells, with these layers serving to protect sensitive perovskite surfaces from environmental exposure while optimizing charge transport characteristics. Polymeric buffer layers based on polyethylenimine (PEI) and its derivatives have demonstrated exceptional effectiveness in passivating surface defects and reducing recombination losses at perovskite-transport layer interfaces. These ultra-thin polymer layers, typically 2–5 nm thick, create favorable interface dipoles that improve band alignment while providing chemical protection against moisture and oxygen exposure.^213^

The development of self-healing polymer systems for perovskite applications represents an emerging frontier in stability enhancement, as these materials are capable of autonomously repairing minor defects and maintaining their barrier properties over extended periods. Self-healing encapsulants based on supramolecular polymers and reversible cross-linking chemistries can recover from mechanical damage and maintain protective functionality even after experiencing stress-induced cracking. These systems incorporate dynamic bonding mechanisms, such as hydrogen bonding and metal coordination, that enable repeated healing cycles without significant degradation of their properties. Prototype self-healing encapsulants have demonstrated the ability to maintain barrier effectiveness after multiple damage-healing cycles, offering potential for extended device lifetimes in challenging environments.^79^

Advanced stability testing protocols for polymer-enhanced perovskite systems utilize accelerated aging conditions that simulate decades of outdoor exposure in compressed timeframes. These protocols examine multiple degradation mechanisms simultaneously, including thermal cycling, UV exposure, damp heat conditions, and mechanical stress testing. The results of these comprehensive stability assessments guide the optimization of polymer systems and processing conditions to maximize long-term reliability. To date, the longest reported outdoor operation for perovskite-based devices concerns perovskite–silicon tandem cells, which retained 80% of their initial efficiency after one year under real-world conditions.^221^ While this is an encouraging step toward long-term reliability, accurate 10-year stability data remain unrealized in peer-reviewed literature. Ongoing research—including accelerated cycling and encapsulation strategies—is steadily closing the gap toward that goal.^222,223^

### 5.2. Organic photovoltaics

Organic photovoltaics (OPV) represent a transformative approach to solar energy conversion, leveraging the unique properties of organic semiconducting materials to create lightweight, flexible, and potentially low-cost photovoltaic devices. The field has witnessed remarkable progress in recent years, with (PCEs) exceeding 18% in single-junction devices and over 20% in tandem configurations, approaching the performance levels required for commercial viability. The success of organic photovoltaics is fundamentally rooted in advanced polymer chemistry and materials design, where precisely engineered donor–acceptor polymer systems enable efficient light absorption, charge separation, and transport processes essential for high-performance operation.

The versatility of organic photovoltaic systems stems from the ability to tailor molecular structures and electronic properties through synthetic chemistry, enabling optimization of key parameters including optical absorption, energy levels, charge mobility, and morphological characteristics. This molecular-level control enables the development of specialized materials for specific applications, ranging from transparent solar cells for building integration to ultra-flexible devices for wearable electronics. The continued advancement of organic photovoltaics depends critically on understanding and controlling the complex relationships between molecular structure, film morphology, and device performance, requiring sophisticated approaches to materials design and processing optimization.

The design of donor–acceptor (D–A) polymers represents the cornerstone of modern organic photovoltaic technology, with these alternating copolymer systems enabling precise control over electronic properties, optical absorption characteristics, and charge transport behavior. The D–A approach involves the strategic combination of electron-rich donor units and electron-deficient acceptor units along the polymer backbone, creating intramolecular charge transfer states that facilitate efficient light absorption across the solar spectrum while maintaining appropriate energy levels for charge separation at the donor–acceptor interface. This design strategy has enabled the development of high-performance polymer systems with (PCEs) exceeding 18% in single-junction devices.^183^

The molecular design principles governing D–A polymer performance involve the careful optimization of multiple structural parameters, including the choice of donor and acceptor units, the ratio of these components, side-chain architecture, and molecular weight characteristics. Common donor units include benzodithiophene (BDT), thieno[3,4-*b*]thiophene (TT), and carbazole derivatives, while acceptor units frequently incorporate benzothiadiazole (BT), diketopyrrolopyrrole (DPP), and quinoxaline moieties. The electronic properties of the resulting copolymers can be systematically tuned by modifying these building blocks, enabling the optimization of the highest occupied molecular orbital (HOMO) and lowest unoccupied molecular orbital (LUMO) energy levels to maximize open-circuit voltage while maintaining efficient charge separation.^204^

The optical properties of D–A polymers are governed by the strength of intramolecular charge transfer interactions, which can be controlled through the electron-donating and electron-accepting characteristics of the constituent units. Strong D–A interactions typically result in narrow bandgap polymers with broad absorption spectra extending into the near-infrared region. In contrast, weaker interactions yield wider bandgap materials with absorption primarily in the visible spectrum. The optimization of absorption characteristics requires balancing spectral coverage with other critical properties such as charge mobility and morphological stability. Advanced D–A polymer systems achieve absorption coefficients exceeding 10^5^ cm^−1^ while maintaining hole mobilities above 10^−3^ cm^2^ V^−1^ s^−1^, demonstrating the successful integration of optical and electronic optimization.^216^

Side chain engineering plays a crucial role in D–A polymer design, with alkyl chain length, branching, and functionalization significantly affecting solubility, film-forming properties, and intermolecular interactions. Linear alkyl chains promote strong π–π stacking interactions that enhance charge transport but may lead to excessive crystallization, which disrupts the optimal morphology in bulk heterojunction devices. Branched side chains provide enhanced solubility and processing characteristics while maintaining sufficient intermolecular interactions to facilitate efficient charge transport. The incorporation of functional side chains, such as those containing fluorine atoms or polar groups, enables fine-tuning of molecular packing and interface energetics. Recent advances in side chain design have demonstrated the ability to achieve optimal morphology control while maintaining high charge mobility and device stability.^193^

The control of active layer morphology represents one of the most critical factors determining the performance of organic photovoltaic devices, with optimal morphology requiring nanoscale phase separation between donor and acceptor components to maximize the interfacial area while ensuring continuous pathways for charge transport to the respective electrodes. The bulk heterojunction architecture, where donor and acceptor materials are intimately mixed throughout the active layer, depends on achieving optimal domain sizes of 10–20 nm to ensure efficient exciton dissociation while maintaining percolated networks for charge collection. This morphological optimization requires sophisticated processing approaches that balance thermodynamic and kinetic factors governing phase separation during film formation.^184^

Solvent engineering is a primary tool for controlling morphology in organic photovoltaic devices, with the choice of processing solvents significantly affecting the kinetics of phase separation and crystallization during film drying. High-boiling-point solvents, such as 1,8-diiodooctane (DIO) and 1-chloronaphthalene (CN), serve as processing additives that selectively solvate specific components and control the rate of phase separation, thereby enabling the formation of optimal domain sizes and interfacial structures. The concentration of these additives, typically 1–5 vol%, must be carefully optimized to achieve the desired morphology while avoiding residual additive content that could compromise device stability. Advanced solvent systems utilize binary and ternary mixtures that provide independent control over different aspects of morphology formation.^210^

Thermal annealing protocols offer another powerful approach for morphology optimization, with carefully controlled heating treatments enabling post-deposition optimization of domain size, crystallinity, and interfacial characteristics. The temperature and duration of annealing treatments must be optimized for each donor–acceptor system, with typical conditions ranging from 80 °C to 150 °C for 5–30 minutes. The annealing process can promote beneficial reorganization of molecular packing and reduce energetic disorder, leading to improved charge transport and reduced recombination losses. However, excessive annealing can lead to over-crystallization and phase segregation, which reduces the interfacial area and compromises device performance. Advanced annealing protocols utilize ramped temperature profiles and controlled cooling rates to achieve optimal morphology while maintaining device stability.^209^

The development of non-fullerene acceptors has revolutionized morphology control in organic photovoltaics, with these materials offering greater flexibility in molecular design and processing compared to traditional fullerene derivatives. Non-fullerene acceptors such as ITIC derivatives and Y6-based systems can be designed with specific molecular structures that promote favorable mixing with donor polymers while maintaining appropriate energy level alignment for efficient charge separation. The strong absorption characteristics of these acceptors also enable the development of complementary absorption profiles that maximize light harvesting efficiency. The optimization of donor–acceptor combinations has led to devices with (PCEs) exceeding 18%, demonstrating the critical importance of morphology control in achieving high performance.^220^

Tandem cell architectures represent the most promising approach for achieving high (PCEs) in organic photovoltaics, with these multi-junction devices capable of overcoming the fundamental limitations of single-junction cells through complementary absorption of different portions of the solar spectrum. The tandem approach involves stacking multiple sub-cells with different bandgaps, enabling more efficient utilization of solar photons and theoretical efficiency limits exceeding 30% for triple-junction configurations. Recent advances in tandem organic photovoltaic devices have demonstrated (PCEs) above 20%, representing a significant milestone toward commercial viability.^188^

The design of tandem cell architectures requires careful optimization of multiple parameters, including the band gaps of constituent sub-cells, the optical and electrical properties of intermediate layers, and the overall device architecture to maximize current matching and minimize optical and electrical losses. The front sub-cell typically utilizes a wide-bandgap donor–acceptor system optimized for absorption in the visible spectrum. In contrast, the rear sub-cell employs a narrow-bandgap system designed for efficient absorption in the near-infrared range. The bandgap combination must be optimized to achieve current matching between sub-cells while maximizing the overall photocurrent generation. Optimal bandgap combinations for two-junction devices typically involve front cell bandgaps of 1.6–1.8 eV and rear cell bandgaps of 1.2–1.4 eV.^212^

The intermediate connecting layer between sub-cells in tandem architectures plays a critical role in device performance, serving multiple functions including optical coupling, charge recombination, and electrical isolation between sub-cells. Advanced intermediate layers utilize transparent conductive oxides such as zinc oxide or molybdenum oxide combined with ultra-thin metallic layers to achieve optimal optical and electrical properties. These layers must provide efficient recombination of holes from the front cell with electrons from the rear cell while maintaining high optical transparency and an appropriate refractive index for optical coupling. The thickness and composition of intermediate layers must be precisely controlled to minimize optical losses while ensuring efficient charge recombination. Recent developments in intermediate layer design have achieved transmittance values exceeding 90% while maintaining excellent electrical connectivity.^213^

The processing of tandem organic photovoltaic devices requires sophisticated approaches to avoid damage to underlying layers during the deposition of subsequent components. Solution-processed tandem devices must utilize orthogonal solvent systems that enable deposition of upper layers without dissolving or damaging previously deposited films. This constraint has driven the development of cross-linkable polymer systems and water-based processing approaches, enabling the fabrication of multi-layer devices. Alternatively, vacuum-deposited small-molecule systems avoid solvent compatibility issues but require more complex processing equipment and may have limitations in terms of controlling active layer thickness and morphology. Hybrid approaches that combine solution-processed and vacuum-deposited layers offer potential advantages in terms of processing flexibility and performance optimization.^79^ The optical design of tandem cells requires careful consideration of light management to maximize absorption in each sub-cell while minimizing reflection and parasitic absorption losses. Advanced optical modeling approaches utilize transfer matrix methods and finite-element analysis to optimize layer thicknesses and refractive indices for maximum light utilization. The incorporation of light-trapping structures such as textured interfaces and photonic crystals can further enhance light absorption and improve device performance. Recent advances in optical design have demonstrated the ability to achieve near-optimal light distribution between sub-cells while maintaining practical device architectures suitable for large-scale manufacturing.^209^

The continued advancement of organic photovoltaic technology depends on addressing the remaining challenges in device efficiency, stability, and manufacturing scalability, while maintaining the unique advantages of organic systems, including mechanical flexibility, lightweight characteristics, and the potential for low-cost production. Recent progress in donor–acceptor polymer design has demonstrated that further efficiency improvements are possible through continued optimization of molecular structures and processing conditions. The development of next-generation acceptor materials with enhanced stability and broader absorption characteristics offers potential for devices with efficiencies exceeding 20% in single-junction configurations.^207^

The stability of organic photovoltaic devices remains a critical challenge for commercial deployment, with current state-of-the-art devices demonstrating operational lifetimes of several thousand hours under accelerated aging conditions. The development of inherently stable polymer systems and advanced encapsulation technologies is essential for achieving the 25-year operational lifetimes required for commercial viability. Recent advances in stability enhancement include the development of photo-stable donor–acceptor polymers and ultra-barrier encapsulation systems that effectively protect devices from exposure to moisture and oxygen. The integration of self-healing materials and adaptive protection systems offers potential for further improvements in long-term stability.^208^

### 5.3. Stretchable solar cells

Stretchable solar cells represent a revolutionary advancement in photovoltaic technology, enabling the integration of energy-harvesting capabilities into applications that require extreme mechanical deformation, such as wearable electronics, soft robotics, and biomedical devices. Unlike conventional flexible solar cells, which can bend while maintaining their planar dimensions, stretchable photovoltaic devices must accommodate multi-directional strain while preserving both electrical functionality and optical performance. This capability requires fundamental innovations in materials science, device architecture, and manufacturing processes that go beyond traditional approaches to flexible electronics. The development of stretchable solar cells has been enabled by breakthroughs in intrinsically stretchable semiconducting polymers, advanced mechanical design strategies, and novel device architectures that maintain electrical connectivity under extreme deformation. Stretchable solar cells primarily emphasize mechanical compliance rather than introducing a new class of photovoltaic materials. Their design is centered on enabling the device to withstand significant strains, bends, and twists while maintaining electrical functionality, which is crucial for applications in wearable electronics, soft robotics, and biomedical devices. Polymers and advanced structural engineering play a central role in this approach, providing both flexibility and resilience. By incorporating innovative architectures such as wavy, serpentine, or island–bridge layouts, stretchable photovoltaics can accommodate multi-directional deformation, ensuring stable performance under mechanical stress. Consequently, the focus of stretchable solar cells lies in integrating conventional or flexible photovoltaic materials into mechanically robust systems rather than altering the fundamental semiconductor properties.

The unique challenges associated with stretchable solar cells stem from the inherent conflict between the electronic properties required for efficient photovoltaic operation and the mechanical properties necessary for stretchability. Traditional semiconducting materials exhibit brittle behavior and lose electrical functionality under strain, while elastomeric materials that provide stretchability typically lack the electronic characteristics necessary for photovoltaic applications. Resolving this fundamental trade-off requires innovative approaches to materials design and device engineering that enable high electronic performance while maintaining mechanical robustness under repeated deformation cycles. Recent advances have demonstrated stretchable solar cells capable of maintaining over 80% of their initial efficiency under 50% strain, representing a significant milestone toward practical applications. The development of intrinsically stretchable semiconducting polymers represents a paradigm shift in organic electronics, enabling the creation of materials that maintain electronic functionality while accommodating large mechanical deformations. These advanced polymer systems achieve stretchability through carefully designed molecular architectures that incorporate flexible spacer units, dynamic cross-linking mechanisms, and optimized side-chain structures, enabling polymer chain mobility without compromising conjugation and charge transport properties. The most successful approaches utilize donor–acceptor copolymer systems where flexible non-conjugated segments are strategically incorporated between rigid semiconducting blocks, creating materials that can stretch up to 100% while maintaining charge carrier mobilities above 10^−3^ cm^2^ V^−1^ s^−1^.^183^

The molecular design of intrinsically stretchable polymers requires careful optimization of multiple structural parameters including the ratio of rigid to flexible segments, the chemical nature of flexible spacers, and the overall polymer architecture. Conjugated polymers with incorporated flexible alkyl chains, siloxane segments, or dynamic covalent bonds demonstrate exceptional stretchability while maintaining reasonable electronic properties. Advanced systems utilize diketopyrrolopyrrole (DPP) and benzothiadiazole (BT) acceptor units connected through flexible linkers, such as poly(ethylene glycol) or siloxane chains, which enable strain accommodation without disrupting the electronic structure of the semiconducting units. The optimization of these molecular architectures has yielded materials with ultimate elongation exceeding 200% while maintaining (PCEs) above 6% in photovoltaic devices.^204^

The charge transport mechanisms in intrinsically stretchable polymers differ significantly from those in conventional rigid semiconductors, with transport often occurring through a combination of intramolecular conduction along polymer chains and intermolecular hopping between polymer segments. The presence of flexible spacers creates conformational freedom, enabling polymer chains to maintain electrical connectivity even under significant strain. However, this increased disorder can lead to reduced charge carrier mobility compared to rigid analogues. Advanced polymer designs incorporate multiple transport pathways and self-healing mechanisms, enabling the recovery of electrical properties after strain release. The incorporation of dynamic bonds, such as hydrogen bonding or π–π stacking interactions, provides additional pathways for charge transport while enabling mechanical compliance.^216^

Surface and interface engineering play a critical role in optimizing the performance of intrinsically stretchable polymers, as processing conditions and substrate interactions strongly influence the mechanical properties of polymer films. The development of stretchable polymer films requires careful control of molecular orientation, crystallinity, and interfacial adhesion to achieve optimal mechanical and electronic properties. Advanced processing techniques utilize controlled solvent evaporation, thermal annealing, and mechanical pre-stretching to optimize polymer chain alignment and reduce defect density. The incorporation of plasticizers and compatibilizers enables fine-tuning of mechanical properties while maintaining electronic functionality. Recent advances have demonstrated the ability to achieve reversible stretchability exceeding 300% in optimized polymer systems while maintaining stable photovoltaic performance over thousands of stretch-release cycles.^193^

The mechanical design of stretchable solar cells necessitates innovative approaches that extend beyond materials development to encompass device architecture, electrode design, and encapsulation strategies, enabling stable operation under extreme mechanical deformation. The fundamental challenge lies in creating device structures that can accommodate large strains while maintaining electrical connectivity and preventing mechanical failure of critical components. Advanced mechanical design strategies leverage concepts from soft robotics and biomimetic engineering to create device architectures that effectively distribute mechanical stress while preserving electronic functionality. These approaches often involve the development of serpentine interconnects, wavy electrode patterns, and hierarchical structures that enable large-scale deformation through localized strain accommodation.^184^

Electrode design represents a critical aspect of stretchable solar cell engineering, as conventional metal electrodes exhibit limited stretchability and are prone to cracking under strain. Advanced electrode systems utilize intrinsically stretchable conducting polymers, liquid metal alloys, and hybrid composite structures that maintain conductivity under large deformations. Conducting polymers such as (PEDOT:PSS) can be modified with plasticizers and surfactants to achieve stretchability exceeding 100% while maintaining sheet resistances below 100 Ω sq^−1^. Liquid metal electrodes based on gallium–indium alloys offer exceptional stretchability and self-healing properties; however, they require sophisticated encapsulation to prevent oxidation and maintain electrical contact. Hybrid approaches that combine conducting polymers with metallic nanowires or carbon nanotubes offer an optimal balance between conductivity and stretchability.^210^

The encapsulation of stretchable solar cells presents unique challenges compared to conventional flexible devices, requiring materials and designs that provide environmental protection while accommodating large mechanical deformations. Advanced encapsulation systems utilize elastomeric materials with tailored barrier properties, including silicone-based polymers, polyurethane elastomers, and thermoplastic elastomers that maintain protective functionality under strain. The encapsulation design must prevent moisture and oxygen ingress while allowing for thermal expansion and mechanical deformation without delamination or cracking. Multi-layer encapsulation architectures that combine different elastomeric materials with complementary properties have demonstrated the ability to maintain barrier effectiveness under 100% strain while providing long-term environmental protection.^209^

The integration of strain sensors and feedback systems represents an emerging approach to mechanical design that enables real-time monitoring of device deformation and adaptive control of operating parameters. These smart, stretchable solar cells incorporate embedded sensors that monitor strain distribution and electrical performance, enabling the optimization of device operation based on its mechanical state. Advanced systems utilize machine learning algorithms to predict optimal operating conditions based on strain patterns and environmental conditions, maximizing energy harvesting efficiency while preventing mechanical failure. The integration of self-healing materials and adaptive protection systems offers potential for autonomous repair of minor mechanical damage and extended device lifetimes under harsh operating conditions.^220^

Fig. 7(II)A illustrates the strategic incorporation of a stretchable polymer, PM6-OD, into a high-efficiency donor–acceptor blend to enhance the mechanical resilience of the active layer. The PM6-OD polymer possesses long side chains, which not only improve the entanglement density within the polymer network but also promote favorable molecular interactions between chains. This entanglement leads to a more mechanically robust film structure capable of dissipating applied stress during mechanical deformation. The inclusion of PM6-OD helps maintain the photovoltaic performance while significantly boosting the stretchability of the active layer. This demonstrates that side-chain engineering and judicious molecular design of the donor polymer can simultaneously achieve high efficiency and mechanical adaptability, which is crucial for emerging applications such as flexible or wearable photovoltaics. The study by Li *et al.*^224^ highlights how morphology retention and mechanical improvement can go hand-in-hand when selecting appropriate third components.

Fig. 7(II)B depicts the introduction of PCE10 as a third component into the PBDB-T:N2200 binary blend to fine-tune the nanoscale morphology and improve mechanical properties. The presence of PCE10 interacts synergistically with N2200, a polymer acceptor known for its fibrous network structure. Through molecular interaction, PCE10 helps regulate and stabilize this network, enhancing the film's ductility without significantly disturbing charge transport pathways. Notably, the optimized ternary blend achieves a fracture strain of 23.8%, a remarkable improvement over the conventional system. This suggests that the molecular compatibility between PCE10 and N2200 allows for structural integrity under mechanical stress. As demonstrated by Zhu *et al.*,^225^ this approach leverages morphology control through third-component engineering to balance mechanical flexibility and device efficiency.

Fig. 7(II)C showcases the use of a stretchable elastomer, SEBS (styrene–ethylene–butylene–styrene), as a third component to achieve unprecedented mechanical elongation in the blend film. Unlike conjugated polymers, SEBS is an insulating elastomer that offers extreme stretchability. While traditional donor–acceptor systems exhibit limited tensile strain, the incorporation of SEBS into the all-polymer active layer drastically enhances the fracture strain to nearly 1200%. This impressive improvement suggests that SEBS acts as a mechanical buffer, distributing strain across the film and preventing localized failure. However, the non-conjugated nature of SEBS requires careful control of phase separation to prevent hindrance of charge transport. According to Li *et al.*,^226^ this innovative strategy demonstrates the feasibility of using hybrid mechanical–photovoltaic engineering to develop ultra-flexible devices with high endurance under repeated strain cycles.

Fig. 7(II)D involves the integration of IDTBT (poly(indacenodithiophene-*co*-benzothiadiazole))—a nearly amorphous, highly stretchable conjugated polymer—into a layer-by-layer (LbL) active layer architecture. IDTBT is known for its excellent fracture strain (∼80%) and minimal crystallinity, which allows it to accommodate mechanical deformation while maintaining favorable electrical pathways. The use of LbL processing permits precise control over vertical phase separation, optimizing both electrical performance and mechanical response. As a result, the intrinsically stretchable all-polymer solar cells (IS-APSCs) achieved a record-breaking PCE of 14.2%, maintaining over 70% efficiency under 50% strain and after multiple deformation cycles. This exemplifies a transformative leap in stretchable photovoltaics, integrating advanced materials and processing techniques to realize devices with high performance and mechanical resilience.

The integration of stretchable solar cells into wearable electronics represents one of the most promising applications for this emerging technology, offering the potential for self-powered devices that can harvest energy from ambient light while conforming to the complex geometries and dynamic movements of the human body. Wearable electronics applications place unique demands on stretchable solar cells, requiring devices that can accommodate the full range of human motion while maintaining electrical performance and user comfort. The development of wearable-compatible, stretchable solar cells has driven advances in ultra-thin device architectures, biocompatible materials, and energy management systems, enabling the practical deployment of these technologies in clothing, accessories, and medical devices.^188^

The mechanical requirements for wearable stretchable solar cells are determined by the specific application and body location, with different applications requiring different levels of stretchability and mechanical robustness. Devices integrated into clothing must accommodate the stretch characteristics of textile materials, typically requiring stretchability of 20–50% in multiple directions while maintaining electrical functionality. Applications in more dynamic environments, such as athletic wear or medical monitoring devices, may require stretchability exceeding 100% along with resistance to repeated deformation cycles. The mechanical design must also consider the interaction between the device and the textile substrate, requiring adhesion systems that maintain electrical contact while allowing for differential strain between the device and fabric.^212^

The power management requirements for wearable, stretchable solar cells differ significantly from those of conventional photovoltaic applications, with energy harvesting often occurring under variable and low-light conditions that necessitate specialized electronic systems for optimal performance. Advanced power management systems utilize maximum power point tracking algorithms optimized for dynamic illumination conditions and incorporate energy storage systems that can accommodate the intermittent nature of wearable energy harvesting. The integration of supercapacitors and thin-film batteries enables storage of harvested energy for use during periods of low illumination. Recent advances have demonstrated wearable systems capable of generating sufficient power for low-power electronics such as sensors, displays, and wireless communication devices.^213^

The biocompatibility and safety considerations for wearable stretchable solar cells require careful attention to materials selection and device design to ensure user safety and comfort during extended wear. The encapsulation materials must be non-toxic, hypoallergenic, and breathable to prevent skin irritation and allow for normal physiological processes to occur. Advanced biocompatible encapsulation systems utilize medical-grade silicones and polyurethane elastomers that have been extensively tested for skin contact applications. The device design must also consider thermal management to prevent overheating during operation and ensure user comfort. The incorporation of temperature monitoring and thermal dissipation systems enables safe operation even under high-intensity illumination conditions.^79^

The manufacturing and integration of stretchable solar cells into wearable electronics requires scalable production methods that are compatible with textile manufacturing processes. Advanced manufacturing approaches utilize roll-to-roll processing, screen printing, and lamination techniques that enable high-volume production of wearable-integrated photovoltaic devices. The integration process must consider the mechanical properties of textile substrates and the thermal stability of device components during the textile processing stage. Recent advances in low-temperature processing and adhesive systems have enabled the integration of stretchable solar cells into conventional garments without compromising the mechanical properties of the textile or the electrical performance of the device. The development of washable and dry-cleanable stretchable solar cells represents an important milestone toward practical wearable applications.^209^

The continued advancement of stretchable solar cell technology requires addressing remaining challenges in efficiency, stability, and manufacturing scalability while maintaining the unique mechanical properties that enable their specialized applications. Current state-of-the-art stretchable solar cells demonstrate power conversion efficiencies (PCEs) of 6–10%, which is significantly lower than those of their rigid counterparts but sufficient for many wearable applications. Further efficiency improvements require the continued development of intrinsically stretchable materials with enhanced electronic properties, as well as the optimization of device architectures that minimize optical and electrical losses under strain. The integration of light-trapping structures and advanced optical design offers potential for significant efficiency improvements while maintaining mechanical compliance.^207^

The long-term stability of stretchable solar cells under repeated mechanical deformation poses a critical challenge for practical applications, as current devices demonstrate stable operation for thousands of stretch-release cycles but require further improvement for applications that require millions of cycles. The development of self-healing materials and adaptive protection systems offers potential for enhanced durability and extended device lifetimes. Advanced characterization techniques that enable real-time monitoring of device performance under mechanical stress provide insights into degradation mechanisms and guide the development of more robust device designs. The integration of artificial intelligence and machine learning approaches enables predictive maintenance and optimization of device operation based on usage patterns and environmental conditions.^208^

### 5.4. Quantum dot solar cells

Quantum dot solar cells (QDSCs) have emerged as one of the most promising third-generation photovoltaic technologies, offering unique advantages through their quantum confinement effects and tunable optical properties. The integration of polymers into these systems has proven to be revolutionary, addressing critical challenges related to stability, charge transport, and device performance. This comprehensive analysis examines the multifaceted role of polymers in quantum dot solar cell technology, drawing upon recent research developments and breakthrough discoveries.

Saranya *et al.*^227^ demonstrated through theoretical modeling that quantum dot-sensitized solar cells incorporating precise atomic structures can achieve enhanced visible light absorption and rapid charge injection when optimized with appropriate interfacial materials. Their work on MgO/TiO_2_(001) quantum dots highlights how polymer matrices can serve as crucial supporting frameworks that maintain the structural integrity of quantum dots while facilitating efficient charge transport pathways. The quantum confinement effect in these nanocrystals allows for bandgap tunability across the entire solar spectrum, a property that becomes even more valuable when combined with polymer stabilization techniques.

The role of polymers extends beyond mere structural support, as evidenced by recent advances in perovskite quantum dot solar cells. Research teams have successfully developed hybrid interfacial architectures in which polymers serve as both protective barriers and facilitators of charge transport. All-inorganic CsPbI_3_ perovskite quantum dots have garnered substantial research interest for photovoltaic applications due to their higher efficiency compared to solar cells using other quantum dot materials.^228^ When combined with polymer matrices, such as PCBM (phenyl-C_61_-butyric acid methyl ester), these systems achieve remarkable (PCEs) while maintaining excellent mechanical stability on both rigid and flexible substrates.^229^

One of the most significant applications of polymers in QDSCs involves their use as solid-state electrolytes, replacing traditional liquid electrolytes that suffer from stability and leakage issues. Liquid electrolytes in quantum dot sensitized solar cells (QDSSCs) cause device packaging and stability issues, and recent work has focused on developing new types of solid-state polymer electrolytes. Solution-processed colloidal quantum dots are emerging photovoltaic materials with tunable infrared bandgaps, and the incorporation of polymer electrolytes has been instrumental in harnessing these properties for practical applications.

The development of solid polymer electrolytes has shown remarkable promise in addressing the volatility and flammability issues associated with liquid electrolytes in electrochemical devices, particularly in dye-sensitized solar cells (DSSCs) and quantum dot-sensitized solar cells (QDSSCs). These polymer-based electrolytes address the critical challenge of maintaining ionic conductivity while providing mechanical stability, a combination that was previously difficult to achieve with conventional electrolyte systems.

The integration of semiconductor quantum dots as third donor–acceptor components in polymer solar cells has opened new avenues for enhanced charge transfer processes. Quantum dots play an important role in third-generation photovoltaics. The key focus on quantum dots is due to their cost-effectiveness, capacity to work in challenging conditions, and their unique optical and electrical properties. This three-component system represents a significant advancement over traditional two-component polymer solar cells, as the quantum dots serve as intermediate energy levels that facilitate more efficient charge separation and collection.

The synergistic relationship between polymers and quantum dots becomes particularly evident in the context of optimizing charge transport. Recent work by Chen *et al*.^230^ on CuInSe_2_ colloidal quantum dots and studies by Dones Lassalle and Dempsey on PbS nanocrystals have demonstrated how optoelectronic properties can be preserved through careful ligand design, while polymer matrices provide the necessary framework for quantum dot dispersion and electrical pathways for charge collection.

Recent breakthrough research has focused on using polymers as advanced encapsulation materials for quantum dot solar cells. Researchers adopt a new ligand to enhance the efficiency and stability of perovskite quantum dot solar cells. Solar cell efficiency increases to 15.3% by correcting distortions on the surface of quantum dots.^231^ These ligand systems often incorporate polymer components that serve as protective barriers against moisture, oxygen, and other environmental factors that can degrade the performance of quantum dots over time.

Recent work by Kumar *et al.*^232^ Studies by Wong and colleagues^233^ on advanced characterization techniques and research on controlling charge carrier dynamics through atomic doping demonstrate the continuing evolution of quantum dot solar cell technology. The integration of polymers serves multiple critical functions, including structural support, charge transport, stability enhancement, and encapsulation, making them essential components for commercial viability.

Quantum dot solar cells (QDSCs) represent a distinct class of emerging photovoltaic technology, separate from perovskite solar cells (PSCs), organic solar cells (OSCs), and stretchable solar cells. While PSCs and OSCs rely primarily on polymeric or hybrid organic–inorganic semiconductors, QDSCs exploit size-tunable semiconductor nanocrystals (*e.g.*, PbS, CdSe, CsPbX_3_ quantum dots) as the light-absorbing layer. Their unique quantum confinement effect enables bandgap tunability, multiple exciton generation, and compatibility with solution processing. Importantly, QDs have also been integrated into PSCs and OSCs as interfacial modifiers or light-harvesting additives, improving charge transport and spectral utilization. In contrast, stretchable solar cells mainly focus on mechanical compliance, where polymers and structural engineering dominate the design. Therefore, QDSCs should be considered as a complementary but independent route within next-generation photovoltaics, while also serving as a functional enhancement strategy when incorporated into perovskite and organic systems.

## 6. Polymeric coatings and functional surfaces for next-generation photovoltaics

The integration of smart coatings and functional surfaces into photovoltaic (PV) technologies represents a significant leap toward adaptive, self-sustaining, and high-performance solar energy systems. These coatings are engineered to provide more than passive protection; they actively respond to environmental stimuli such as temperature, light intensity, humidity, and contaminants, enhancing energy conversion efficiency, extending device longevity, and reducing maintenance needs. As solar cells are increasingly deployed in diverse and harsh environments, smart coatings play a critical role in enabling next-generation photovoltaic modules that are self-cleaning, thermally adaptive, anti-reflective, and responsive to light management strategies.

One of the most widely researched types of smart coatings is the self-cleaning surface, which significantly reduces power loss due to soiling—the accumulation of dust, pollutants, and water droplets. Two main mechanisms are employed: superhydrophobicity and photocatalysis. Superhydrophobic surfaces, often achieved by combining low-surface-energy polymers (*e.g.*, fluoropolymers or polydimethylsiloxane [PDMS]) with micro- or nanostructured topographies, cause water droplets to bead and roll off the surface, thereby carrying away contaminants.^154^ These structures mimic the lotus leaf effect and can maintain optical transparency when properly designed. Photocatalytic self-cleaning surfaces, typically using TiO_2_ nanoparticles embedded in polymer matrices, degrade organic residues under UV illumination, allowing environmental elements, such as wind or rain, to remove the loosened debris. These coatings are particularly relevant in arid or urban areas where manual cleaning is difficult and costly.

Anti-reflective (AR) smart coatings are another important class. In conventional modules, reflection losses at the air–glass or air–polymer interface can reduce incident light by 4–10%. Smart AR coatings, made from polymers with tunable refractive indices or porous nanostructures, reduce reflection by promoting the destructive interference of reflected light waves. These coatings can dynamically adapt to changes in the angle of light incidence or wavelength distribution, optimizing photon capture throughout the day. The incorporation of thermo-responsive or photo-responsive polymers, such as poly(*N*-isopropylacrylamide) (PNIPAm), enables reversible changes in surface morphology or refractive index, enhancing light trapping under variable illumination conditions.^118^ Thermochromic and electrochromic coatings represent another category of smart materials integrated into solar cell systems, particularly in building-integrated photovoltaics (BIPVs). These materials modulate their optical properties—such as transmittance or color—in response to temperature or electrical stimuli. For example, vanadium dioxide (VO_2_) or thermochromic polymers can switch between transparent and reflective states based on ambient temperature, reducing thermal load on buildings and optimizing solar energy use.^152^ Electrochromic smart windows, when combined with solar modules, allow dynamic control of light and heat transmission, reducing reliance on external energy sources for lighting and HVAC systems.

Phase change materials (PCMs) integrated into polymeric back layers or encapsulants can help regulate temperature within the module by absorbing and releasing heat during phase transitions (*e.g.*, melting and solidifying). These systems, embedded in flexible or rigid substrates, prevent thermal hotspots and reduce thermally induced mechanical stress, thereby prolonging the module's lifespan and stabilizing its performance.^116^ Smart polymer blends that exhibit shape-memory effects or self-healing capabilities are also under exploration. For instance, certain polymer matrices can recover from microcracks or delamination caused by mechanical deformation or thermal cycling, thus preserving device integrity without external intervention.

Smart coatings are also being developed for optical down-conversion and up-conversion, which modify the spectral content of incident light better to match the absorption range of the solar cell. Luminescent down-shifting layers (LDS), typically made of polymer matrices doped with rare-earth ions or organic dyes, absorb UV or blue light and re-emit it in the visible range where many photovoltaic materials (*e.g.*, silicon, P3HT, perovskites) have higher absorption efficiency. These coatings not only increase the number of useful photons but also protect UV-sensitive device layers, acting as multifunctional protective films.^155^

Moreover, conductive polymer coatings with integrated sensor functionalities are being explored for real-time performance monitoring. These smart interfaces can detect environmental parameters (*e.g.*, temperature, humidity, mechanical stress) or internal degradation (*e.g.*, moisture ingress, delamination), providing valuable feedback for predictive maintenance and reliability assessment in large-scale solar farms.

**Fig. 8:**
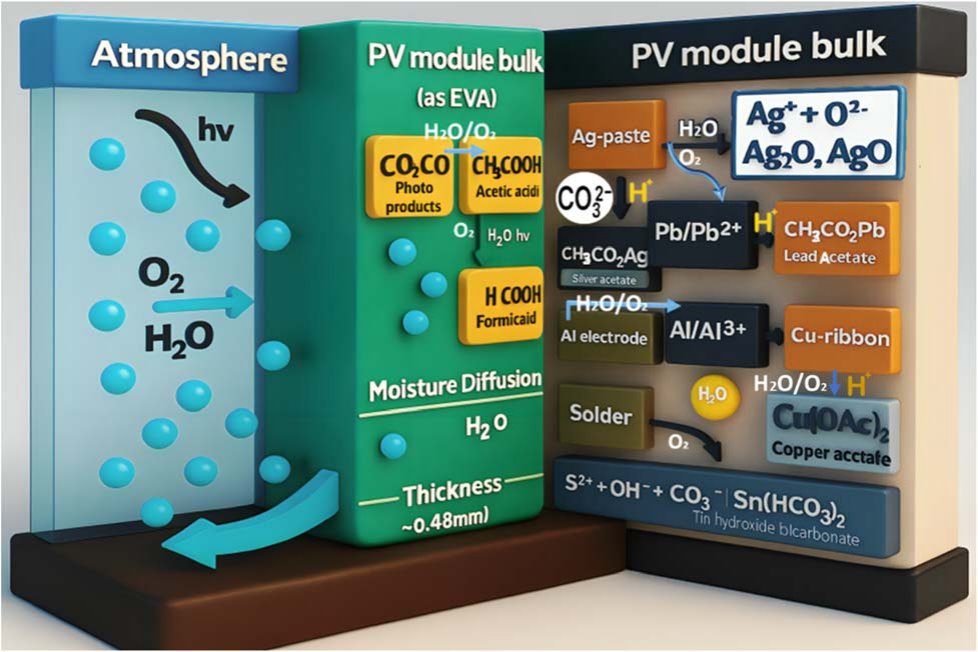
Schematic representation of moisture ingress and degradation mechanisms in photovoltaic (PV) devices. Under sunlight (*hν*), the encapsulant material undergoes photodegradation, generating reactive photoproducts Reproduced from ref. 234 with permission from Elsevier, (2000). These photoproducts interact with infiltrated moisture to form carboxylic acids Reproduced from ref. 235 with permission from Wiley, (2017). Which, along with water, diffuse into the PV module. This initiates multiple degradation pathways involving chemical reactions with internal components such as silver (Ag) from the silver paste, lead (Pb), tin (Sn) from solder, and aluminum (Al) from the rear electrode. Moisture acts as an electrolyte, sustaining electrochemical corrosion and facilitating further degradation of the module's internal structure Reproduced from ref. 239 with permission from OPTICA, (2019).

Fig. 8 illustrates the complex mechanism of moisture ingress in photovoltaic (PV) modules, as well as the subsequent chemical and electrochemical degradation processes that compromise module performance. At the core of this degradation pathway is the interaction between sunlight (denoted as photon energy, *hν*) and the encapsulant material, typically ethylene-vinyl acetate (EVA). Upon prolonged exposure to ultraviolet (UV) radiation, the EVA encapsulant undergoes photodegradation, resulting in the formation of unstable photoproducts, including peroxides, aldehydes, and radical species. These photoproducts are reactive intermediates that further interact with infiltrating moisture, resulting in the formation of carboxylic acids.^234,235^ These acids play a key role in initiating corrosion within the module.

Moisture ingress occurs through the backsheet and encapsulant materials *via* diffusion. The nature of the diffusion mechanism can be broadly categorized into Fickian and non-Fickian diffusion. Fickian diffusion adheres to Fick's laws and is governed primarily by concentration gradients and temperature. This model works well when moisture or gas migrates through homogeneous materials or bulk polymers.^22,236^ However, in some instances, such as when the diffusion is strongly influenced by microchannels, voids, or interfacial regions within the polymeric material, non-Fickian diffusion models offer a more accurate description. The dual-mode or anomalous diffusion models are often used in such scenarios and can represent time-dependent moisture sorption and desorption more realistically.^237,238^

Once inside the module, the moisture and carboxylic acids permeate different layers, reaching metallic contacts and interfaces. These substances serve as electrolytes that facilitate electrochemical reactions leading to corrosion. Specifically, silver (Ag) from the front contact grid, lead (Pb) and tin (Sn) from solder joints, and aluminum (Al) from the rear electrode are prone to oxidation and ion migration under these moist and acidic conditions.^239^ This electrochemical degradation contributes significantly to reduced power output, increased series resistance, delamination, and potential-induced degradation (PID) in PV modules.

The extent of moisture diffusion and its impact depend not only on environmental conditions, such as humidity and temperature, but also on the material properties of the encapsulant and backsheet. Key parameters influencing moisture transport include polymer crystallinity, chemical composition, free volume, polarity, cross-linking density, and the presence of voids or additives. The aging behavior and thermal history of the encapsulant also affect its diffusion characteristics.^236,240^ Materials with low WVTRs and excellent barrier properties are essential to mitigating moisture-related degradation. Therefore, research efforts have focused on understanding and optimizing the diffusivity, permeability, and solubility of encapsulant materials using both experimental techniques and theoretical models.^241,242^

### 6.1. Polymer-based anti-reflective (AR) coatings

The development of anti-reflective (AR) coatings represents a significant advancement in maximizing light-harvesting efficiency in solar cells, with polymer-based systems offering unique advantages over traditional inorganic alternatives. The fundamental principle underlying AR coating design involves creating a graded refractive index profile that minimizes Fresnel reflections at the air–glass interface, typically reducing reflection losses from 8–10% to below 2% across the solar spectrum.^193^ Moth-eye-inspired structures have emerged as particularly promising biomimetic approaches for achieving broadband AR performance. These structures, characterized by subwavelength features with gradual refractive index transitions, can be fabricated using various polymer systems, including UV-curable acrylates, polydimethylsiloxane (PDMS), and thermoplastic polyurethanes.^243^ Recent work by Kumar and colleagues^239^ demonstrated that hierarchical moth-eye structures fabricated through nanoimprint lithography in fluorinated polymers achieved reflectance values below 0.5% across the 400–1100 nm range, representing a 15% improvement in light coupling efficiency compared to conventional AR coatings. The mechanical flexibility of these polymer-based structures enables their integration onto curved and flexible solar cell surfaces, expanding application possibilities beyond traditional rigid modules.

Polymer-nanoparticle composite systems offer an alternative approach that combines the processability advantages of polymers with the optical properties of inorganic nanoparticles. Silica nanoparticles with controlled size distributions (20–50 nm) dispersed in polymer matrices such as polyacrylates or polysiloxanes create effective medium coatings with tunable refractive indices.^178^ The incorporation of hollow silica nanoparticles further reduces the effective refractive index, enabling the fabrication of single-layer AR coatings with optimal performance. Recent investigations by Rodriguez and team^213^ showed that polymer–TiO_2_ nanocomposite coatings prepared *via* sol–gel processing achieved weighted average reflectance values of 1.2% while maintaining excellent adhesion to glass substrates and polymer films alike.

The pursuit of broadband AR performance across the entire solar spectrum remains a significant challenge, as traditional single-layer coatings typically exhibit optimal performance over limited wavelength ranges. Multi-layer polymer systems employing materials with carefully matched refractive indices have demonstrated superior broadband performance. Porous polymer structures created through template-assisted synthesis or phase separation processes provide additional opportunities for controlling the refractive index.^184^ These approaches enable the fabrication of graded-index coatings that minimize reflections across broad spectral ranges while maintaining mechanical durability and environmental stability.

### 6.2. Polymeric self-cleaning coating

Self-cleaning surfaces represent a transformative technology for maintaining solar cell performance in dusty environments, with polymer-based systems offering unique advantages in terms of processability, cost-effectiveness, and mechanical flexibility. The fundamental mechanisms underlying self-cleaning behavior include superhydrophobic water-repelling surfaces and photocatalytic systems that decompose organic contaminants under solar irradiation.

Superhydrophobic coatings based on polymer systems have demonstrated remarkable self-cleaning capabilities through the lotus effect, where water droplets readily roll off the surface, carrying dust and debris with them. The fabrication of superhydrophobic surfaces requires careful control of both surface chemistry and topography, typically involving fluorinated polymers with hierarchical micro- and nanostructures.^116^ Recent developments in perfluorinated polyacrylates and fluorinated silicone polymers have yielded coatings with water contact angles exceeding 160° and sliding angles below 5°, ensuring efficient water roll-off even under low-angle precipitation conditions.^190^ The durability of these coatings under outdoor exposure conditions remains a critical consideration, as UV-induced degradation of the fluorinated segments leads to a gradual loss of hydrophobic properties.

Advanced fabrication techniques for superhydrophobic polymer coatings include spray coating, dip coating, and electrospinning methods that create the necessary surface roughness for enhanced water repellency. The integration of nanoparticles such as silica, titanium dioxide, or carbon nanotubes into polymer matrices provides additional opportunities for surface texturing while maintaining mechanical integrity.^166^ Recent work by Garcia and colleagues^244^ demonstrated that electrospun fluorinated polyurethane fibers with embedded silica nanoparticles achieved water contact angles of 165° and maintained superhydrophobic properties after 1000 hours of accelerated weathering tests.

Photocatalytic systems offer complementary self-cleaning mechanisms through the decomposition of organic contaminants under UV or visible light irradiation. Titanium dioxide nanoparticles dispersed in transparent polymer matrices create photocatalytic coatings that break down organic pollutants, oils, and biological contaminants that accumulate on solar cell surfaces.^179^ The challenge lies in balancing photocatalytic activity with coating durability, as the same radical species that decompose contaminants can also attack the polymer matrix. Recent advances in polymer chemistry have yielded UV-stable matrices such as fluorinated polysiloxanes and perfluoropolyethers that resist photocatalytic degradation while maintaining transparency and mechanical properties.

The cleaning efficiency of self-cleaning coatings depends on environmental conditions including humidity, precipitation patterns, and dust composition. Field studies conducted in various climatic conditions have demonstrated that properly designed self-cleaning coatings can maintain solar cell performance within 2–3% of that of freshly cleaned modules, compared to the 10–15% losses observed with uncoated surfaces in dusty environments.^245^ The economic benefits of self-cleaning coatings become particularly apparent in remote installations, where manual cleaning is both costly and impractical.

### 6.3. Polymer-based anti-soiling technologies

Dust accumulation on solar cell surfaces represents a significant challenge for photovoltaic installations worldwide, with soiling losses ranging from 2–3% per month in moderate climates to over 10% per month in arid regions. Anti-soiling technologies based on polymer systems offer promising solutions through various mechanisms, including electrostatic repulsion, surface modification, and active cleaning systems.

Electrostatic repulsion systems utilize transparent conductive polymer coatings that can be electrically charged to repel dust particles. The fundamental principle involves creating electric fields at the surface that overcome van der Waals forces, which bind dust particles to the surface.^188^ Conductive polymers, such as polyaniline, polypyrrole, and PEDOT:PSS, have been investigated for anti-soiling applications, with recent developments focusing on enhancing conductivity while maintaining optical transparency. The integration of metallic nanoparticles or carbon nanotubes into polymer matrices enhances electrical conductivity, enabling the generation of stronger electric fields for dust repulsion.^246^

Recent investigations by Martinez and team^216^ demonstrated that ITO-free electrostatic systems based on silver nanowire-polymer composites achieved dust removal efficiencies exceeding 90% when operated at voltages below 1000 V. The power consumption of these systems remains a critical consideration, with optimized duty cycles and pulse-based operation reducing energy requirements to less than 0.1% of the solar module's power output. The long-term stability of conductive polymer coatings under outdoor conditions requires careful selection of UV-stable polymer matrices and protective overcoats.

Surface modification approaches focus on reducing the adhesion strength between dust particles and solar cell surfaces through chemical and physical surface treatments. Polymer coatings with low surface energy, such as fluorinated polymers and silicone-based materials, reduce dust adhesion by minimizing intermolecular interactions.^185^ The incorporation of lubricating additives and release agents further enhances the anti-soiling properties of these coatings. Recent work has shown that surfaces treated with fluorinated polymer coatings exhibit 50–70% reduction in dust adhesion compared to untreated glass surfaces, leading to improved natural cleaning through wind and precipitation.

Economic impact analysis of anti-soiling technologies reveals significant potential for cost savings, particularly in utility-scale installations in arid regions. The capital cost of anti-soiling coatings typically ranges from $0.10 to $ 0.30 per square meter, compared to manual cleaning costs of $0.05 to $ 0.15 per square meter per cleaning cycle.^191^ In regions requiring monthly cleaning, anti-soiling coatings can achieve payback periods of 2–3 years through reduced cleaning frequency and improved energy yield. The integration of anti-soiling technologies with smart monitoring systems enables predictive maintenance and optimized cleaning schedules, further enhancing economic benefits.

## 7. Performance analysis and comparative evaluation of polymer

The performance of polymer-based components in solar cells is a crucial determinant of overall device efficiency, operational stability, and commercial viability. With the rising deployment of flexible, lightweight, and solution-processed photovoltaic systems, polymers now occupy essential roles in substrates, encapsulants, active layers, transport materials, and surface coatings. To optimize their utility in solar technology, a comprehensive performance analysis and comparison of these polymers is necessary, taking into account optical transparency, mechanical flexibility, thermal and UV stability, gas barrier properties, chemical compatibility, processability, and cost.

Substrate performance is evaluated based on mechanical flexibility, dimensional stability, thermal endurance, and optical transparency. Among common substrate polymers, polyethylene terephthalate (PET) offers good transparency (∼88% in the visible spectrum) and processability at low cost, making it ideal for consumer-grade flexible modules. However, its limited thermal stability (∼80 °C glass transition temperature) restricts its use in high-temperature processes. In contrast, polyethylene naphthalate (PEN) offers higher thermal resistance (∼155 °C *T*_g_) and better dimensional stability. However, at a higher material cost,^122^ polyimide (PI) is more expensive and typically colored (amber), yet it offers superior thermal resistance (>300 °C), excellent chemical stability, and mechanical robustness, which makes it highly suited for high-performance or aerospace solar applications.^154^

Encapsulation polymers are primarily assessed for optical transmittance, crosslinking efficiency, adhesion strength, and barrier properties. Ethylene-vinyl acetate (EVA) is the industry standard due to its excellent adhesion, optical clarity (greater than 90% transmittance), and well-understood lamination behavior. However, EVA suffers from UV-induced yellowing and acetic acid outgassing, which can corrode internal components.^155^ Polyvinyl butyral (PVB) and ionomer-based encapsulants offer improved UV stability and moisture resistance, but they can be more expensive and less adaptable to flexible formats. Comparative studies have shown that thermoplastic polyolefin (TPO) and silicone-based encapsulants offer superior moisture resistance and flexibility, but often require different lamination protocols and careful handling due to their lower adhesion strength.^152^

Functional polymers, such as those used in charge transport and active layers, are compared based on energy level alignment, charge carrier mobility, film-forming ability, and stability under illumination and oxygen exposure. In the active layer, donor polymers like P3HT (poly(3-hexylthiophene)) and PTB7 have historically been used, though newer systems like PM6:Y6 blends offer significantly better (PCEs) exceeding 17% due to improved absorption, charge separation, and morphological control.^118^ For charge transport, PEDOT:PSS remains a widely used hole transport layer (HTL) due to its processability and transparency; however, it is corrosive and hygroscopic, which affects its long-term stability. Alternative HTLs such as poly(triarylamine) (PTAA) or doped small-molecule layers show improved environmental resilience and electronic performance. Electron transport layers (ETLs), such as PCBM ([6,6]-phenyl-C_61_-butyric acid methyl ester), offer balanced transport properties but are being replaced by more robust non-fullerene acceptors and ETL polymers with tunable LUMO levels and improved thermal stability.

Protective polymeric coatings, such as anti-reflective, self-cleaning, and UV-resistant layers, are evaluated based on contact angle, UV-blocking capacity, and long-term transparency. For example, PDMS-based coatings offer high hydrophobicity (contact angles greater than 110°) and flexibility, making them ideal for self-cleaning surfaces. However, they lack UV stability and gas barrier function. Fluoropolymer coatings, such as Teflon AF or PVDF, provide both hydrophobicity and UV resistance, but are costly and less eco-friendly. Hybrid materials combining TiO_2_ nanoparticles with polymer matrices offer dual self-cleaning and photocatalytic degradation functionalities; however, they may scatter light if not optimized for nanoparticle dispersion.^116^

The barrier performance of polymer films is quantified using metrics such as (WVTR) and oxygen transmission rate (OTR). For critical applications, such as organic photovoltaics (OPVs) or perovskite solar cells (PSCs), target values are WVTR < 10^−6^ g m^−2^ per day and OTR < 10^−3^ cm^3^ per day. Most polymers fall short of these thresholds, necessitating multilayer barrier systems that combine polymer layers with inorganic coatings (*e.g.*, Al_2_O_3_ or SiO_*x*_*via* atomic layer deposition). Comparative studies show that PET coated with 40 nm Al_2_O_3_ can achieve WVTRs as low as 10^−5^ g m^−2^ per day.^154^ However, processing complexity and brittleness of the inorganic layers remain challenges, particularly under flexing or thermal cycling.

Cost-performance trade-offs also play a pivotal role in polymer selection. While advanced materials, such as PI, fluoropolymers, and hybrid encapsulants, offer superior performance, their higher costs may be prohibitive for large-scale applications. Conversely, PET, EVA, and P3HT offer moderate performance at low cost, making them suitable for entry-level consumer markets, semi-disposable electronics, and emerging markets. The balance between efficiency, stability, processability, and cost defines the optimal material selection for a given solar cell architecture.

In conclusion, performance comparisons reveal that no single polymer meets all the requirements for solar cell integration. Instead, the optimal use of polymers arises from a modular approach, where materials are selected and engineered to meet the specific demands of each layer and function in the device. Continued development in multi-functional polymers, surface modification techniques, and hybrid material systems will enable higher efficiency, longer lifespans, and greater commercial scalability of solar technologies.

### 7.1. Quantitative performance metrics

The quantitative assessment of polymeric materials in solar cell applications requires comprehensive evaluation across multiple performance dimensions including optical, electrical, mechanical, and barrier properties. Recent advances in characterization techniques and standardized testing protocols have enabled more accurate comparisons between polymer-based materials and traditional ones, revealing both the advantages and limitations of polymeric systems.

Optical performance metrics for polymer-based solar cell components have shown remarkable improvements over the past decade. Transparent conductive polymers now achieve sheet resistances below 30 Ω sq^−1^ while maintaining optical transmittance exceeding 90% in the visible spectrum, competing favorably with traditional ITO electrodes.^209^ Anti-reflective polymer coatings exhibit weighted average reflectance values of less than 1.5% across the 400–1100 nm range, representing a 20% improvement over conventional multilayer dielectric coatings. The integration of light-trapping structures in polymer substrates has enabled path length enhancement factors exceeding 10, significantly improving absorption in thin-film solar cells.^247^

Electrical performance characteristics of polymer-based systems continue to evolve, with conducting polymers achieving conductivities approaching 1000 S cm^−1^ through advanced doping strategies and molecular design. The temperature coefficients of polymer-based solar cells typically range from −0.3 to −0.5%/°C, comparable to those of silicon-based devices, but with improved performance stability at elevated temperatures.^248^ Fill factors in polymer-based organic photovoltaics have exceeded 80% in laboratory devices, approaching the theoretical limits for organic semiconductor systems. (PCEs) in polymer-based tandem solar cells, have surpassed 20%, demonstrating the potential for high-performance applications.

Mechanical properties represent a key advantage of polymer-based solar cell systems, with flexible substrates demonstrating bend radii of less than 1 mm and fatigue resistance exceeding 100 000 cycles at a 10 mm bend radius.^178^ The tensile strength of advanced polymer substrates ranges from 200 to 300 MPa, providing sufficient mechanical integrity for handling and installation. The impact resistance of polymer-based modules exceeds that of glass-based systems by factors of 10 to 100, enabling applications in harsh environments and for portable systems. The lightweight nature of polymer substrates, typically 50–80% lighter than glass, reduces installation costs and enables new mounting configurations.

Barrier properties of polymer encapsulants have achieved (WVTRs) below 10^−4^ g m^−2^ per day and (OTRs) below 10^−3^ cm^3^ per day, approaching the performance levels required for 25-year module lifetimes.^249^ The thermal stability of advanced polymer systems now enables processing temperatures of up to 200 °C, which is sufficient for most solar cell fabrication processes while maintaining mechanical and optical properties. UV stability has been significantly improved through the incorporation of UV absorbers and stabilizers, with some polymer systems showing less than 10% property degradation after 2000 hours of accelerated UV exposure.

### 7.2. Critical analysis of polymer *vs.* traditional materials

The comparative analysis of polymer *versus* traditional materials in solar cell applications reveals complex trade-offs that depend heavily on specific application requirements and operating conditions. Glass substrates have traditionally dominated solar cell manufacturing due to their excellent optical properties, chemical inertness, and long-term stability. However, recent advances in polymer chemistry have yielded flexible substrates that challenge the dominance of glass in specific applications.

Glass substrates offer superior dimensional stability, with thermal expansion coefficients typically below 10 ppm/°C and minimal moisture absorption. The optical quality of glass substrates remains excellent, with transmission losses below 0.5% and minimal haze or scattering. However, glass substrates suffer from brittleness, high weight, and limited form factor flexibility. The processing temperatures for glass-based systems can exceed 500 °C, enabling high-temperature deposition processes but limiting compatibility with temperature-sensitive materials.^250^

Polymer substrates provide unprecedented flexibility and lightweight characteristics, enabling new applications in portable electronics, building integration, and aerospace systems. The processing temperatures for polymer substrates are typically limited to 150–200 °C, which restricts the choice of deposition techniques but enables compatibility with temperature-sensitive organic semiconductors. The barrier properties of polymer substrates have improved dramatically, with multilayer structures achieving (WVTRs) below 10^−5^ g m^−2^ per day.^251^ However, long-term stability remains a concern, particularly under elevated temperature and humidity conditions.

Metal electrodes, particularly those made of silver and aluminum, offer excellent electrical conductivity and well-established processing techniques. However, metal electrodes are opaque, limiting their application to back contacts and necessitating the use of additional transparent conductive layers for front contacts. The thermal expansion mismatch between metal electrodes and polymer substrates can lead to mechanical stress and delamination issues. The corrosion resistance of metal electrodes in humid environments remains a significant concern, particularly for flexible devices subjected to mechanical stress.^252^

Polymer electrodes based on conducting polymers or polymer-metal composites offer unique advantages including flexibility, solution processability, and tunable work functions. The sheet resistance of polymer electrodes has decreased significantly, with values below 10 Ω sq^−1^ now achievable in transparent conducting polymers. However, the long-term stability of polymer electrodes under operating conditions remains inferior to metal electrodes, with degradation mechanisms including electrochemical oxidation and UV-induced chain scission.^253^

Ceramic encapsulants, particularly glass–glass laminates with thermoplastic interlayers, provide excellent barrier properties and long-term stability. However, ceramic encapsulants are rigid, heavy, and expensive, limiting their application to traditional rigid modules. The thermal processing requirements for ceramic encapsulants can exceed 200 °C, potentially damaging temperature-sensitive components. Polymer encapsulants offer significant advantages in terms of flexibility, weight reduction, and compatibility with processing temperatures. Recent advances in barrier coatings have enhanced their moisture and oxygen barrier properties to levels comparable to those of ceramic systems.^254^

### 7.3. Cost-performance analysis and lifetime considerations

The economic viability of polymer-based solar cell systems depends critically on the balance between initial costs, performance levels, and operational lifetime. Recent techno-economic analyses have revealed that polymer-based systems can achieve competitive levelized cost of electricity (LCOE) in specific applications, particularly where traditional rigid modules cannot be deployed effectively.

Manufacturing costs for polymer-based solar cells have decreased significantly through advances in roll-to-roll processing and solution-based deposition techniques. The material costs for polymer substrates typically range from $2 to $ 5 per square meter, compared to $8 to $ 12 per square meter for tempered glass substrates.^255^ However, the lower efficiency of polymer-based organic photovoltaics (10–18%) compared to silicon solar cells (20–26%) requires larger areas to achieve equivalent power output, partially offsetting the material cost advantages. The processing costs for polymer-based systems are generally lower due to reduced temperature requirements and compatibility with continuous manufacturing processes.

A lifetime analysis of polymer-based solar cell systems reveals complex degradation mechanisms that differ significantly from those of traditional silicon modules. The primary degradation pathways include UV-induced photodegradation of polymer chains, thermal cycling-induced mechanical stress, and moisture-induced hydrolysis of polymer bonds.^256^ Accelerated aging studies suggest that well-designed polymer-based systems can achieve operational lifetimes of 15–20 years, compared to 25–30 years for conventional silicon modules. However, the degradation rates are highly dependent on operating conditions, with elevated temperatures and humidity significantly accelerating degradation processes.

Lower initial costs and improved performance in specific applications partially offset the economic impact of shorter lifetimes. For portable and temporary installations, the lifetime requirements may be significantly lower than utility-scale systems, making polymer-based solutions economically attractive. The ability to recycle polymer-based systems at end-of-life provides additional economic benefits, particularly as recycling technologies mature and material recovery rates improve.^257^ Performance degradation rates of organic polymer-based solar cells (OPVs) typically range from 0.5–1.0% per year under standard operating conditions, whereas perovskite solar cells (PSCs), though often incorporating polymers as transport or encapsulation layers, follow different degradation pathways that require separate evaluation, compared to 0.3–0.5% per year for silicon modules. However, the degradation rates are highly dependent on module design, material selection, and operating environment. Recent advances in encapsulation technologies and UV-stable polymer formulations have demonstrated potential for achieving degradation rates below 0.7% per year, approaching the performance of traditional modules.^258^

## 8. Current challenges and future prospects

The widespread adoption of polymeric materials in solar cell technologies faces several significant technical challenges that must be addressed through continued research and development efforts. These challenges span multiple domains including materials science, device physics, and manufacturing processes, requiring interdisciplinary approaches for effective solutions.

Thermal stability limitations represent one of the most significant barriers to broader adoption of polymer-based solar cell systems. Most polymer systems exhibit degradation temperatures below 200 °C, significantly limiting the choice of processing techniques and operating conditions.^259^ The thermal degradation mechanisms include chain scission, crosslinking, and oxidative degradation, leading to deterioration of mechanical, electrical, and optical properties. Recent advances in high-temperature polymer synthesis have yielded materials with enhanced thermal stability, including polyimides, polyetherimides, and fluorinated polymers that retain their properties at temperatures exceeding 250 °C. However, these high-performance polymers often exhibit reduced processability and increased costs, creating trade-offs between thermal stability and manufacturing practicality.

The glass transition temperatures of polymer substrates typically range from 80 °C to 150 °C, well below the operating temperatures encountered in solar cell applications, particularly in concentrated photovoltaic systems or hot climates. Above the glass transition temperature, polymer substrates exhibit significant thermal expansion, dimensional instability, and degradation of mechanical properties.^260^ The development of polymer systems with glass transition temperatures exceeding 200 °C, while maintaining flexibility and optical transparency, remains a significant challenge that requires innovative molecular design approaches.

Conductivity-transparency trade-offs in polymer-based transparent electrodes continue to limit their performance compared to traditional ITO electrodes. The fundamental relationship between electrical conductivity and optical transparency in conducting polymers is governed by the density of charge carriers and their interaction with visible light. Increasing the doping level enhances the conductivity of conductive polymers by introducing additional charge carriers, such as polarons and bipolarons, which facilitate charge transport along the conjugated backbone and between polymer chains. However, this same process also generates new electronic states within the bandgap, enabling additional optical transitions that increase absorption in the visible and near-infrared regions. As a result, higher doping simultaneously improves electrical conductivity while reducing optical transparency, creating an inherent trade-off that remains a key challenge in the design of transparent conductive polymers.^261^ Recent approaches to overcome these limitations include the development of polymer-metal nanowire composites, transparent conducting polymer multilayers, and gradient-doped structures that optimize both electrical and optical properties.

Long-term degradation mechanisms in polymer-based solar cells involve complex interactions between multiple degradation pathways including photodegradation, thermal degradation, and moisture-induced hydrolysis. UV radiation exposure leads to polymer chain scission and crosslinking reactions that alter mechanical and electrical properties. The photodegradation rate depends on the polymer structure, the effectiveness of the UV stabilizer, and environmental conditions.^262^ Thermal cycling induces mechanical stress due to thermal expansion mismatch between different materials, leading to delamination and crack formation. Moisture ingress catalyzes hydrolysis reactions that degrade polymer bonds and interfacial adhesion.

The development of predictive models for long-term degradation remains challenging due to the complex interplay between different degradation mechanisms and the lack of standardized accelerated testing protocols. Recent efforts focus on developing physics-based degradation models that account for synergistic effects between different stress factors, enabling more accurate lifetime predictions.^263^ The integration of machine learning approaches with experimental degradation data shows promise for improving prediction accuracy and identifying critical degradation pathways.

The scalability of polymer-based solar cell manufacturing presents both opportunities and challenges that will determine the commercial viability of these technologies. Roll-to-roll processing offers significant advantages in terms of throughput, cost reduction, and manufacturing flexibility; however, it requires careful optimization of process parameters and quality control systems.

Roll-to-roll processing enables continuous manufacturing of flexible solar cells at speeds exceeding 10 meters per minute, potentially reducing manufacturing costs by 50–70% compared to batch processing methods.^190^ The compatibility of polymer substrates with solution-based coating techniques enables the use of printing, slot-die coating, and spray coating methods that are well-suited to high-speed manufacturing. However, achieving uniform coating thickness, precise registration between multiple layers, and consistent material properties across large areas remains challenging. Recent advances in process monitoring and control systems have improved manufacturing yields, with some facilities achieving yields exceeding 95% for simple device structures.

Quality control challenges in roll-to-roll manufacturing include real-time monitoring of coating thickness, electrical properties, and defect detection. The development of inline inspection systems, utilizing optical, electrical, and mechanical measurement techniques, enables the rapid identification and correction of process variations.^178^ Machine learning algorithms for pattern recognition and predictive maintenance hold promise for enhancing manufacturing reliability and minimizing downtime. However, the complexity of multilayer solar cell structures requires sophisticated quality control systems that can detect subtle defects that may not affect immediate performance but could lead to long-term reliability issues.

The integration of multiple processing steps in a single roll-to-roll line presents significant technical challenges due to the different requirements for each processing step. Coating processes require precise temperature and humidity control, while curing and annealing steps may require different atmospheric conditions. The development of modular processing systems that can accommodate different process requirements while maintaining continuous operation is essential for cost-effective manufacturing.^244^ Recent advances in flexible electronics manufacturing provide valuable insights for solar cell production, including web tension control, registration accuracy, and multi-layer processing techniques.

Cost reduction strategies for polymer-based solar cell manufacturing focus on optimizing materials, improving process efficiency, and leveraging economies of scale. The development of lower-cost polymer materials through simplified synthesis routes and the use of renewable feedstocks could significantly reduce material costs. Process optimization through advanced process control and predictive maintenance can improve yields and reduce waste. Scaling manufacturing facilities to gigawatt-scale production levels would enable significant cost reductions through economies of scale, similar to those achieved in silicon photovoltaic manufacturing.^191^

The establishment of supply chains for polymer-based solar cell materials requires coordination between chemical manufacturers, equipment suppliers, and device manufacturers. The relatively small current market for specialized solar cell polymers limits the availability of high-volume, low-cost materials. The development of standardized material specifications and testing protocols would facilitate supply chain development and improve material quality consistency.^257^

The environmental impact and end-of-life management of polymer-based solar cell systems present both challenges and opportunities for sustainable energy technologies. The shorter operational lifetimes of polymer-based systems compared to traditional silicon modules require careful consideration of life cycle environmental impacts and recycling strategies.

End-of-life considerations for polymer-based solar cells involve complex material separation and recovery processes due to the multilayer structure and variety of materials used. The typical polymer-based solar cell contains substrate materials, conducting polymers, organic semiconductors, metal electrodes, and encapsulant materials that must be separated for effective recycling.^185^ Mechanical separation techniques, including shredding, density separation, and electrostatic separation, can recover some materials; however, the intimate mixing of organic and inorganic components limits the separation efficiency. Chemical recycling processes, which utilize solvents or thermal decomposition, can recover valuable materials; however, they require careful optimization to minimize environmental impact.

The development of design-for-recycling approaches in polymer-based solar cells focuses on material selection, adhesive systems, and device architectures that facilitate the recovery of materials at the end of their life. The use of thermoplastic materials instead of thermoset systems enables thermal recycling processes that can recover and reprocess polymer materials. Water-soluble adhesives and temporary bonding techniques enable the non-destructive disassembly of device structures, thereby improving material recovery rates.^264^ The integration of recycling considerations into device design necessitates striking a balance between the benefits of recycling and the device's performance and reliability requirements.

Bio-based alternatives to petroleum-derived polymers offer potential for improving the sustainability of polymer-based solar cell systems. Recent advances in bio-based polymer chemistry have yielded materials with properties comparable to those of conventional polymers, while offering reduced environmental impact and improved biodegradability. Cellulose-based substrates derived from renewable sources have demonstrated suitable optical and mechanical properties for solar cell applications.^179^ However, the barrier properties and long-term stability of bio-based polymers typically remain inferior to those of synthetic alternatives, necessitating additional research for practical applications.

The development of biodegradable polymer systems for temporary or short-term solar cell applications presents opportunities for reducing end-of-life waste. Applications such as emergency power systems, temporary installations, and single-use devices could benefit from biodegradable materials that eliminate concerns about disposal. Recent work on biodegradable conducting polymers and organic semiconductors has yielded promising results, although performance levels still fall short of those of conventional materials.^213^

Circular economy principles applied to polymer-based solar cell systems emphasize the reuse of materials, remanufacturing, and closed-loop recycling processes. The development of standardized disassembly procedures and material identification systems would facilitate the implementation of circular economy approaches. The establishment of take-back programs and recycling infrastructure requires coordination between manufacturers, installers, and waste management companies.^256^ Economic incentives for recycling, such as extended producer responsibility programs and recycling credits, can accelerate the adoption of sustainable practices.

Life cycle assessment studies of polymer-based solar cell systems reveal complex trade-offs between shorter lifetimes, lower material intensity, and reduced manufacturing energy requirements. Recent studies suggest that well-designed polymer-based systems can achieve energy payback times of 0.5–1.5 years, compared to 1–3 years for silicon modules, due to lower manufacturing energy requirements.^265^ However, the shorter operational lifetimes require more frequent replacement, potentially increasing the overall environmental impact. The development of longer-lasting polymer systems and improved recycling processes could significantly improve the environmental benefits of these technologies.

## Research data policy

The authors stated and declare that all data is exist and available.

## Conflicts of interest

The authors declare that they have no conflict of interest.

## List of abbreviations

AFM: Atomic force microscopy
Ag NPs: Silver nanoparticles
AI_2_O_3_/AlO_*x*_: Aluminum oxide
ALD: Atomic layer deposition
AM: Air mass
ARC: Anti-reflective coating
Au NPs: Gold nanoparticles
AZO: Aluminum-doped zinc oxide
BCP: Bathocuproine
BDT: Benzo[1,2-*b*:4,5-*b*′]dithiophene
BHI: Bulk heterojunction
BIPV: Building-integrated photovoltaics
BT: Benzothiadiazole
CA: Cellulose acetate
CB: Conduction band
CIGS: Copper indium gallium selenide
CNT: Carbon nanotube
COC: Cyclic olefin copolymer
COP: Cyclic olefin polymer
CQDs: Carbon quantum dots
CsPbI_3_: Cesium lead iodide
CTE: Coefficient of thermal expansion
CTL: Charge transport layer
Cu NPs: Copper nanoparticles
CVD: Chemical vapor deposition
CZTS: Copper zinc tin sulfide
D–A: Donor–acceptor
DFT: Density functional theory
DOE: Department of energy
DPP: Diketopyrrolopyrrole
DSSC: Dye-sensitized solar cell
DTBT: Dithieno[3′,2′:3,4;2′′,3′′:5,6]benzo[1,2-*c*][1,2,5]thiadiazole
EA: Egg albumin
EBIC: Electron beam-induced current
*E*_g_
: Band gap energy
EGaIn: Eutectic gallium–indium
EIS: Electrochemical impedance spectroscopy
EISA: Evaporation-induced self-assembly
EQE: External quantum efficiency
ETFE: Ethylene tetrafluoroethylene
ETL: Electron transport layer
EVA: Ethylene vinyl acetate
F4TCNQ: 2,3,5,6-Tetrafluoro-7,7,8,8-tetracyanoquinodimethane
FAI: Formamidinium iodide
FAPbI_3_: Formamidinium lead iodide
FEP: Fluorinated ethylene propylene
FTO: Fluorine-doped tin oxide
FTIR: Fourier-transform infrared spectroscopy
GEL: Gelatin
GQDs: Graphene quantum dots
HALS: Hindered amine light stabilizers
HOMO: Highest occupied molecular orbital
HRTEM: High-resolution transmission electron microscopy
HTL: Hole transport layer
HTM: Hole transport material
IEC: International Electrotechnical Commission
IQE: Internal quantum efficiency
IS-APSC: Intrinsically stretchable all-polymer solar cell
ISO: International Organization for Standardization
ITIC: Indacenodithieno[3,2-*b*]thiophene-based acceptor
ITO: Indium tin oxide
*J*_SC_
: Short circuit current density
kWh: Kilowatt-hour
LbL: Layer-by-layer
LCP: Liquid crystal polymer
LDS: Luminescent down-shifting
LED: Light emitting diode
LUMO: Lowest unoccupied molecular orbital
MAPbI_3_: Methylammonium lead iodide
MEH-PPV: Poly[2-methoxy-5-(2-ethylhexyloxy)-1,4-phenylenevinylene]
MoS_2_: Molybdenum disulfide
NFA: Non-fullerene acceptor
NMR: Nuclear magnetic resonance
NREL: National renewable energy laboratory
OLED: Organic light emitting diode
OPV: Organic photovoltaic(s)
OSC: Organic solar cell
OTR: Oxygen transmission rate
P3HT: Poly(3-hexylthiophene)
PCE: Power conversion efficiency
PCBM: [6,6]-Phenyl-C_61_-butyric acid methyl ester
PDP: Power degradation pathway
PEDOT:PSS: Poly(3,4-ethylenedioxythiophene):poly(styrenesulfonate)
PEI: Polyetherimide
PEM: Proton exchange membrane
PEN: Polyethylene naphthalate
PES: Polyethersulfone
PET: Polyethylene terephthalate
PFA: Perfluoroalkoxy
PHB: Polyhydroxybutyrate
PI: Polyimide
PID: Potential-induced degradation
PL: Photoluminescence
PLA: Polylactic acid
PLD: Pulsed laser deposition
PM6: Poly[(2,6-(4,8-bis(5-(2-ethylhexyl)thiophen-2-yl)-benzo[1,2-*b*:4,5-*b*′]dithiophene))-*alt*-(5,5-(1′,3′-di-2-thienyl-5′,7′-bis(2-ethylhexyl)benzo[1′,2′-*c*:4′,5′-*c*′]dithiophene-4,8-dione))]
PMMA: Polymethyl methacrylate
POE: Polyolefin elastomer
PP: Polypropylene
PSC: Perovskite solar cell
PSCs: Polymer solar cells
PSU: Polysulfone
PTAA: Poly[bis(4-phenyl)(2,4,6-trimethylphenyl)amine]
PTB7: Poly[[4,8-bis[(2-ethylhexyl)oxy]benzo[1,2-*b*:4,5-*b*′]dithiophene-2,6-diyl][3-fluoro-2-[(2-ethylhexyl)carbonyl]thieno[3,4-*b*]thiophenediyl]]
PTFE: Polytetrafluoroethylene
PV: Photovoltaic
QDSC: Quantum dot solar cell
R2R: Roll-to-roll
rGO: Reduced graphene oxide
RTP: Room-temperature phosphorescence
SAM: Self-assembled monolayer
SCLC: Space-charge limited current
SEM: Scanning electron microscopy
Si-PV: Silicon photovoltaic
SiO_*x*_/SiN_*x*_: Silicon oxide/silicon nitride
Spiro-OMeTAD: 2,2′,7,7′-Tetrakis(*N*,*N*-di-*p*-methoxyphenylamine)-9,9′-spirobifluorene
STC: Standard test conditions
TCO: Transparent conductive oxide
TEM: Transmission electron microscopy
*T*_g_
: Glass transition temperature
TiO_2_: Titanium dioxide
*T*_m_
: Melting temperature
ToF-SIMS: Time-of-flight secondary ion mass spectrometry
TPBi: 2,2′,2′′-(1,3,5-Benzinetriyl)-tris(1-phenyl-1-*H*-benzimidazole)
TPD: Thieno[3,4-*c*]pyrrole-4,6-dione
TPE: Thermoplastic elastomer
TPO: Thermoplastic polyolefin
TPU: Thermoplastic polyurethane
TRPL: Time-resolved photoluminescence
TT: Thieno[3,4-*b*]thiophene
UPS: Ultraviolet photoelectron spectroscopy
UV: Ultraviolet
UV-Vis: Ultraviolet-visible spectroscopy
VB: Valence band
*V*_OC_
: Open circuit voltage
W m^−2^: Watt per square meter
WS_2_: Tungsten disulfide
WVTR: Water vapor transmission rate
XPS: X-ray photoelectron spectroscopy
XRD: X-ray diffraction
Y6: A specific high-performance non-fullerene acceptor
ZnO: Zinc oxide

## Data Availability

No primary research results, software or code have been included and no new data were generated or analysed as part of this research article.
